# Copy number amplification-activated long non-coding RNA LINC00662 epigenetically inhibits BIK by interacting with EZH2 to regulate tumorigenesis in non-small cell lung cancer

**DOI:** 10.7150/jca.69210

**Published:** 2022-03-14

**Authors:** Chunluan Yuan, Yue Ding, Yan Zhuang, Chongguo Zhang, Liang Han, Wei Li, Renhua Guo, Erbao Zhang

**Affiliations:** 1 Department of Oncology, Lianyungang Clinical College of Nanjing Medical University, the First People's Hospital of Lianyungang, PR China; 2 Department of Epidemiology, Center for Global Health, School of Public Health, Nanjing Medical University, Nanjing 211166, China; 3 Department of Oncology, The Affiliated Cancer Hospital of Nanjing Medical University, Nanjing, Jiangsu, 210000, PR China; 4 Department of Oncology, The second Affiliated Hospital of Nanjing Medical University, Nanjing, Jiangsu, 210000, PR China; 5 Department of Oncology, Xuzhou Central Hospital, Xuzhou, Jiangsu, PR China; 6 Department of Oncology, The first Affiliated Hospital of Nanjing Medical University, Nanjing, Jiangsu, 210000, PR China; 7 Department of Oncology, Sir Run Run Hospital, Nanjing Medical University, Nanjing, Jiangsu, 210000, PR China; 8 Jiangsu Key Lab of Cancer Biomarkers, Prevention and Treatment, Collaborative Innovation Center for Cancer Personalized Medicine, Nanjing Medical University, Nanjing 211166, China

**Keywords:** Gene amplification, LINC00662, tumorigenesis, NSCLC

## Abstract

Recently, studies have shown that lncRNAs play important roles in regulation of cancer cells proliferation, apoptosis and metastasis. Here, through systematic bioinformatics analysis and screening, we identified a long noncoding RNA LINC00662 with high copy number amplification in NSCLC. High expression of LINC00662 predicted a poorer survival. The exact sequence full-length of LINC00662 was determined by rapid amplification of cDNA ends (RACE). We also found that LINC00662 could regulate lung cancer cell proliferation both in vitro and in vivo. Mechanically, we obtained global expression profile that respond to LINC00662 knockdown through RNA-Seq analysis. And we found that LINC00662 could bind to EZH2 and recruit EZH2 to the promoter regions of BIK, regulating the level of H3K27me3 in the BIK promoter, thus epigenetically repressing BIK expression. Our results shown that lncRNA LINC00662, driven by copy number amplification, promotes tumorigenesis by EZH2/BIK cell axis, indicating that it was a potential molecular target of NSCLC.

## Introduction

Lung cancer is the main cause of the high incidence of cancer and high mortality worldwide 1, 2. NSCLC accounts for about 80-85% of all lung cancers 3, 4. Although a combination of chemotherapy, radiation treatment, surgical operation, and molecularly targeted therapy approach can be adopted in lung cancer treatment 5, 6. The survival rate of NSCLC patients is remaining poor 7, 8. So, it is necessary to further understand the molecular mechanism of NSCLC to improve the problems in the diagnosis and treatment of NSCLC.

Long noncoding RNA (>200 nt) has gained extensive attention because of the deepening research for noncoding RNAs (ncRNAs) 9. Although lncRNAs were previously considered to be "noise" generated in transcription, now lncRNAs considered to be regulators that play a key role in biological processes including cellular development, differentiation, etc. 9, 10. Recently, much research has indicated that the lncRNAs aberrant expressions can affect many types of tumorigenesis including lung cancer 11-14. For instance, LncRNA HOTAIR could lead to the transcriptional activation of c-MYC target genes in breast cancer cells, thus promoting the occurrence of breast cancer 15. Long noncoding RNA NEAT1 promoted proliferation and invasion of NSCLC cells via targeting miR-181a-5p 16. Our previous work also found that lncRNAs could play key roles in tumorigenesis^14^, ^17^, ^18^.

LINC00662 was a lncRNA that was located in the chromosome 19q11. LINC00662 have revealed its carcinogenesis in oral squamous cell carcinoma, gastric cancer and prostate cancer etc. 19-22. For instance, LINC00662 could increase the abilities of OSCC cell proliferation and invasion by regulating the Wnt/beta-catenin pathway 19. LINC00662 promoted gastric cancer cell proliferation by LINC00662-miR-497-5p-YAP1 axis 20. Published research revealed that the dysregulation of LINC00662 could involve in tumorigenesis. However, the cause for LINC00662 activation and its biological mechanism in lung cancer tumorigenesis, especially, the global genes mediated by LINC00662 has not been established. In addition, the exact sequence of LINC00662 has not been determined. Therefore, we pay attention to its specific role in tumorigenesis of NSCLC.

In the research, we discovered LINC00662 was significantly elevated in NSCLC samples and evaluated poor survival. The activation of LINC00662 is driven by the copy number amplification. Then we first identified the full-length sequence of LINC00662. And we found that knockdown LINC00662 could decrease the ability of cell growth and migration both in vitro and in vivo. Mechanically, we obtained global expression profile that respond to LINC00662 knockdown through RNA-Seq analysis. And we found that LINC00662 could interact with EZH2 and recruit EZH2 to the promoter regions of BIK, regulating the level of H3K27me3 in the BIK promoter, thus epigenetically repressing BIK expression and promoting tumorigenesis of NSCLC. These results indicated that LINC00662 may play important role in the tumorigenesis of NSCLC and could supply a certain theoretical basis of the diagnosis and treatment for NSCLC.

## Materials and Methods

### Lung cancer lncRNA expression profiling data retrieval and analysis

Microarray gene expression data was retrieved from the GEO dataset. The data sets from GSE51852 and GSE43767 were added to our study. We retrieved the original files from the GEO database. Next, the normalization and z-score transformation were performed to the raw data. Then we get the differentially expressed lncRNA.

### The copy number variations and expression analysis of LINC00662

NSCLC copy number variation data obtained from whole genome sequencing and transcriptome sequencing of The Cancer Genome Atlas (TCGA) database. We obtained the expression data of samples from the UCSC Xena website (https://xenabrowser.net/datapages/) and downloaded the copy number data of NSCLC samples from https://gdac.broadinstitute.org/. A threshold of 1 in values was considered an amplification and calculated the ratio of copy amplification for LINC00662.

### Cell Cultures

Two human NSCLC cell lines were purchased from the Institute of Biochemistry and Cell Biology at the Chinese Academy of Sciences (Shanghai, China). A549 cells and SPCA1 cells were cultured in RPMI 1640 and DMEM (Gibco-BRL) containing 10% fetal bovine serum (FBS), respectively, supplemented with 100 U/mL penicillin and 100 mg/mL streptomycin (Invitrogen, Carlsbad, CA, USA). Both A549 and SPCA1 cells were cultured in an environment with a temperature of 37°C and 5% CO_2_.

### RNA isolation and qRT-PCR Analyses

The cultured cells were lysed with TRIzol reagent (Invitrogen, Carlsbad, CA, USA) to extract total RNA. Then the RNA was reverse transcribed with a reverse transcription kit (Takara, China) to obtain cDNA. SYBR Premix Ex Taq (TaKaRa, China) was used to perform real-time PCR analyses, following the manufacturer's instructions. The expression of glyceraldehyde-3-phosphate dehydrogenase (GAPDH) was used for calibration. Specific primers are in Supplementary Table S2.

### RACE (rapid amplification of cDNA ends)

Total RNA was extracted using RNA Purification Kit (Invitrogen) followed the manufacturer's instructions. 5' RACE and 3' RACE was performed using SMART RACE cDNA Amplification Kit (Cat. 634858, Clontech, Palo Alto, CA, USA) according to the manufacturer's instructions.

### Cell transfection

Followed the instructions and used Lipofectamine 2000 (Invitrogen) reagent to transfect LUAD cells together with siRNA. Negative control was siRNA (si-NC) (Invitrogen, CA, USA). All sequences of siRNAs are listed in Supplementary Table S2.

### Flow Cytometric Analysis

After the medium supernatant was collected, the transfected cells were digested with trypsin, and then re-suspended and collected together with the medium supernatant. Annexin V FITC Annexin V Apoptosis assay kit (BD Biosciences) was subsequently used for double staining of luciferin isothiocyanate-Annexin V and propidium iodide. Results were analyzed using FACScan (BD Biosciences) with Cell Quest software. The cells were divided into living cells, dead cells, early apoptotic cells and apoptotic cells. Then calculated and compared the proportion of apoptotic cells in each experimental group. All experiments were performed three parallel.

### EdU Analysis

Cultured the cells in a 24-well plate, the number of cells per well is 5×10^4^, after transfection 48 hours, reagent A from EdU assay kit (Ribobio, Guangzhou, China) prepared in a certain proportion with culture medium and then added to 24-well plate for incubation 2 hours at a temperature of 37°C and a CO2 concentration of 5%. The cells were treated with 4% paraformaldehyde followed by 0.5% Triton X-100, after which the cells were stained with anti-EdU working solution, and the nuclei were labeled with DAPI. After taking pictures through a fluorescence microscope, count and calculate the percentage of EDU-positive cells. Three fields of view were randomly selected for each group.

### Tumor formation assay in mouse model

Athymic BALB/ C nude mice aged 5 weeks were raised under certain pathogen-free conditions. After NC or si-LINC00662 transfected A549 cells, the suspension cells were subcutaneously injected to either side of the rear flank of the nude mice. Tumor volume and weight of mice in control group and si-LINC00662 group were measured. Tumor volume was measured. A few days after the injection, the mice were killed, and the tumors were taken out and the tumors were weighed. Our proposal was approved by the Ethics Committee of Animal Experiments of Nanjing Medical University.

### Transcriptome sequencing

Total RNA was extracted from LINC00662 knockdown A549 cells and control cells. NanoDrop 2000 (Thermo Scientific, USA) was used to mensurate the concentration of each sample. Quality was evaluated by Agilent2200 (Agilent, USA). Based on a solution provided by the manufacturer (Life Technologies, USA), Ion Proton Total RNA-SEQ Kit V2 was used to prepare sequencing libraries for each RNA sample. Data are provided in supplementary Table S1.

### Western blot assays

Transfected cells were harvested 48 h after transfection. Then the protein was extracted and electrophoresed. 10% SDS-PAGE was used to segregate the protein, then incubated together with specific antibodies, and lastly densitometry (Bio-Rad) was used to quantify the electrophoretic bands. The EZH2 antibody (1:1000) were acquired from Abcam (Cat#: ab191250), and the GAPDH served as control (CST, Cat#: 5174).

### RNA immunoprecipitation (RIP) assays

RNA immunoprecipitation (RIP) experiments were performed using a Magna RIP™ RNA-Binding Protein Immunoprecipitation Kit (Millipore, USA) according to the manufacturer's instructions. Antibody for RIP assays of EZH2 (Abcam, Cat#: ab191250) were from Abcam.

### Chromatin immunoprecipitation (ChIP) assays

ChIP assays were performed using EZ-CHIP KIT according to the manufacturer instruction (Millipore). Antibody of EZH2 (Abcam, Cat#: ab191250) and H3K27me3 (Abcam, Cat#: ab6002) were from Abcam. Quantification of immunoprecipitated DNA was performed using qPCR. Result was calculated as a percentage relative to input DNA by equation 2^[Input Ct- Target Ct]^ ×100 (%).

### Statistical analyses

All statistical analyses were performed using SPSS software. Student's t test, χ^2^ test, or Wilcoxon test, as appropriate were used for the difference between the two groups. The OS rates were calculated by the Kaplan-Meier method and the log-rank test was used for comparison. A bilateral P value of 0.05 was considered statistically significant.

## Results

### Copy number amplification could lead to activation of LINC00662 in NSCLC

**Figure 1:**
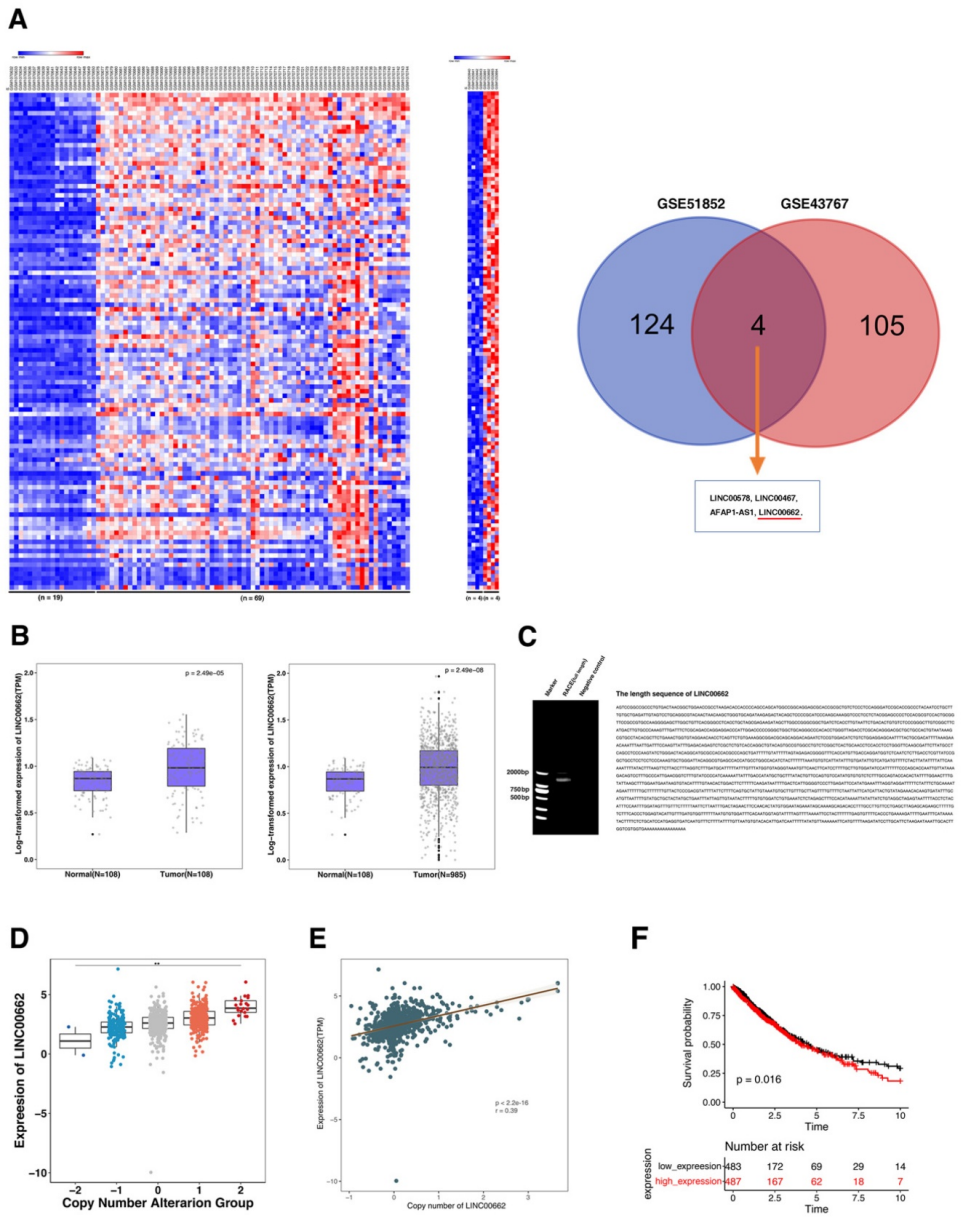
** Copy number amplification drives LINC00662 high expression in NSCLC.** A: Raw microarray data including GSE51852 and GSE43767 were downloaded from GEO and then normalization. B: The expression of LINC00662 was activated in NSCLC tissues with paired and unpaired TCGA data. C: Exact full length of LINC00662 by RACE. D and E: LINC00662 expression was correlated with its copy number level. F: Higher expression of LINC00662 was indicated a poorer overall survival.

Raw microarray data were obtained from GEO, containing GSE51852 (n=4vs4) and GSE43767 (n=19vs69). The normalization was performed to the signal data. As shown in Figure 1A, we obtained activated lncRNA in these two data sets (fold change ≥ 1.5). We can see that there are 4 activated lncRNAs in lung cancer (LINC00578, LINC00467, AFAP1-AS1, LINC00662), among these differentially expressed lncRNAs, there also have many other well-known lncRNAs in lung cancer, such as LINC00578^23^. Our previous studies also found that AFAP1-AS1 could display important regulatory roles in lung cancer tumorigenesis^14^. Based on the basic expression level of in lung cancer, we choose LINC00662 for further in-depth study.

Then we used TCGA database of NSCLC to evaluate the expression of LINC00662 in tumors and normal samples. In Figure 1B, the results indicated that LINC00662 significantly higher expression in NSCLC, which compared with their matched adjacent normal tissues, whether in a matched and unmatched tissues. Then we preformed rapid amplification of cDNA ends (RACE) of LINC00662 in A549 cells. The result showed the exact complete sequence of LINC00662 and its length is 2064nt (Figure 1C). Furthermore, we found that as the copy level increased, the expression of LINC00662 gradually increased (Figure 1D). Moreover, LINC00662 copy number amplification is indeed positively correlated with expression (Figure 1E). This suggested that the increase of LINC00662 expression in NSCLC may partly be due to gene amplification and play an important role in NSCLC tumorigenesis. Further analysis found that the high expression of LINC00662 suggested poor prognosis in NSCLC (Figure 1F).

### LINC00662 regulates lung cancer cell proliferation, migration and cell apoptosis in* vitro.*

**Figure 2:**
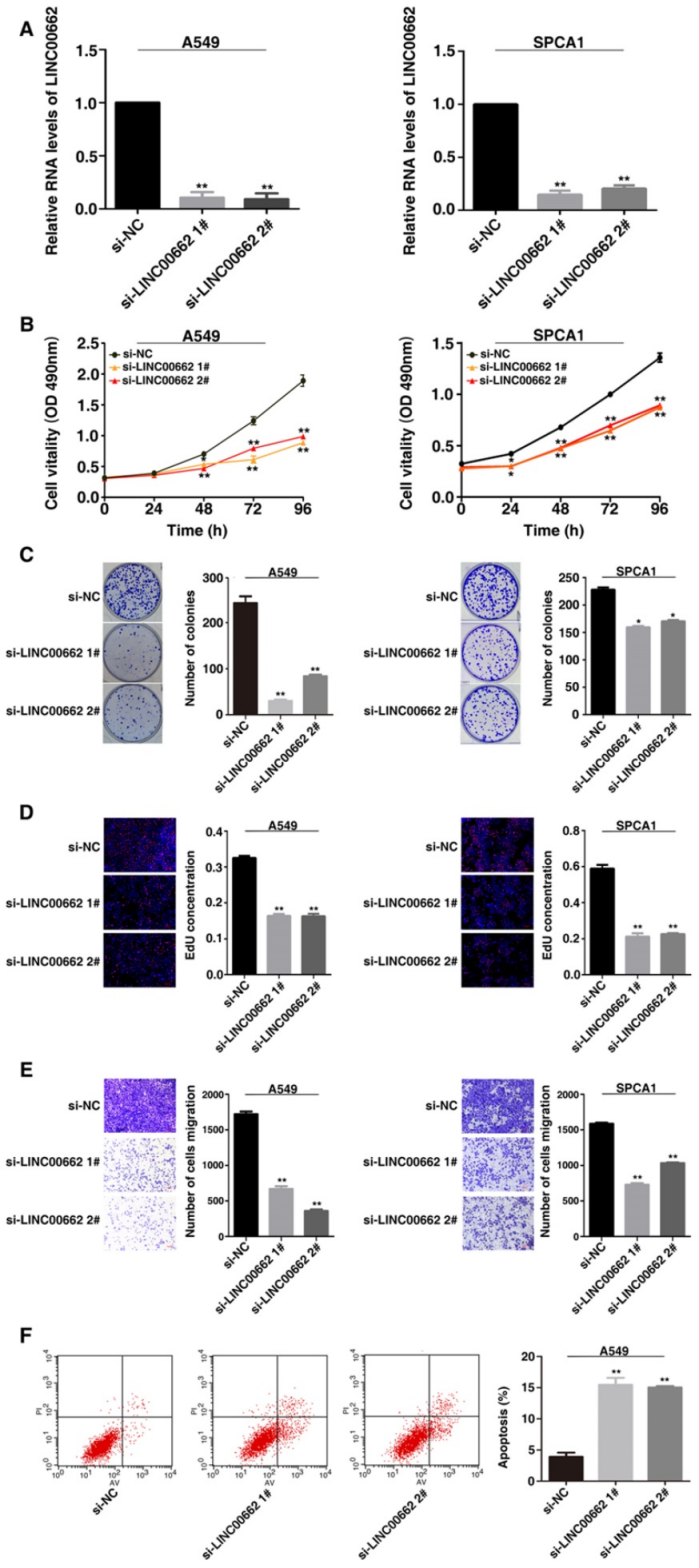
** Knockdown LINC00662 inhibits NSCLC cell proliferation and migration in *vitro*.** A: The expression levels of LINC00662 in siRNA-mediated A549 and SPCA1 cells were detected by qRT-PCR. B: After siRNA-mediated LINC00662 knockdown, the proliferation of A549 and SPCA1 cells were detect by MTT assays.C: Colony formation assays were detected in LINC00662 silenced A549 and SPCA1 cells. D: 48 h after transfection, the proliferation of siRNA-mediated A549 and SPCA1 cells were detected use the EdU staining kit. E: After siRNA transfection the altered migratory capacity of A549 and SPCA1 cells were performed to investigate by transwell assays. F: A549 cells were used flow cytometry to be stained and analyzed. LR: early apoptotic cells. UR: terminal apoptotic cells. *P<0.05, **P<0.01.

**Figure 3:**
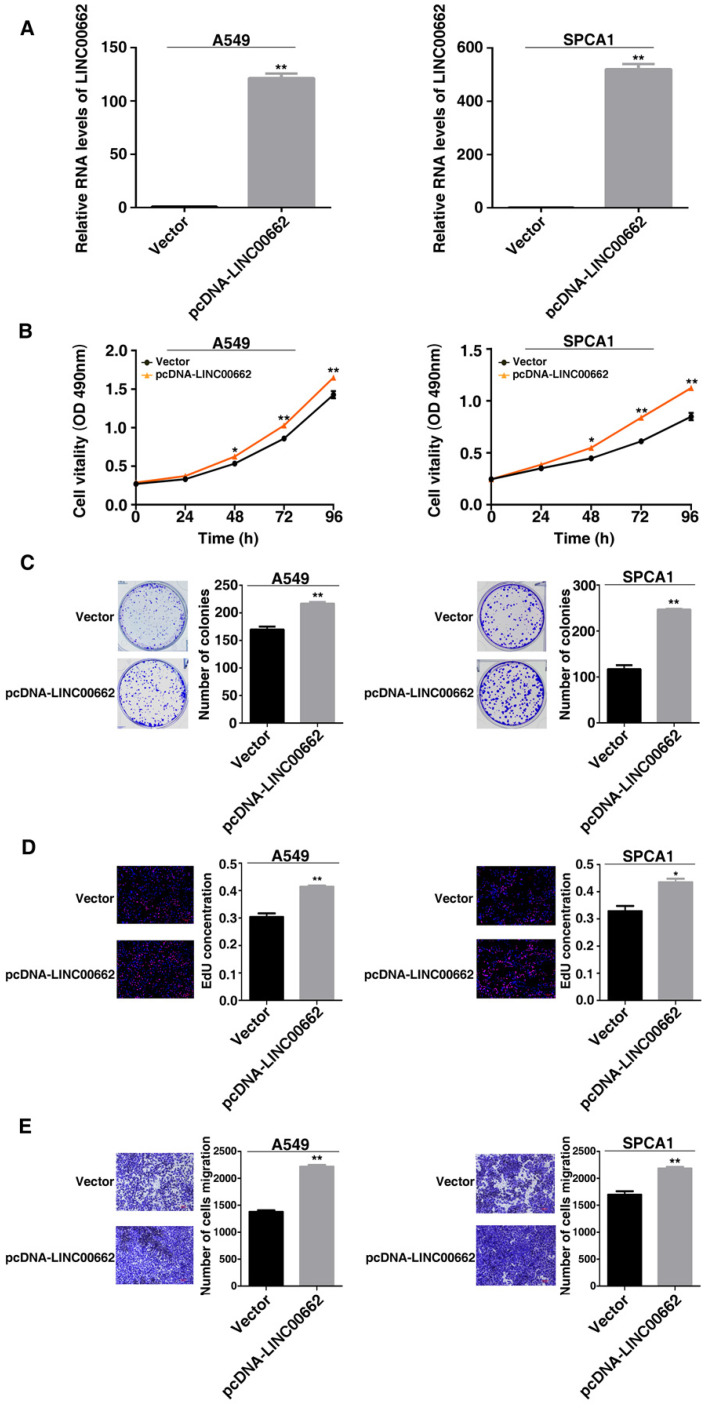
** Overexpression of LINC00662 promotes NSCLC cell proliferation and migration in *vitro*.** A: Analysis of LINC00662 expression levels in A549 and SPCA1 by qRT-PCR. B: After LINC00662 overexpression cell proliferation both in A549 and SPCA1 cells were determine by MTT assays. C: Colony formation ability were detected in A549 and SPCA1 cells after LINC00662 was overexpression. D: The proliferation of A549 and SPCA1 cells were determined after overexpression of LINC00662 by EdU assays. E: The alterations in migratory capacity of A549 and SPCA1 cells were used transwell assays to investigate after transfection. *P<0.05, **P<0.01.

This study selected human lung cancer cell lines A549 and SPCA1 to determine the biological role of LINC00662. Then siRNA was used to mediate silencing of LINC00662 expression and plasmid-mediated overexpression in lung cancer cells (Figure 2A and 3A). Methylthiazol tetrazolium (MTT) assays showed that cell proliferation was inhibited after LINC00662 knockdown in A549 and SPCA1 cells (Figure 2B). And the clone formation experiment results showed that the clone survival were decreased after silencing LINC00662 in A549 and SPCA1 cells (Figure 2C). Consistently, ethynyldeoxyuridine (EdU) staining assays also proved that after silencing LINC00662, the proliferation rate of A549 and SPCA1 were inhibited (Figure 2D). Next, transwell assays revealed the ability of migration of NSCLC cells. As showed in Figure 2E, after silencing of LINC00662, the migration both in A549 and SPCA1 cells were significantly repressed.

Apoptosis plays an important role in the proliferation of various cancer cells. To determine whether apoptosis affects the apoptosis of NSCLC cells, flow cytometry analysis was performed. In Figure 2F, the apoptotic percentage of A549 cells was observably higher than the control group after silencing LINC00662. Therefore, LINC00662 may regulate NSCLC cell proliferation by influencing cell apoptosis.

By contrast, overexpressed LINC00662 promoted cell proliferation and increased the cell clone numbers (Figure 3B and 3C). Similarly, EdU staining assays found the proliferation ability both in A549 and SPCA1 were increased after overexpression of LINC00662 (Figure 3D). And overexpression of LINC00662 promoted cell migration (Figure 3E). The above results indicate that LINC00662 may play a key role in the proliferation and migration of NSCLC cells.

### LINC00662 regulates NSCLC cell proliferation in *vivo*

**Figure 4:**
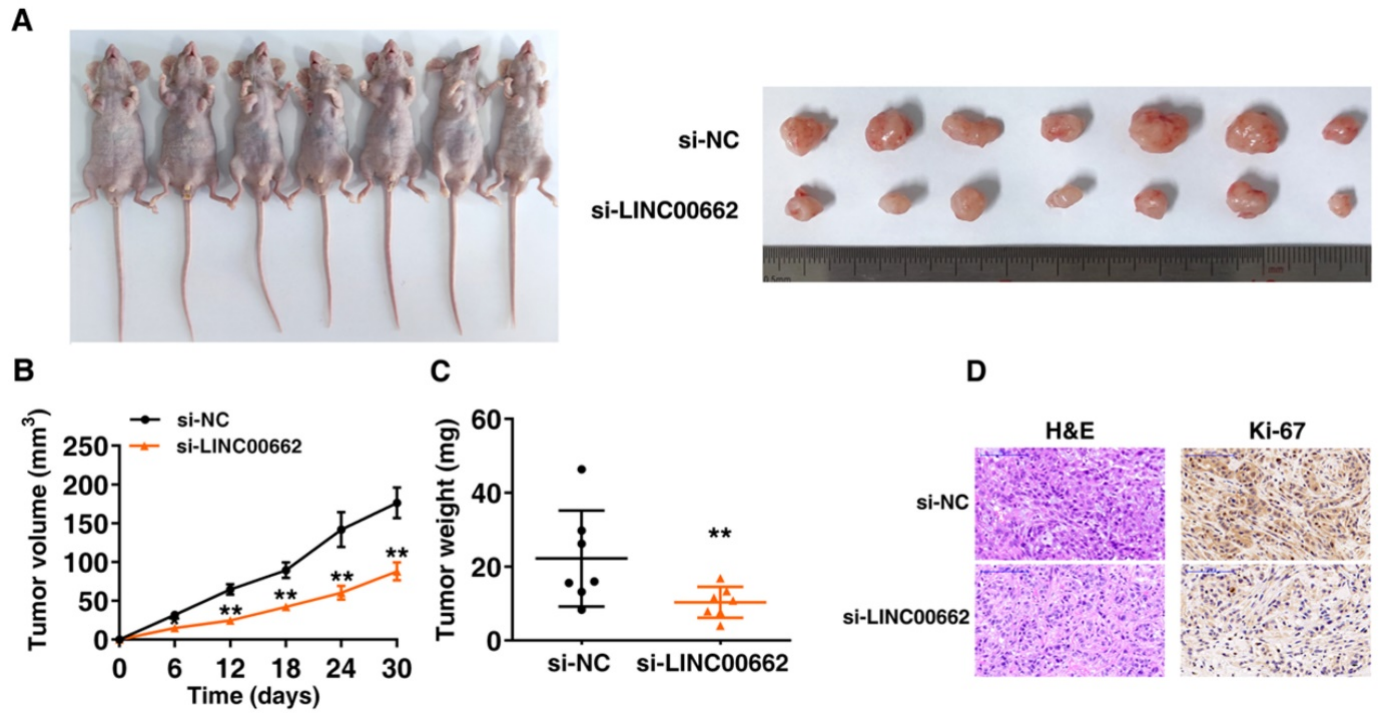
** LINC00662 regulates NSCLC cell proliferation and migration in *vivo*.** A: Transfected A549 cells were injected into nude mice. B: Tumor volume was calculated every 6 days after injection. C: Tumor weights were presented as mean tumor weight ±S.D. (standard deviation). D: H&E staining and IHC staining (antibodies against Ki-67) were performed to detect tumor sections, and it was found that the tumor developed from the siLINC00662 transfection group was down-regulated. *P<0.05, **P<0.01.

Further research is to confirm whether LINC00662 affects lung cancer cell proliferation in *vivo*, nude mice were inoculated with the A549 transfected with control and si-LINC00662 (Figure 4A). After continuous measurement, the tumor volume of the si-LINC00662 group was significantly smaller than that of the control group (Figure 4B). In addition, at the end of experiment, the average tumor weight of the LINC00662 knockdown group was significantly lower than that of the control group (Figure 4C). The positive rate of Ki-67 staining for tumors formed by si-LINC00662-transfected A549 cells was significantly lower than that of the control group (Figure 4D). These above results indicated that LINC00662 knockdown can repress tumor growth in *vivo*. The experiments in *vivo* are consistent with those of in *vitro* functional studies which mediated by LINC00662.

### The global downstream genes regulated by LINC00662

**Figure 5:**
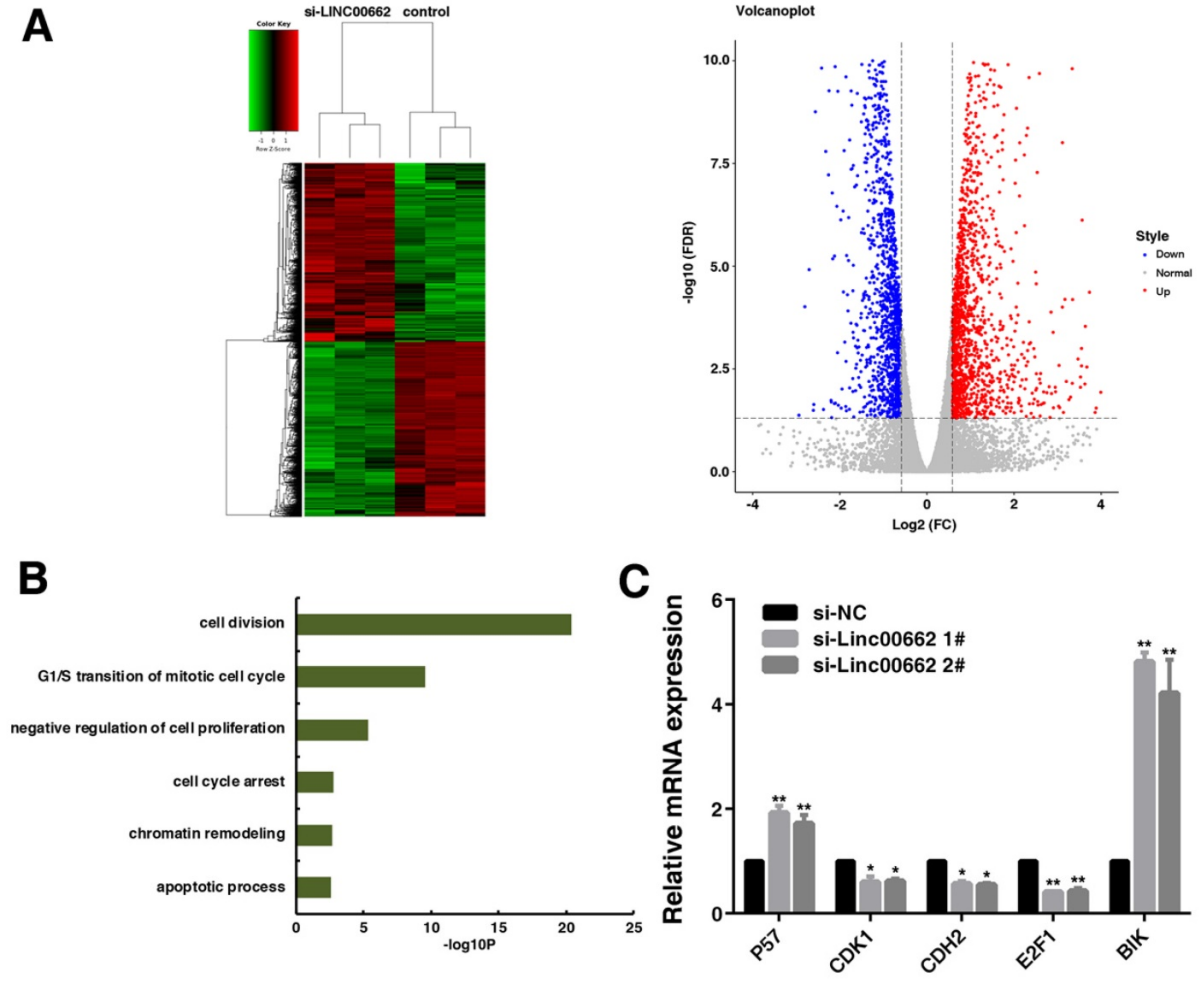
** The global downstream target genes of LINC00662.** A: After LINC00662 was knockdown from A549 cells, the mean center and grading clustering of gene transcripts were changed (≥1.5-fold change), with a total of 3 repeats. This was a volcanic map depicting the up-regulated and down-regulated genes knockdown by LINC00662 in RNA-seq. B: Gene ontology analysis of the whole gene expression changes after LINC00662 gene knockdown. C: qRT-PCR selectively confirmed the changes in mRNA levels after LINC00662 knockdown. *P<0.05, **P<0.01.

To determine changes in global downstream gene expression of LINC00662, we assessed the global effects of LINC00662 knockdown by RNA transcriptomic sequencing in A549 cells (Figure 5A). We totally identified 2934 differentially expressed genes (fold change ≥ 1.5), 1483 genes were up-regulated while 1451 genes were down-regulated in LINC00662 knockdown A549 cells (Supplementary Table S1). The results of GO terms analysis showed that the majority of these genes were involved in cell division, cell cycle and apoptotic process (Figure 5B). We selected five genes (P57, CDK1, CDH2, E2F1 and BIK) that are quite relevant to cancer cell proliferation and apoptosis 24-28. We confirmed their change expression by qRT-PCR after LINC00662 knockdown in A549. After LINC00662 knockdown, CDK1, CDH2 and E2F1 were inhibited, while the expressions of P57 and BIK were induced (Figure 5C). These results suggested that LINC00662 could affect tumorigenesis by regulating these key genes related to tumorigenesis.

### LINC00662 could bind to EZH2 and recruit EZH2 to the promoter regions of BIK, thus epigenetically repressing BIK expression and promoting tumorigenesis of NSCLC

**Figure 6:**
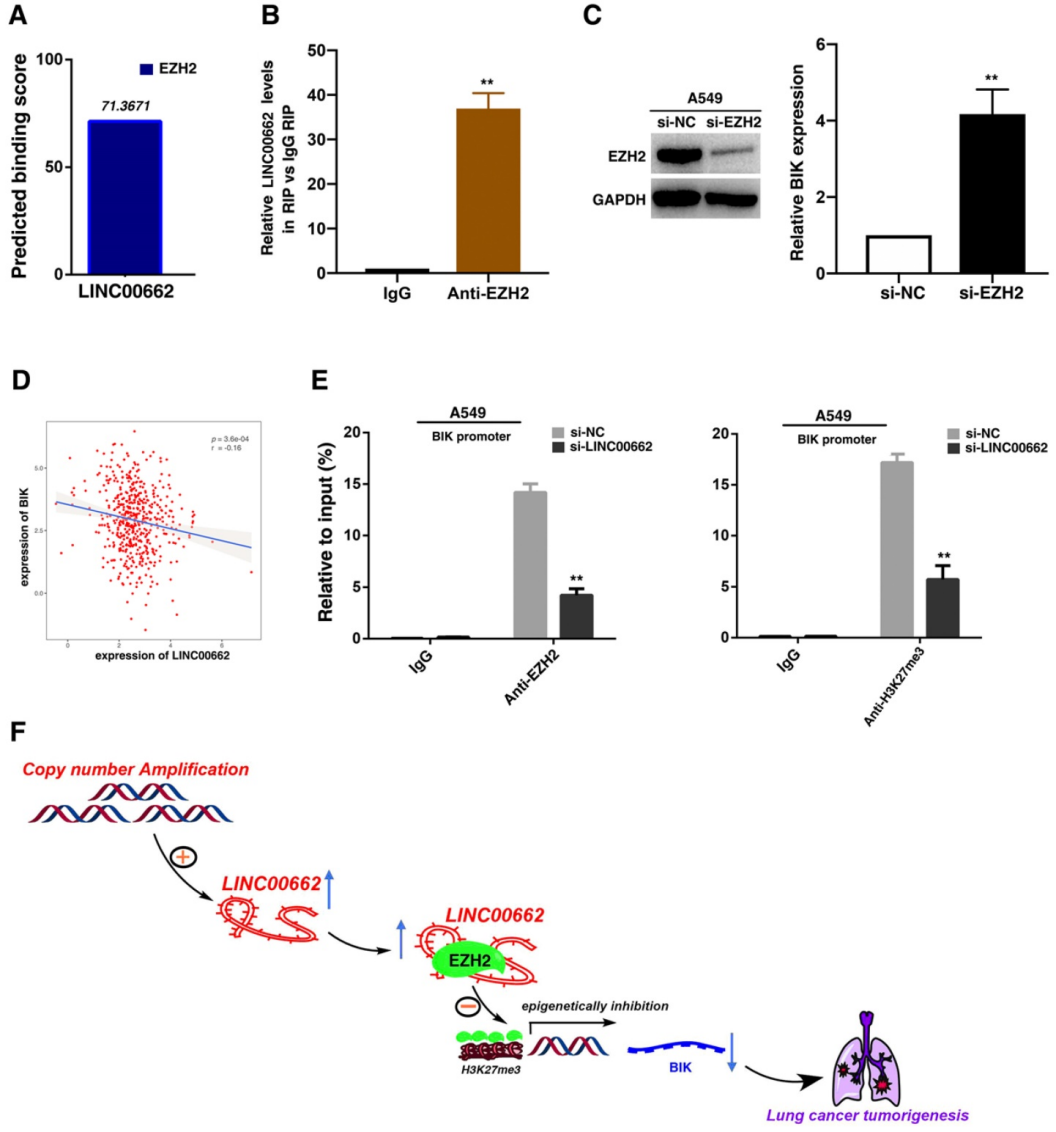
** LINC00662 could bind to EZH2 and recruit EZH2 to the promoter regions of BIK, thus epigenetically repressing BIK expression and promoting tumorigenesis of NSCLC.** A: Bioinformatics were used to predict the possible interaction of LINC00662 and EZH2. B: RIPs experiments were performed, and the coprecipitated RNA was subjected to qRT-PCR for LINC00662. C: The expression of EZH2 after knockdown of EZH2, and the expression of BIK after knockdown of EZH2. D: The level of LINC00662 and BIK showed a statistically negative correlation in TCGA. E: ChIP-qPCR of H3K27me3 and EZH2 of the promoter region of BIK loci after siRNA treatment targeting si-NC or si-LINC00662. Antibody enrichment was quantified relative to the amount of input DNA. An antibody directed against IgG was used as a negative control. F: A proposed model which is mediated by LINC00662.

Recent studies have reported that a significant number of lncRNAs have been shown to function in cooperation with chromatin modifying enzymes to exert epigenetic regulation of gene expression^29^. Approximately 20% of human lncRNAs have been shown to interaction with PRC2, indicating that lncRNAs may have a general role in recruiting PRC2 proteins to their target genes^30^. PRC2, a methyltransferase that is composed of EZH2, SUZ12 and EED, can catalyze the trimethylation of lysine residue 27 of histone 3 (H3K27me3), thus epigenetically repressing gene expression^31^. And abnormal expression of PRC2 is closely related to the occurrence and development of tumors 32. Thus, we hypothesized that LINC00662 may regulate gene expression in such a manner. To test this, we used bioinformatics to predict this interaction possibility (http://bioinfo. bjmu.edu.cn/lncpro/). As shown in Figure 6A, the predicted binding scores between LINC00662 and EZH2 were fairly high. To validate this interaction, RNA immunoprecipitation (RIP) assays showed that the endogenous LINC00662 was enriched in the anti-EZH2 RIP fraction compared to the IgG fraction (Figure 6B). Next, through analysis of sequencing results, we found that BIK was significantly induced after knockdown of LINC00662. Then the role of EZH2 in coregulating suppression of LINC00662-suppressed BIK was investigated by EZH2 knockdown, and both were induced in cells after knockdown of EZH2 (Figure 6C). Further analysis demonstrated that LINC00662 was negatively correlated with BIK expression (Figure 6D). To address whether LINC00662 is involved in epigenetically transcriptional repression by enrichment of EZH2 to target gene promoters, we conducted ChIP analysis afrer LINC00662-knockdown. ChIP assays demonstrated that LINC00662 decreased the binding of EZH2 and H3K27me3 levels across the BIK promoters (Figure 6E). These results indicated that LINC00662 promotes tumorigenesis of NSCLC via epigenetically repressing BIK expression.

## Discussion

Recent research reported that lncRNA was a vital component in regulating the tumorigenesis of lung cancer. In this research, we focused on lncRNA LINC00662 in NSCLC. LINC00662 have revealed its oncogenic property in many tumor types 19-21, 33, however, the reason for how LINC00662 activated in tumorigenesis remain unclear. As an important reason for activation of lncRNA, at the genomic level, copy number amplification has attracted widespread attention. For example, lncRNA PVT1 which located in 8q24 region is activated by amplification in multiple tumor types, and PVT1 could participate in the regulation of tumorigenesis^34^. Our results showed that the activation of LINC00662 in NSCLC part of the reason may be the copy number amplification. In addition, the exact sequence of LINC00662 is still unclear. We firstly used RACE to get its exact sequence in NSCLC.

We also shown that knockdown of LINC00662 could significantly inhibit NSCLC cell proliferation, migration and induce apoptosis. Moreover, we obtained the global downstream genes which mediated by LINC00662. Several of them were related to cell division, cell cycle and apoptosis processes. Then qRT-PCR was used to identify the five potential downstream target genes P57, CDK1, CDH2, E2F1 and BIK after knockdown of LINC00662. These genes may be potential targets of LINC00662 in lung cancer because they could contribute an important role to tumorigenesis 24, 26-28, 35. As an inhibitor of cyclin-dependent kinase, P57 is considered to be a tumor suppressor gene in various tumors 36. It can also inhibit the development of NSCLC by LINC00511-mediated epigenetic silencing 37. Our study confirmed that LINC00662-knockdown can mediate up-regulation of P57. E2F1 could mediate the cell cycle and apoptosis of tumor cells 38. Reports suggested that E2F1 played a key role in the proliferation of tumors cells 39. Its expression was markedly reduced after LINC00662 knockdown. BIK is a member of the BCL-2 family and is considered as a tumor suppressor due to its vital function in promoting apoptosis including lung cancer 38. Studies have proven that activation of BIK can result in cancer cell death. 40. In addition, it has been found that BIK can be induced to increase cell cancer apoptosis, and these regulatory mechanisms may serve as important targets for tumor therapy drugs 41. However, the mechanism by which BIK expression is inhibited is unclear. Our results demonstrated that knockdown of LINC00662 could induce the expression of BIK by EZH2-dependent manner. Many lncRNAs regulate the level of H3K27me3 of specific gene promoter loci via recruiting and binding to EZH2, and EZH2-mediated epigenetically regulation plays a crucial role in process of tumor development^42^.

In summary, we found that LINC00662 was upregulated by copy number amplification in NSCLC, and its exact sequence was determined. LINC00662 is crucial for maintaining NSCLC cell proliferation, apoptosis and migration. In addition, our research also revealed the global downstream genes of LINC00662 for the first time. And LINC00662 could interact with EZH2 and recruit EZH2 to the promoter regions of BIK, thus epigenetically repressing BIK expression and promoting tumorigenesis of NSCLC. Therefore, it is believed that targeting LINC00662 may be an effective strategy to inhibit the development of NSCLC. Our studies have further deepened our understanding of the occurrence of lung cancer and may provide potential molecular targets for NSCLC.

## Supplementary Material

Supplementary tables.
