# Supplementary material for: Copy number amplification-activated long non-coding RNA LINC00662 epigenetically inhibits BIK by interacting with EZH2 to regulate tumorigenesis in non-small cell lung cancer
Source: J Cancer. 2022 Mar 14;13(5):1640–51. doi: 10.7150/jca.69210 (PMC8965125; doi:10.7150/jca.69210)
Supplement: Supplementary file 1 — Supplementary tables. [file jcav13p1640s1.pdf]

Supplementary Table S1 and Table S2

Supplementary Table S1: mRNAs variation abundance (≥1.5-fold) in LINC00662-knockdown A549 cells				
**Gene name**	**Description**	**Log2FC**	**Style**	**FDR**
ATP6	ATP synthase F0 subunit 6	-1.53575668	down	1.45E-13
ATP8	ATP synthase F0 subunit 8	-1.6589645	down	1.18E-07
COX2	cytochrome c oxidase subunit II	-1.2370861	down	5.71E-17
COX3	cytochrome c oxidase III	-1.44826452	down	8.85E-11
CYTB	cytochrome b	-1.2697585	down	1.89E-05
ND2	MTND2	-1.00581934	down	0.000212
ND3	NADH dehydrogenase, subunit 3 (complex I)	-1.03133955	down	9.64E-05
ND4	NADH dehydrogenase, subunit 4 (complex I)	-0.64349502	down	1.84E-05
ND4L	NADH dehydrogenase, subunit 4L (complex I)	-0.60384849	down	0.008627
ND5	NADH dehydrogenase, subunit 5 (complex I)	-0.8903699	down	2.51E-06
ND6	NADH dehydrogenase, subunit 6 (complex I)	-2.259422	down	6E-08
MARC1	mitochondrial amidoxime reducing component 1	-1.14654068	down	0.000409
A1BG	alpha-1-B glycoprotein	0.648350145	up	0.044294
AATK	apoptosis-associated tyrosine kinase	1.600461797	up	2.04E-05
ABCA10	ATP-binding cassette, sub-family A (ABC1), member 10	1.749191767	up	0.033371
ABCB10	ATP-binding cassette, sub-family B (MDR/TAP), member 10	-0.63742374	down	0.046731
ABCB9	ATP-binding cassette, sub-family B (MDR/TAP), member 9	0.826594314	up	0.000159
ABCC10	ATP-binding cassette, sub-family C (CFTR/MRP), member 10	1.085516571	up	0.00376
ABCC5	ATP-binding cassette, sub-family C (CFTR/MRP), member 5	0.930222077	up	1.22E-07
ABCC8	ATP-binding cassette, sub-family C (CFTR/MRP), member 8	0.858001868	up	0.007508
ABCE1	ATP-binding cassette, sub-family E (OABP), member 1	-0.64280182	down	0.020844

ABCF2	ATP-binding cassette, sub-family F (GCN20), member 2	-0.61625998	down	0.000298
ABCG2	ATP-binding cassette, sub-family G (WHITE), member 2 (Junior blood group)	-0.72970165	down	8.31E-06
ABCG4	ATP-binding cassette, sub-family G (WHITE), member 4	0.75972158	up	0.024445
ABHD13	abhydrolase domain containing 13	-1.14037888	down	9.12E-06
ABHD17C	abhydrolase domain containing 17C	0.81162595	up	6.42E-06
ABHD2	abhydrolase domain containing 2	-1.25411381	down	1.16E-06
ABLIM3	actin binding LIM protein family, member 3	1.529646915	up	7.18E-19
ABTB1	ankyrin repeat and BTB (POZ) domain containing 1	0.769804915	up	4.34E-05
ABTB2	ankyrin repeat and BTB (POZ) domain containing 2	0.897173452	up	4.25E-07
ACAA2	acetyl-CoA acyltransferase 2	-0.65445369	down	0.000117
ACACA	acetyl-CoA carboxylase alpha	-0.84897401	down	1.51E-07
ACAD9	acyl-CoA dehydrogenase family, member 9	-0.71406085	down	0.000242
ACBD3	acyl-CoA binding domain containing 3	0.760028794	up	0.004104
ACBD7	acyl-CoA binding domain containing 7	-1.04870619	down	0.005721
ACHE	acetylcholinesterase (Yt blood group)	2.508314947	up	4.11E-19
ACOT13	acyl-CoA thioesterase 13	-0.6043867	down	0.000456
ACOT9	acyl-CoA thioesterase 9	-0.61850204	down	0.000972
ACOX3	acyl-CoA oxidase 3, pristanoyl	-0.87984416	down	0.000784
ACPP	acid phosphatase, prostate	-4.63255103	down	0.000529
ACSL3	acyl-CoA synthetase long-chain family member 3	-0.71535929	down	0.009378
ACSS1	acyl-CoA synthetase short-chain family member 1	-1.08510057	down	1.42E-09
ACSS2	acyl-CoA synthetase short-chain family member 2	0.694493446	up	3.83E-05

ACTL6A	actin-like 6A	-0.70590764	down	0.000164
ACVR1	activin A receptor, type I	0.63214224	up	0.000324
ACVRL1	activin A receptor type II-like 1	1.294725438	up	2.15E-05
ACYP1	acylphosphatase 1, erythrocyte (common) type	-0.72653927	down	0.004168
ADA	adenosine deaminase	-1.16129682	down	4.29E-09
ADAM10	ADAM metallopeptidase domain 10	-0.73579653	down	3.15E-06
ADAM12	ADAM metallopeptidase domain 12	1.259657817	up	9.46E-09
ADAM19	ADAM metallopeptidase domain 19	0.883413292	up	2.58E-07
ADAMTS15	ADAM metallopeptidase with thrombospondin type 1 motif, 15	0.782108812	up	0.00032
ADAMTS8	ADAM metallopeptidase with thrombospondin type 1 motif, 8	-2.59729822	down	0.02319
ADAP1	ArfGAP with dual PH domains 1	0.840921541	up	0.005018
ADAT1	adenosine deaminase, tRNA-specific 1	-0.72803987	down	0.000906
ADCK1	aarF domain containing kinase 1	-0.88333968	down	0.001676
ADCY3	adenylate cyclase 3	-0.84740137	down	5.08E-07
ADCY5	adenylate cyclase 5	0.988337365	up	0.022198
ADCY9	adenylate cyclase 9	-1.11096007	down	1.68E-11
ADGRE1	egf-like module containing, mucin-like, hormone receptor-like 1	1.140593533	up	7.21E-09
ADGRE5	CD97 molecule	0.798104093	up	1.47E-07
ADGRF4	G protein-coupled receptor 115	1.215902484	up	0.002806
ADGRG1	G protein-coupled receptor 56	1.17926008	up	2.83E-08
ADORA2A	adenosine A2a receptor	1.583774961	up	1.25E-06
ADPRHL2	ADP-ribosylhydrolase like 2	0.726445457	up	0.000128
ADPRM	ADP-ribose/CDP-alcohol diphosphatase, manganese-dependent	1.489443867	up	5.55E-0

				7
ADSSL1	adenylosuccinate synthase like 1	-0.71316922	down	0.001603
AGGF1	angiogenic factor with G patch and FHA domains 1	-0.89416277	down	8.45E-07
AGL	amylo-alpha-1, 6-glucosidase, 4-alpha-glucanotransferase	-0.95236425	down	0.002613
AGPAT1	1-acylglycerol-3-phosphate O-acyltransferase 1	-0.90295751	down	1.6E-07
AGR2	anterior gradient 2, protein disulphide isomerase family member	-0.83531072	down	2.61E-08
AHDC1	AT hook, DNA binding motif, containing 1	0.5990959	up	0.038014
AHSG	alpha-2-HS-glycoprotein	-2.20058307	down	0.018419
AJUBA	ajuba LIM protein	-1.62154476	down	3.43E-22
AK1	adenylate kinase 1	0.72053749	up	4.25E-05
AKAP2	A kinase (PRKA) anchor protein 2	1.061190395	up	1.34E-08
AKAP5	A kinase (PRKA) anchor protein 5	-1.43287812	down	0.015261
AKAP8L	A kinase (PRKA) anchor protein 8-like	0.669389624	up	9.13E-05
AKIRIN1	akirin 1	0.633588889	up	0.001592
AKR1B10	aldo-keto reductase family 1, member B10 (aldose reductase)	-0.96812773	down	8.58E-09
AKR1C1	aldo-keto reductase family 1, member C1	-0.65430503	down	0.019785
AKR1C3	aldo-keto reductase family 1, member C3	-0.83604122	down	1.71E-08
AKR7A2	aldo-keto reductase family 7, member A2 (aflatoxin aldehyde reductase)	-1.0025283	down	1.54E-05
ALDH16A1	aldehyde dehydrogenase 16 family, member A1	-0.81373823	down	0.001413
ALDH1A1	aldehyde dehydrogenase 1 family, member A1	-3.29529158	down	5.7E-109
ALDH1A3	aldehyde dehydrogenase 1 family, member A3	1.355408599	up	3.75E-16
ALDH3A1	aldehyde dehydrogenase 3 family, member A1	-1.37251829	down	3.23E-21
ALDH3A2	aldehyde dehydrogenase 3 family, member A2	-1.24616081	down	4.59E-1

				7
ALDH5A1	aldehyde dehydrogenase 5 family, member A1	-0.62286474	down	0.00903
ALDH7A1	aldehyde dehydrogenase 7 family, member A1	-0.69774356	down	3.62E-05
ALDOC	aldolase C, fructose-bisphosphate	1.357103403	up	8.73E-10
ALG10	ALG10, alpha-1,2-glucosyltransferase	-1.34108328	down	0.000921
ALG10B	ALG10B, alpha-1,2-glucosyltransferase	-0.84005745	down	0.004414
ALG14	ALG14, UDP-N-acetylglucosaminyltransferase subunit	-1.80056114	down	7.28E-06
ALG6	ALG6, alpha-1,3-glucosyltransferase	-1.32856827	down	5.54E-05
ALOX5	arachidonate 5-lipoxygenase	1.55125736	up	1.73E-10
ALOXE3	arachidonate lipoxygenase 3	1.994973784	up	1.52E-11
ALYREF	Aly/REF export factor	-0.8901296	down	2.07E-05
AMDHD2	amidohydrolase domain containing 2	0.849564307	up	0.000468
AMN1	antagonist of mitotic exit network 1 homolog (S. cerevisiae)	0.828916812	up	8.55E-06
AMOTL2	angiomotin like 2	-0.9210467	down	2.23E-08
AMPD3	adenosine monophosphate deaminase 3	1.342097572	up	1.24E-10
ANAPC1	anaphase promoting complex subunit 1	-1.27982842	down	0.000558
ANGPTL4	angiopoietin-like 4	0.855345078	up	0.045732
ANK3	ankyrin 3, node of Ranvier (ankyrin G)	-0.62878216	down	0.04694
ANKDD1A	ankyrin repeat and death domain containing 1A	0.852006169	up	0.005609
ANKFY1	ankyrin repeat and FYVE domain containing 1	-0.95335219	down	0.017402
ANKRD1	ankyrin repeat domain 1 (cardiac muscle)	-1.68938942	down	9.49E-06
ANKRD13D	ankyrin repeat domain 13 family, member D	0.798245186	up	2.02E-05
ANKRD2	ankyrin repeat domain 2 (stretch responsive muscle)	-1.27160731	down	0.010653

ANKRD24	ankyrin repeat domain 24	2.08017987	up	6.18E-16
ANKRD29	ankyrin repeat domain 29	0.972671848	up	4.68E-08
ANKRD33B	ankyrin repeat domain 33B	0.660575877	up	0.002495
ANKRD36B	ankyrin repeat domain 36B	-1.08892667	down	0.037302
ANKRD40	ankyrin repeat domain 40	-0.64318533	down	0.000207
ANKRD46	ankyrin repeat domain 46	0.637101237	up	0.015088
ANKS1A	ankyrin repeat and sterile alpha motif domain containing 1A	-1.22187952	down	2.98E-06
ANLN	anillin, actin binding protein	-1.44588074	down	1.53E-21
ANOS1	Kallmann syndrome 1 sequence	3.023443594	up	0.02546
ANP32E	acidic (leucine-rich) nuclear phosphoprotein 32 family, member E	-1.23054629	down	5.1E-14
ANPEP	alanyl (membrane) aminopeptidase	0.86915482	up	4.34E-05
ANTXR1	anthrax toxin receptor 1	-0.83062383	down	0.000731
ANTXR2	anthrax toxin receptor 2	1.622326743	up	4E-12
ANXA10	annexin A10	-1.84866588	down	0.033022
ANXA13	annexin A13	-1.01112774	down	0.001175
ANXA2R	annexin A2 receptor	1.33874919	up	1.56E-06
ANXA3	annexin A3	-0.70591648	down	6.92E-05
ANXA8	annexin A8	-0.92289267	down	0.000305
ANXA8L1	annexin A8-like 1	-0.96577184	down	4.53E-07
AOAH	acyloxyacyl hydrolase (neutrophil)	20	up	0.039329
AOC3	amine oxidase, copper containing 3	0.785086123	up	0.046308
AP1M2	adaptor-related protein complex 1, mu 2 subunit	-1.55668849	down	3.69E-11
AP1S1	adaptor-related protein complex 1, sigma 1 subunit	-1.2608073	down	3.74E-16

AP3S1	adaptor-related protein complex 3, sigma 1 subunit	0.604195607	up	0.002973
AP4S1	adaptor-related protein complex 4, sigma 1 subunit	-0.84700773	down	0.030411
AP5M1	adaptor-related protein complex 5, mu 1 subunit	-0.84679325	down	3E-05
AP5Z1	adaptor-related protein complex 5, zeta 1 subunit	1.287993454	up	2.07E-06
APH1B	APH1B gamma secretase subunit	-1.10539948	down	1.23E-09
API5	apoptosis inhibitor 5	-0.60166284	down	0.000405
APITD1-CORT	APITD1-CORT readthrough	-0.96298239	down	2.19E-05
APOBEC3B	apolipoprotein B mRNA editing enzyme, catalytic polypeptide-like 3B	-0.79813894	down	0.000131
APOE	apolipoprotein E	0.931969437	up	0.008126
APOH	apolipoprotein H (beta-2-glycoprotein I)	-1.30477139	down	3.71E-06
APOL1	apolipoprotein L, 1	0.818364737	up	1.21E-05
APOL3	apolipoprotein L, 3	1.518594958	up	0.001964
APPL2	adaptor protein, phosphotyrosine interaction, PH domain and leucine zipper containing 2	-1.5545697	down	1.44E-16
AQP11	aquaporin 11	0.887337905	up	0.024565
AR	androgen receptor	-1.17666448	down	1.47E-08
ARC	activity-regulated cytoskeleton-associated protein	1.831321663	up	0.004439
AREG	amphiregulin	1.867935512	up	7.47E-32
ARFGAP1	ADP-ribosylation factor GTPase activating protein 1	0.753539953	up	4.49E-05
ARFRP1	ADP-ribosylation factor related protein 1	0.706262951	up	0.000301
ARG2	arginase 2	1.681895852	up	8.97E-06
ARHGAP11A	Rho GTPase activating protein 11A	-1.69426873	down	4.88E-17
ARHGAP19	Rho GTPase activating protein 19	-0.63817771	down	0.002547

ARHGAP22	Rho GTPase activating protein 22	0.908758408	up	0.004901
ARHGAP26	Rho GTPase activating protein 26	0.951247367	up	0.037128
ARHGEF2	Rho/Rac guanine nucleotide exchange factor (GEF) 2	1.114740443	up	2.4E-10
ARHGEF3	Rho guanine nucleotide exchange factor (GEF) 3	0.934432704	up	1.37E-07
ARHGEF39	Rho guanine nucleotide exchange factor (GEF) 39	-1.01032533	down	4.69E-05
ARHGEF4	Rho guanine nucleotide exchange factor (GEF) 4	1.327182864	up	1.28E-10
ARID3A	AT rich interactive domain 3A (BRIGHT-like)	-0.6184333	down	0.002235
ARID3B	AT rich interactive domain 3B (BRIGHT-like)	0.675355322	up	0.027359
ARID5A	AT rich interactive domain 5A (MRF1-like)	0.844924752	up	0.002216
ARL13B	ADP-ribosylation factor-like 13B	-1.22957211	down	0.003456
ARL4C	ADP-ribosylation factor-like 4C	0.779555519	up	2.45E-07
ARL6	ADP-ribosylation factor-like 6	-0.758893	down	0.023141
ARL6IP1	ADP-ribosylation factor-like 6 interacting protein 1	-1.60958759	down	3.83E-27
ARMC1	armadillo repeat containing 1	-0.61262441	down	0.000833
ARMCX1	armadillo repeat containing, X-linked 1	3.562640546	up	7.57E-07
ARMCX6	armadillo repeat containing, X-linked 6	0.973988204	up	2.97E-05
ARPC4	actin related protein 2/3 complex, subunit 4, 20kDa	-1.21935309	down	4.65E-14
ARPC4-TTLL3	ARPC4-TTLL3 readthrough	-1.23405338	down	5.18E-12
ARRDC3	arrestin domain containing 3	1.386405851	up	3.75E-10
ARRDC4	arrestin domain containing 4	1.682547296	up	0.005636
ARSB	arylsulfatase B	-1.0940255	down	0.000965
ARSD	arylsulfatase D	-0.67525748	down	0.000329

ARSJ	arylsulfatase family, member J	-0.67010792	down	0.004383
ARVCF	armadillo repeat gene deleted in velocardiofacial syndrome	1.333824096	up	3.36E-06
ASB2	ankyrin repeat and SOCS box containing 2	2.311241112	up	4.37E-09
ASF1B	anti-silencing function 1B histone chaperone	-1.78133896	down	1.54E-19
ASH1L	ash1 (absent, small, or homeotic)-like (Drosophila)	-0.6793808	down	0.001954
ASIC3	acid-sensing (proton-gated) ion channel 3	0.628677705	up	0.019116
ASPM	asp (abnormal spindle) homolog, microcephaly associated (Drosophila)	-2.04520711	down	5.55E-10
ASRGL1	asparaginase like 1	-1.32988483	down	6.47E-05
ASS1	argininosuccinate synthase 1	1.025680412	up	2.5E-11
ASXL2	additional sex combs like transcriptional regulator 2	-0.83889765	down	0.004031
ATAD2	ATPase family, AAA domain containing 2	-1.40481556	down	9.72E-11
ATAD5	ATPase family, AAA domain containing 5	-1.37227865	down	0.000178
ATE1	arginyltransferase 1	-0.60281783	down	0.001338
ATF1	activating transcription factor 1	-0.81968867	down	0.000127
ATF3	activating transcription factor 3	2.015461209	up	0.002571
ATF7	activating transcription factor 7	0.619659716	up	0.00069
ATG14	autophagy related 14	0.659353125	up	0.0014
ATG2A	autophagy related 2A	1.084951146	up	5.64E-06
ATG4A	autophagy related 4A, cysteine peptidase	0.661125246	up	0.005838
ATG4C	autophagy related 4C, cysteine peptidase	-1.12854348	down	0.001026
ATG4D	autophagy related 4D, cysteine peptidase	0.631089656	up	0.035129
ATG9B	autophagy related 9B	1.840641605	up	0.007734
ATL3	atlastin GTPase 3	-0.81846818	down	3.72E-06

ATP11C	ATPase, class VI, type 11C	-1.29299894	down	3.11E-11
ATP1A3	ATPase, Na+/K+ transporting, alpha 3 polypeptide	0.944123906	up	0.00012
ATP2A3	ATPase, Ca++ transporting, ubiquitous	0.723356262	up	5.43E-06
ATP5F1	ATP synthase, H+ transporting, mitochondrial Fo complex, subunit B1	-1.04867843	down	5.23E-11
ATP5G1	ATP synthase, H+ transporting, mitochondrial Fo complex, subunit C1 (subunit 9)	-0.67071248	down	3.63E-05
ATP6V0A1	ATPase, H+ transporting, lysosomal V0 subunit a1	0.809830324	up	2.55E-06
ATP6V0A4	ATPase, H+ transporting, lysosomal V0 subunit a4	-1.97761762	down	7.45E-07
ATP6V0B	ATPase, H+ transporting, lysosomal 21kDa, V0 subunit b	0.672728427	up	1.75E-05
ATP6V0D2	ATPase, H+ transporting, lysosomal 38kDa, V0 subunit d2	3.885648386	up	0.028401
ATP6V1E1	ATPase, H+ transporting, lysosomal 31kDa, V1 subunit E1	-0.65118623	down	7.35E-05
ATP6V1E2	ATPase, H+ transporting, lysosomal 31kDa, V1 subunit E2	-1.31865393	down	2.51E-07
ATP9A	ATPase, class II, type 9A	-0.62072612	down	0.038573
ATRX	alpha thalassemia/mental retardation syndrome X-linked	-0.9706643	down	0.008686
ATXN7L1	ataxin 7-like 1	0.925261344	up	0.000149
AUNIP	aurora kinase A and ninein interacting protein	-0.98305599	down	0.018056
AURKA	aurora kinase A	-1.05428713	down	3.71E-11
AURKB	aurora kinase B	-0.59561156	down	0.000449
AXIN2	axin 2	-0.93368462	down	3.12E-06
B2M	beta-2-microglobulin	0.804047869	up	1.23E-07
B3GALT5	UDP-Gal:betaGlcNAc beta 1,3-galactosyltransferase, polypeptide 5	-1.28487721	down	0.030411
B4GALT1	UDP-Gal:betaGlcNAc beta 1,4- galactosyltransferase, polypeptide 1	-0.84849105	down	2.6E-08
B4GALT5	UDP-Gal:betaGlcNAc beta 1,4- galactosyltransferase, polypeptide 5	-0.87480028	down	1.07E-08
B4GALT6	UDP-Gal:betaGlcNAc beta 1,4- galactosyltransferase, polypeptide 6	-1.13800248	down	2.03E-0

				5
B4GALT7	xylosylprotein beta 1,4-galactosyltransferase, polypeptide 7	-2.10684722	down	1.04E-27
BACH1	BTB and CNC homology 1, basic leucine zipper transcription factor 1	0.684479204	up	0.003692
BAG1	BCL2-associated athanogene	0.739898972	up	1.14E-05
BAIAP2	BAI1-associated protein 2	0.806954508	up	1.2E-06
BAIAP3	BAI1-associated protein 3	1.960903292	up	1.58E-14
BAK1	BCL2-antagonist/killer 1	1.635113762	up	7.76E-21
BAMBI	BMP and activin membrane-bound inhibitor	-1.57843005	down	1.84E-19
BARD1	BRCA1 associated RING domain 1	-0.70632904	down	0.005043
BARX1	BARX homeobox 1	1.100337356	up	1.83E-06
BATF2	basic leucine zipper transcription factor, ATF-like 2	1.16041055	up	0.001069
BBC3	BCL2 binding component 3	1.253263668	up	0.000473
BBS4	Bardet-Biedl syndrome 4	0.67666882	up	8.99E-05
BCAR1	breast cancer anti-estrogen resistance 1	0.603042597	up	0.013074
BCAS1	breast carcinoma amplified sequence 1	-1.02648614	down	7.73E-06
BCAS2	breast carcinoma amplified sequence 2	-0.89178	down	0.011434
BCKDHB	branched chain keto acid dehydrogenase E1, beta polypeptide	-0.88162566	down	0.00017
BCL2A1	BCL2-related protein A1	1.397888581	up	0.011159
BCL2L15	BCL2-like 15	-1.04966592	down	0.003153
BCL6	B-cell CLL/lymphoma 6	0.964386043	up	0.000883
BCL7A	B-cell CLL/lymphoma 7A	0.82081491	up	3.78E-06
BCL9	B-cell CLL/lymphoma 9	0.871675384	up	3.99E-07
BCLAF1	BCL2-associated transcription factor 1	-1.29772331	down	1.07E-08

BDH2	3-hydroxybutyrate dehydrogenase, type 2	-0.96916217	down	0.000732
BDKRB1	bradykinin receptor B1	1.243681854	up	0.002243
BDKRB2	bradykinin receptor B2	1.074806054	up	0.004698
BEND3	BEN domain containing 3	-1.03495593	down	0.007551
BET1L	Bet1 golgi vesicular membrane trafficking protein-like	0.813408768	up	0.00014
BEX2	brain expressed X-linked 2	1.727767281	up	3.43E-06
BEX4	brain expressed, X-linked 4	2.236530285	up	1.96E-08
BHLHA15	basic helix-loop-helix family, member a15	3.19998636	up	0.006375
BIK	BCL2-interacting killer (apoptosis-inducing)	1.490446567	up	7.79E-12
BIRC3	baculoviral IAP repeat containing 3	1.246819719	up	0.016109
BIRC5	baculoviral IAP repeat containing 5	-1.36543738	down	1.75E-19
BLM	Bloom syndrome, RecQ helicase-like	-1.37089199	down	2.46E-08
BLMH	bleomycin hydrolase	-0.6130669	down	0.000668
BLOC1S6	biogenesis of lysosomal organelles complex-1, subunit 6, pallidin	-1.40061529	down	2.34E-08
BMF	Bcl2 modifying factor	2.240267737	up	2.29E-43
BMP2	bone morphogenetic protein 2	1.77270955	up	0.011471
BMP4	bone morphogenetic protein 4	-0.59587774	down	0.002838
BMPER	BMP binding endothelial regulator	1.034196341	up	2.4E-06
BNIP1	BCL2/adenovirus E1B 19kDa interacting protein 1	1.199200004	up	3.82E-09
BOLA1	bolA family member 1	-0.65329732	down	0.01105
BOLA3	bolA family member 3	-0.61470695	down	0.004252
BPTF	bromodomain PHD finger transcription factor	-0.82012885	down	5.59E-07
BRCA1	breast cancer 1, early onset	-1.15024382	down	2.17E-10

BRCA2	breast cancer 2, early onset	-1.77507638	down	0.009155
BRCC3	BRCA1/BRCA2-containing complex, subunit 3	-0.90545549	down	2.66E-05
BRF2	BRF2, RNA polymerase III transcription initiation factor 50 kDa subunit	1.00159613	up	2.71E-07
BRI3	brain protein I3	0.88979247	up	9.11E-09
BRI3BP	BRI3 binding protein	-1.91687611	down	1.47E-16
BRIP1	BRCA1 interacting protein C-terminal helicase 1	-1.6643182	down	7.05E-11
BRSK1	BR serine/threonine kinase 1	1.256409239	up	1.13E-06
BST2	bone marrow stromal cell antigen 2	1.103391948	up	0.002124
BTG1	B-cell translocation gene 1, anti-proliferative	0.807630544	up	0.000163
BTG2	BTG family, member 2	1.089619615	up	6.83E-12
BTN2A1	butyrophilin, subfamily 2, member A1	0.620679884	up	0.000521
BTN2A2	butyrophilin, subfamily 2, member A2	0.608429158	up	0.001496
BUB1	BUB1 mitotic checkpoint serine/threonine kinase	-0.75783689	down	3.21E-05
BUB1B	BUB1 mitotic checkpoint serine/threonine kinase B	-1.55067816	down	1.46E-18
C10orf10	chromosome 10 open reading frame 10	1.289379052	up	0.006599
C10orf91	chromosome 10 open reading frame 91	-0.9093672	down	0.0377
C11orf63	chromosome 11 open reading frame 63	0.841372363	up	0.016827
C11orf86	chromosome 11 open reading frame 86	1.112589239	up	1.73E-08
C11orf96	chromosome 11 open reading frame 96	2.168647927	up	0.033698
C12orf4	chromosome 12 open reading frame 4	-0.64883562	down	0.035198
C12orf76	chromosome 12 open reading frame 76	-0.63719578	down	0.028724
C14orf1	chromosome 14 open reading frame 1	-0.74560833	down	2.98E-05

C16orf45	chromosome 16 open reading frame 45	1.009937973	up	1.02E-06
C17orf100	chromosome 17 open reading frame 100	-0.80124315	down	0.024417
C17orf51	chromosome 17 open reading frame 51	-0.88414469	down	4.88E-05
C17orf97	chromosome 17 open reading frame 97	-1.02358271	down	0.015669
C18orf54	chromosome 18 open reading frame 54	-0.84441562	down	0.022396
C19orf66	chromosome 19 open reading frame 66	0.840489197	up	5.68E-07
C1GALT1	core 1 synthase, glycoprotein-N-acetylgalactosamine 3-beta-galactosyltransferase 1	-1.11697303	down	1.49E-09
C1GALT1C1	C1GALT1-specific chaperone 1	-0.59960812	down	0.042255
C1orf109	chromosome 1 open reading frame 109	-1.11540964	down	5.62E-07
C1orf112	chromosome 1 open reading frame 112	-0.78418618	down	0.00597
C1orf116	chromosome 1 open reading frame 116	-1.89709107	down	1.96E-13
C1orf52	chromosome 1 open reading frame 52	0.873272997	up	5.4E-05
C1QBP	complement component 1, q subcomponent binding protein	-0.65370928	down	3.22E-05
C1QL4	complement component 1, q subcomponent-like 4	0.631703835	up	0.006833
C1QTNF4	C1q and tumor necrosis factor related protein 4	2.280835915	up	0.045175
C1R	complement component 1, r subcomponent	0.759818965	up	1.16E-06
C20orf24	chromosome 20 open reading frame 24	-1.0557661	down	3.76E-11
C21orf58	chromosome 21 open reading frame 58	-1.82631726	down	3.59E-16
C22orf46	chromosome 22 open reading frame 46	-0.98700376	down	2.27E-05
C2CD2L	C2CD2-like	1.064374231	up	2.51E-06
C2orf68	chromosome 2 open reading frame 68	-0.75196806	down	5.25E-05
C2orf82	chromosome 2 open reading frame 82	-0.80295584	down	0.010375
C3	complement component 3	0.721222042	up	1.72E-0

				6
C3orf18	chromosome 3 open reading frame 18	-1.21154645	down	2.42E-07
C3orf52	chromosome 3 open reading frame 52	1.476625475	up	0.001417
C3orf58	chromosome 3 open reading frame 58	-1.35699248	down	4.57E-07
C4orf46	chromosome 4 open reading frame 46	-1.09973862	down	3.42E-06
C5	complement component 5	-1.41667992	down	7.86E-11
C5AR1	complement component 5a receptor 1	1.085223388	up	0.001128
C5orf30	chromosome 5 open reading frame 30	-0.69055964	down	0.000914
C5orf34	chromosome 5 open reading frame 34	-1.41265182	down	0.025864
C6orf1	chromosome 6 open reading frame 1	1.124790615	up	3.04E-06
C6orf141	chromosome 6 open reading frame 141	0.784621419	up	0.000177
C6orf223	chromosome 6 open reading frame 223	-1.18559822	down	0.042721
C6orf48	chromosome 6 open reading frame 48	1.747802792	up	5.38E-20
C6orf99	chromosome 6 open reading frame 99	-1.25527394	down	0.008054
C7orf43	chromosome 7 open reading frame 43	0.789643986	up	0.002293
C7orf55-LUC7L2	C7orf55-LUC7L2 readthrough	-0.70014343	down	2.49E-05
C8orf58	chromosome 8 open reading frame 58	-0.80414775	down	0.000777
C9orf142	chromosome 9 open reading frame 142	0.607432631	up	0.012313
C9orf163	chromosome 9 open reading frame 163	-1.3178839	down	0.01392
C9orf64	chromosome 9 open reading frame 64	-0.7569443	down	9.73E-05
C9orf85	chromosome 9 open reading frame 85	0.84600235	up	0.000131
CA2	carbonic anhydrase II	0.922598865	up	0.013091
CA4	carbonic anhydrase IV	2.31161517	up	0.00112

				8
CA9	carbonic anhydrase IX	1.853455077	up	5.71E-17
CACFD1	calcium channel flower domain containing 1	-0.82627074	down	0.000258
CACNB1	calcium channel, voltage-dependent, beta 1 subunit	0.619846911	up	0.015715
CACNB3	calcium channel, voltage-dependent, beta 3 subunit	0.911577661	up	8.83E-07
CACNG6	calcium channel, voltage-dependent, gamma subunit 6	-1.01538049	down	7.17E-08
CACNG7	calcium channel, voltage-dependent, gamma subunit 7	0.937700467	up	2.5E-08
CALB2	calbindin 2	1.359398024	up	1.91E-08
CALCOCO1	calcium binding and coiled-coil domain 1	0.653719806	up	6.79E-05
CALM1	calmodulin 1 (phosphorylase kinase, delta)	-1.04234326	down	7.18E-12
CALM3	calmodulin 3 (phosphorylase kinase, delta)	-0.97530164	down	7.2E-11
CALU	calumenin	-1.46460348	down	3.35E-20
CALY	calcyon neuron-specific vesicular protein	2.497123793	up	9.85E-12
CAMLG	calcium modulating ligand	0.74893084	up	6E-06
CAMSAP3	calmodulin regulated spectrin-associated protein family, member 3	0.74631133	up	0.012143
CAP2	CAP, adenylate cyclase-associated protein, 2 (yeast)	-1.15741827	down	0.000311
CAPN12	calpain 12	0.852046608	up	6.79E-05
CARD11	caspase recruitment domain family, member 11	1.522043048	up	0.040094
CARNMT1	chromosome 9 open reading frame 41	-1.05115408	down	0.000911
CASC10	cancer susceptibility candidate 10	-0.72770078	down	0.017962
CASKIN1	CASK interacting protein 1	0.885782773	up	0.00592
CASP7	caspase 7, apoptosis-related cysteine peptidase	0.603238844	up	0.000714
CASP8AP2	caspase 8 associated protein 2	-0.78251051	down	0.01781
CASP9	caspase 9, apoptosis-related cysteine peptidase	-0.63875292	down	0.002293
CASZ1	castor zinc finger 1	0.699024817	up	0.02624

				7
CATSPER1	cation channel, sperm associated 1	0.969036386	up	0.00014<br>4
CATSPERG	catsper channel auxiliary subunit gamma	1.115369206	up	0.00072<br>4
CAV1	caveolin 1, caveolae protein, 22kDa	-0.70579765	down	3.78E-0<br>6
CBFB	core-binding factor, beta subunit	-1.07066499	down	4.32E-0<br>8
CBX4	chromobox homolog 4	0.646351686	up	0.01598<br>2
CBX5	chromobox homolog 5	-2.41832496	down	1.51E-1<br>0
CCBE1	collagen and calcium binding EGF domains 1	1.367522994	up	0.00100<br>3
CCDC107	coiled-coil domain containing 107	0.776065165	up	2.39E-0<br>5
CCDC12	coiled-coil domain containing 12	-1.18446549	down	7.14E-1<br>0
CCDC174	coiled-coil domain containing 174	0.632700351	up	0.00347<br>8
CCDC18	coiled-coil domain containing 18	-2.60783294	down	0.03215<br>1
CCDC183	coiled-coil domain containing 183	0.634815071	up	0.02799<br>5
CCDC28B	coiled-coil domain containing 28B	1.528862791	up	4.53E-0<br>7
CCDC58	coiled-coil domain containing 58	-0.72235705	down	0.03471<br>2
CCDC69	coiled-coil domain containing 69	0.752808804	up	0.00137<br>5
CCDC80	coiled-coil domain containing 80	-1.08447733	down	2.61E-1<br>1
CCDC88A	coiled-coil domain containing 88A	-1.25794863	down	1.36E-0<br>5
CCDC92	coiled-coil domain containing 92	-0.78721043	down	1.43E-0<br>5
CCK	cholecystokinin	1.240368061	up	0.04042<br>5
CCL20	chemokine (C-C motif) ligand 20	1.549025655	up	0.00195<br>9
CCL22	chemokine (C-C motif) ligand 22	4.331117957	up	3.39E-0<br>9

CCL26	chemokine (C-C motif) ligand 26	-1.18546973	down	0.01581
CCL3	chemokine (C-C motif) ligand 3	3.636553041	up	0.000291
CCL5	chemokine (C-C motif) ligand 5	1.003055271	up	0.000746
CCNA2	cyclin A2	-1.22286581	down	3.24E-09
CCNB1	cyclin B1	-1.09714474	down	2.88E-13
CCNB1IP1	cyclin B1 interacting protein 1, E3 ubiquitin protein ligase	1.205526801	up	5.49E-14
CCNB2	cyclin B2	-1.24609184	down	2.04E-14
CCND2	cyclin D2	5.293479958	up	4.25E-05
CCNE2	cyclin E2	-1.2769785	down	0.020051
CCNG1	cyclin G1	0.988127544	up	9.58E-07
CCNL1	cyclin L1	0.915494343	up	0.001121
CCPG1	cell cycle progression 1	-1.01052769	down	0.000453
CCT4	chaperonin containing TCP1, subunit 4 (delta)	-0.9317763	down	6.78E-10
CCT5	chaperonin containing TCP1, subunit 5 (epsilon)	-0.89701289	down	1.63E-09
CCT6A	chaperonin containing TCP1, subunit 6A (zeta 1)	-0.99580046	down	5.66E-06
CCT7	chaperonin containing TCP1, subunit 7 (eta)	-0.6497463	down	2.8E-05
CD22	CD22 molecule	1.736900635	up	1.19E-17
CD2AP	CD2-associated protein	-0.73772315	down	0.009659
CD40	CD40 molecule, TNF receptor superfamily member 5	1.006677594	up	0.000158
CD55	CD55 molecule, decay accelerating factor for complement (Cromer blood group)	0.651129231	up	0.009437
CD5L	CD5 molecule-like	3.861217622	up	0.036293
CD68	CD68 molecule	1.551601567	up	4.9E-26
CD79B	CD79b molecule, immunoglobulin-associated beta	1.314413519	up	0.039647

CD82	CD82 molecule	1.123264004	up	1.9E-09
CD83	CD83 molecule	1.200554543	up	1.51E-06
CD99L2	CD99 molecule-like 2	-0.75781497	down	1.51E-06
CDA	cytidine deaminase	0.603964723	up	0.009547
CDADC1	cytidine and dCMP deaminase domain containing 1	0.769552817	up	0.017811
CDC20	cell division cycle 20	-0.76935748	down	1.26E-06
CDC25A	cell division cycle 25A	-1.0766722	down	0.024232
CDC25C	cell division cycle 25C	-0.85083898	down	7.05E-06
CDC34	cell division cycle 34	0.960409701	up	5.12E-07
CDC42BPA	CDC42 binding protein kinase alpha (DMPK-like)	-0.67533531	down	0.000136
CDC45	cell division cycle 45	-1.48818025	down	1.4E-12
CDC6	cell division cycle 6	-1.58300562	down	3.09E-21
CDC7	cell division cycle 7	-1.03572269	down	5.27E-05
CDC73	cell division cycle 73	-0.8184822	down	0.002994
CDCA3	cell division cycle associated 3	-2.14314473	down	7.58E-35
CDCA4	cell division cycle associated 4	-0.96090759	down	8.37E-09
CDCA5	cell division cycle associated 5	-0.6483704	down	0.000152
CDCA7	cell division cycle associated 7	-1.07669001	down	1.27E-10
CDCA7L	cell division cycle associated 7-like	-0.61272953	down	0.000431
CDCA8	cell division cycle associated 8	-1.64710471	down	5.4E-22
CDCP1	CUB domain containing protein 1	0.821399818	up	9.1E-08
CDH1	cadherin 1, type 1, E-cadherin (epithelial)	-0.70289294	down	0.006194
CDH2	cadherin 2, type 1, N-cadherin (neuronal)	-1.39042684	down	1.05E-16
CDHR1	cadherin-related family member 1	2.59839091	up	0.04424

				5
CDHR4	cadherin-related family member 4	2.277151639	up	0.00567<br>5
CDIP1	cell death-inducing p53 target 1	1.089865856	up	1.02E-0<br>6
CDK1	cyclin-dependent kinase 1	-1.00457853	down	0.00088<br>1
CDK13	cyclin-dependent kinase 13	-0.99201091	down	1.39E-0<br>7
CDK18	cyclin-dependent kinase 18	0.729694894	up	0.00027
CDK19	cyclin-dependent kinase 19	-1.80109584	down	7.15E-1<br>5
CDK2	cyclin-dependent kinase 2	-1.33082672	down	2.18E-1<br>5
CDK20	cyclin-dependent kinase 20	1.019345293	up	5.61E-0<br>6
CDK5RAP2	CDK5 regulatory subunit associated protein 2	-0.61988892	down	0.00015<br>5
CDK6	cyclin-dependent kinase 6	-0.90274202	down	2.19E-0<br>6
CDKN1A	cyclin-dependent kinase inhibitor 1A (p21, Cip1)	0.6638044	up	0.00434<br>6
CDKN1B	cyclin-dependent kinase inhibitor 1B (p27, Kip1)	0.746439399	up	1.28E-0<br>5
CDKN1C	cyclin-dependent kinase inhibitor 1C (p57, Kip2)	1.044074376	up	0.00623<br>3
CDKN2AIP	CDKN2A interacting protein	0.688600896	up	0.01599<br>1
CDKN2C	cyclin-dependent kinase inhibitor 2C (p18, inhibits CDK4)	-0.61614715	down	0.00734<br>6
CDKN2D	cyclin-dependent kinase inhibitor 2D (p19, inhibits CDK4)	0.869128771	up	0.01630<br>7
CDS1	CDP-diacylglycerol synthase (phosphatidate cytidylyltransferase) 1	0.82055618	up	0.00438<br>3
CEACAM1	carcinoembryonic antigen-related cell adhesion molecule 1 (biliary glycoprotein)	0.997775157	up	1.58E-0<br>6
CELSR2	cadherin, EGF LAG seven-pass G-type receptor 2	-0.78520532	down	0.01372
CEND1	cell cycle exit and neuronal differentiation 1	4.158089384	up	2.84E-0<br>8
CENPA	centromere protein A	-1.17396591	down	4.07E-1<br>0
CENPBD1	CENPB DNA-binding domains containing 1	-0.7736487	down	0.04031<br>1

CENPE	centromere protein E, 312kDa	-2.17355472	down	1.66E-07
CENPH	centromere protein H	-0.80367443	down	0.030096
CENPI	centromere protein I	-1.34255976	down	2.19E-06
CENPJ	centromere protein J	-1.08048032	down	7.19E-07
CENPK	centromere protein K	-1.71698326	down	4.22E-08
CENPM	centromere protein M	-1.04755504	down	9.92E-07
CENPN	centromere protein N	-1.07086227	down	9.01E-09
CENPO	centromere protein O	-1.0669344	down	6.23E-10
CENPU	centromere protein U	-1.60196712	down	0.000969
CENPV	centromere protein V	-0.60446165	down	0.000274
CENPW	centromere protein W	-0.92049179	down	0.021339
CEP128	centrosomal protein 128kDa	-1.22670769	down	0.000553
CEP162	centrosomal protein 162kDa	-1.20127561	down	0.044277
CEP250	centrosomal protein 250kDa	-0.65869947	down	0.002031
CEP41	centrosomal protein 41kDa	-1.1607639	down	8.86E-09
CEP44	centrosomal protein 44kDa	-0.74415025	down	0.003581
CEP55	centrosomal protein 55kDa	-0.74866828	down	7.24E-05
CEP57L1	centrosomal protein 57kDa-like 1	-0.8780435	down	0.004956
CEP72	centrosomal protein 72kDa	0.705328773	up	0.000225
CEP76	centrosomal protein 76kDa	-0.60955625	down	0.014978
CEP78	centrosomal protein 78kDa	-0.68026903	down	0.000843
CEP95	centrosomal protein 95kDa	0.917107738	up	2.13E-05

CEP97	centrosomal protein 97kDa	-0.74989959	down	0.010239
CERS4	ceramide synthase 4	0.928420758	up	0.029775
CETN3	centrin, EF-hand protein, 3	-0.59231213	down	0.021517
CFB	complement factor B	1.048953912	up	1.41E-11
CFD	complement factor D (adipsin)	0.679447465	up	0.041973
CGN	cingulin	0.785927467	up	7.22E-05
CH17-360D5.1	neuropeptide Y receptor type 4-like	-1.00983165	down	1.32E-06
CHAC2	ChaC, cation transport regulator homolog 2 (E. coli)	-1.69495435	down	0.000406
CHAF1A	chromatin assembly factor 1, subunit A (p150)	-1.38303724	down	2.74E-09
CHAF1B	chromatin assembly factor 1, subunit B (p60)	-1.31232867	down	2.52E-05
CHAMP1	chromosome alignment maintaining phosphoprotein 1	-0.66359996	down	0.00165
CHCHD1	coiled-coil-helix-coiled-coil-helix domain containing 1	-0.71286705	down	0.010408
CHD5	chromodomain helicase DNA binding protein 5	1.602103381	up	1.79E-06
CHD7	chromodomain helicase DNA binding protein 7	0.660528691	up	0.000503
CHGB	chromogranin B (secretogranin 1)	1.112178582	up	5.29E-08
CHIC1	cysteine-rich hydrophobic domain 1	0.621894489	up	0.041625
CHORDC1	cysteine and histidine-rich domain (CHORD) containing 1	-0.74401409	down	0.000143
CHRM4	cholinergic receptor, muscarinic 4	2.37445106	up	6.3E-11
CHRNB1	cholinergic receptor, nicotinic, beta 1 (muscle)	0.821711786	up	0.000376
CHST1	carbohydrate (keratan sulfate Gal-6) sulfotransferase 1	1.025963825	up	0.00036
CHST14	carbohydrate (N-acetylgalactosamine 4-0) sulfotransferase 14	-1.23965549	down	2.69E-10
CHST15	carbohydrate (N-acetylgalactosamine 4-sulfate 6-O) sulfotransferase 15	-1.20214855	down	9.85E-12
CHST2	carbohydrate (N-acetylglucosamine-6-O) sulfotransferase 2	2.405469992	up	3.62E-12

CHTF8	CTF8, chromosome transmission fidelity factor 8 homolog (S. cerevisiae)	-0.59982378	down	0.000327
CIB1	calcium and integrin binding 1 (calmyrin)	1.19753166	up	3.74E-16
CIB2	calcium and integrin binding family member 2	0.780074579	up	5.54E-06
CIR1	corepressor interacting with RBPJ, 1	0.687107846	up	0.001045
CISD2	CDGSH iron sulfur domain 2	-1.57205797	down	4.05E-15
CIT	citron rho-interacting serine/threonine kinase	-0.59861208	down	0.001128
CKS1B	CDC28 protein kinase regulatory subunit 1B	-1.42644677	down	2.98E-08
CKS2	CDC28 protein kinase regulatory subunit 2	-0.59647973	down	0.0026
CLASP1	cytoplasmic linker associated protein 1	-0.64936928	down	0.000324
CLDN12	claudin 12	-0.81842979	down	6.76E-06
CLDN2	claudin 2	-2.32174728	down	1.61E-08
CLDN7	claudin 7	-1.02387371	down	0.018787
CLDND1	claudin domain containing 1	0.589748348	up	0.001013
CLDND2	claudin domain containing 2	-1.10839678	down	0.002822
CLEC2B	C-type lectin domain family 2, member B	1.466885618	up	0.015962
CLIC3	chloride intracellular channel 3	1.027364097	up	0.003873
CLIP1	CAP-GLY domain containing linker protein 1	-0.62500742	down	0.000567
CLIP2	CAP-GLY domain containing linker protein 2	0.74303831	up	0.000432
CLOCK	clock circadian regulator	-1.40872458	down	1.38E-07
CLSPN	claspin	-2.03558969	down	4.78E-19
CLSTN3	calsyntenin 3	-0.67668471	down	0.000244
CMTM4	CKLF-like MARVEL transmembrane domain containing 4	-0.80403653	down	0.000134

CMTM6	CKLF-like MARVEL transmembrane domain containing 6	-1.72689303	down	4.95E-26
CMTR2	cap methyltransferase 2	-0.58766638	down	0.013496
CNIH1	cornichon family AMPA receptor auxiliary protein 1	-1.28762226	down	8.58E-15
CNPPD1	cyclin Pas1/PHO80 domain containing 1	0.714246664	up	5.83E-05
CNTFR	ciliary neurotrophic factor receptor	2.622130381	up	0.023189
CNTN1	contactin 1	-0.74828181	down	0.040659
CNTNAP1	contactin associated protein 1	0.653850793	up	0.000154
CNTRL	centriolin	-0.71453633	down	6.84E-05
COG1	component of oligomeric golgi complex 1	0.826259993	up	8.82E-07
COL13A1	collagen, type XIII, alpha 1	2.184680366	up	0.005839
COL1A1	collagen, type I, alpha 1	1.009568309	up	1.8E-08
COL21A1	collagen, type XXI, alpha 1	0.749184566	up	0.003065
COL4A1	collagen, type IV, alpha 1	0.732334214	up	1.61E-06
COL5A3	collagen, type V, alpha 3	2.754438289	up	0.011995
COL6A1	collagen, type VI, alpha 1	0.737576118	up	0.000114
COL6A2	collagen, type VI, alpha 2	1.01604465	up	2.8E-07
COL6A3	collagen, type VI, alpha 3	1.158517713	up	0.037448
COL7A1	collagen, type VII, alpha 1	0.837345353	up	3.36E-06
COLEC12	collectin sub-family member 12	20	up	0.031911
COMMD10	COMM domain containing 10	-0.6318824	down	0.009098
COMMD4	COMM domain containing 4	-0.66859836	down	0.001391
COMMD7	COMM domain containing 7	-0.94680213	down	2.34E-08
COMP	cartilage oligomeric matrix protein	20	up	0.02257

				2
COQ10B	coenzyme Q10 homolog B (S. cerevisiae)	-0.82109371	down	1.2E-05
COQ3	coenzyme Q3 methyltransferase	-1.25416053	down	2.37E-06
COQ7	coenzyme Q7 homolog, ubiquinone (yeast)	-0.75705245	down	0.00075
CORO2A	coronin, actin binding protein, 2A	-1.76402289	down	4.52E-25
CORO2B	coronin, actin binding protein, 2B	1.919412726	up	0.000161
CORO6	coronin 6	0.862785696	up	0.010122
CORO7	coronin 7	0.84285969	up	0.000401
COX19	cytochrome c oxidase assembly homolog 19 (S. cerevisiae)	0.669837681	up	0.002594
COX7A2L	cytochrome c oxidase subunit VIIa polypeptide 2 like	0.615131209	up	0.000101
CPA4	carboxypeptidase A4	2.471074117	up	5.02E-24
CPEB4	cytoplasmic polyadenylation element binding protein 4	0.781242408	up	0.024594
CPM	carboxypeptidase M	1.202293408	up	9.15E-06
CPOX	coproporphyrinogen oxidase	-0.75547481	down	6.62E-05
CPSF3	cleavage and polyadenylation specific factor 3, 73kDa	-0.59018441	down	0.000883
CPSF6	cleavage and polyadenylation specific factor 6, 68kDa	-1.09312488	down	3.92E-11
CRABP2	cellular retinoic acid binding protein 2	1.417367867	up	1.83E-12
CREB3L1	cAMP responsive element binding protein 3-like 1	0.793997503	up	0.008134
CREB3L3	cAMP responsive element binding protein 3-like 3	1.342191072	up	0.000418
CREB5	cAMP responsive element binding protein 5	3.29821078	up	0.01532
CREBRF	CREB3 regulatory factor	1.728903805	up	0.001
CRELD1	cysteine-rich with EGF-like domains 1	0.628338049	up	0.001496
CRHR1	corticotropin releasing hormone receptor 1	1.685540526	up	0.000263
CRISPLD2	cysteine-rich secretory protein LCCL domain containing 2	1.603962167	up	1.12E-05

CRLF1	cytokine receptor-like factor 1	1.193543391	up	1.72E-05
CRMP1	collapsin response mediator protein 1	1.155715906	up	2.92E-07
CRNDE	colorectal neoplasia differentially expressed (non-protein coding)	-1.0326638	down	0.003839
CRTAC1	cartilage acidic protein 1	2.901550337	up	3.23E-21
CRTC2	CREB regulated transcription coactivator 2	0.679784634	up	7.98E-05
CRY2	cryptochrome circadian clock 2	1.272305916	up	3.77E-07
CRYM	crystallin, mu	1.102480251	up	9.52E-05
CSE1L	CSE1 chromosome segregation 1-like (yeast)	-0.69391946	down	8.06E-06
CSF2	colony stimulating factor 2 (granulocyte-macrophage)	1.703249299	up	0.008668
CSGALNACT1	chondroitin sulfate N-acetylgalactosaminyltransferase 1	-0.66942176	down	3.72E-05
CSGALNACT2	chondroitin sulfate N-acetylgalactosaminyltransferase 2	0.832024352	up	1.68E-06
CSPG5	chondroitin sulfate proteoglycan 5 (neuroglycan C)	-0.67538063	down	0.042005
CSRNP1	cysteine-serine-rich nuclear protein 1	1.450526968	up	8.24E-06
CSTF2	cleavage stimulation factor, 3' pre-RNA, subunit 2, 64kDa	-0.73209997	down	0.0004
CTDSP1	CTD (carboxy-terminal domain, RNA polymerase II, polypeptide A) small phosphatase 1	-1.50233186	down	1.53E-06
CTGF	connective tissue growth factor	-2.02134681	down	7.23E-12
CTNNBIP1	catenin, beta interacting protein 1	-0.61648249	down	0.004767
CTPS1	CTP synthase 1	-1.01570426	down	2.01E-10
CTSD	cathepsin D	0.680120892	up	9.43E-05
CTSH	cathepsin H	0.802513224	up	3.74E-06
CTSV	cathepsin V	-0.67482845	down	0.000351
CTTNBP2NL	CTTNBP2 N-terminal like	-1.23870754	down	2.92E-07

CUL1	cullin 1	-0.68193366	down	0.00126 1
CUL7	cullin 7	-0.66488028	down	0.00017 7
CXCL1	chemokine (C-X-C motif) ligand 1 (melanoma growth stimulating activity, alpha)	0.835197916	up	0.03964 7
CXCL8	chemokine (C-X-C motif) ligand 8	1.599347415	up	0.00169 4
CXorf38	chromosome X open reading frame 38	0.864624816	up	6.57E-0 7
CYB561D1	cytochrome b561 family, member D1	-0.83671276	down	0.00516 7
CYB5R1	cytochrome b5 reductase 1	1.086401584	up	4.28E-1 1
CYP1A1	cytochrome P450, family 1, subfamily A, polypeptide 1	1.779314657	up	4.37E-0 6
CYP1B1	cytochrome P450, family 1, subfamily B, polypeptide 1	1.47797441	up	2.23E-0 8
CYP27A1	cytochrome P450, family 27, subfamily A, polypeptide 1	2.806202102	up	0.00857 4
CYP27B1	cytochrome P450, family 27, subfamily B, polypeptide 1	1.426579511	up	0.00252 8
CYP2J2	cytochrome P450, family 2, subfamily J, polypeptide 2	2.233504968	up	0.00171 5
CYP2S1	cytochrome P450, family 2, subfamily S, polypeptide 1	0.79104308	up	5.06E-0 5
CYP4F11	cytochrome P450, family 4, subfamily F, polypeptide 11	-1.06510695	down	1.57E-1 1
CYP4F3	cytochrome P450, family 4, subfamily F, polypeptide 3	-0.7302561	down	0.00061 5
CYSTM1	cysteine-rich transmembrane module containing 1	-0.83043958	down	2.51E-0 7
CYTH1	cytohesin 1	0.664522028	up	0.00012 9
CYTH3	cytohesin 3	-1.3922191	down	5.22E-1 8
CYTH4	cytohesin 4	3.335321205	up	1.57E-1 0
DAAM1	dishevelled associated activator of morphogenesis 1	1.02392037	up	4.92E-0 7
DAB2	Dab, mitogen-responsive phosphoprotein, homolog 2 (Drosophila)	-0.75028672	down	0.00047 1
DACT3	dishevelled-binding antagonist of beta-catenin 3	2.536473371	up	7.4E-15

DAG1	dystroglycan 1 (dystrophin-associated glycoprotein 1)	-0.60523596	down	0.000214
DAGLA	diacylglycerol lipase, alpha	0.804205403	up	0.000132
DAGLB	diacylglycerol lipase, beta	0.903107596	up	5.39E-08
DAPK2	death-associated protein kinase 2	0.750416201	up	0.000329
DAPK3	death-associated protein kinase 3	0.912143845	up	2.64E-05
DARS2	aspartyl-tRNA synthetase 2, mitochondrial	-1.29712763	down	8.8E-16
DAZAP2	DAZ associated protein 2	-0.66133176	down	3.82E-05
DBF4B	DBF4 zinc finger B	-1.29573584	down	0.002048
DBI	diazepam binding inhibitor (GABA receptor modulator, acyl-CoA binding protein)	-0.88179266	down	6.63E-06
DBR1	debranching RNA lariats 1	-0.68422256	down	0.012032
DBT	dihydrolipoamide branched chain transacylase E2	-0.75596141	down	0.004006
DCAF13	DDB1 and CUL4 associated factor 13	-0.92953216	down	0.000291
DCAF6	DDB1 and CUL4 associated factor 6	-0.64485027	down	0.026509
DCBLD1	discoidin, CUB and LCCL domain containing 1	0.991924782	up	5.25E-10
DCDC2	doublecortin domain containing 2	-0.94922579	down	6.09E-07
DCHS1	dachsous cadherin-related 1	1.913328584	up	0.03369
DCK	deoxycytidine kinase	-0.9009831	down	0.000873
DCLK1	doublecortin-like kinase 1	1.869920393	up	1.13E-15
DCLRE1A	DNA cross-link repair 1A	-0.77276208	down	0.00085
DCLRE1B	DNA cross-link repair 1B	-0.59371923	down	0.008014
DCP1B	decapping mRNA 1B	0.809008228	up	3.35E-05
DCPS	decapping enzyme, scavenger	-0.61788466	down	0.002449
DCTN4	dynactin 4 (p62)	0.674623785	up	0.000115

DCUN1D3	DCN1, defective in cullin neddylation 1, domain containing 3	0.947279166	up	0.000169
DDAH1	dimethylarginine dimethylaminohydrolase 1	-1.39160517	down	4.22E-09
DDHD2	DDHD domain containing 2	-0.62231854	down	0.00054
DDIAS	DNA damage-induced apoptosis suppressor	-1.59899718	down	6.26E-10
DDIT3	DNA-damage-inducible transcript 3	2.496306932	up	1.39E-05
DDN	dendrin	1.651708117	up	0.021217
DDX24	DEAD (Asp-Glu-Ala-Asp) box helicase 24	0.641078994	up	4.38E-05
DDX39A	DEAD (Asp-Glu-Ala-Asp) box polypeptide 39A	-0.59422035	down	0.004398
DDX46	DEAD (Asp-Glu-Ala-Asp) box polypeptide 46	-0.96012078	down	8.17E-08
DDX58	DEAD (Asp-Glu-Ala-Asp) box polypeptide 58	0.690110733	up	0.036686
DEDD2	death effector domain containing 2	0.75354432	up	0.022106
DEK	DEK proto-oncogene	-0.96972037	down	9.28E-05
DENND1C	DENN/MADD domain containing 1C	0.795452386	up	0.004013
DENND2A	DENN/MADD domain containing 2A	1.282018668	up	0.002351
DENND2C	DENN/MADD domain containing 2C	1.500645036	up	0.005168
DENND3	DENN/MADD domain containing 3	0.878202506	up	9.39E-05
DENND6B	DENN/MADD domain containing 6B	0.650804478	up	0.029011
DEPDC1	DEP domain containing 1	-1.74036518	down	4.38E-08
DEPDC1B	DEP domain containing 1B	-1.43014861	down	1.14E-08
DERA	deoxyribose-phosphate aldolase (putative)	-0.83164347	down	0.001006
DES	desmin	1.667533086	up	0.027358
DEXI	Dexi homolog (mouse)	-2.48307021	down	1.04E-30

DFFB	DNA fragmentation factor, 40kDa, beta polypeptide (caspase-activated DNase)	-0.99048264	down	0.004961
DGAT2	diacylglycerol O-acyltransferase 2	0.891579255	up	0.001887
DGCR6	DiGeorge syndrome critical region gene 6	1.311923065	up	6.24E-09
DGKD	diacylglycerol kinase, delta 130kDa	0.623783631	up	6.18E-05
DHCR24	24-dehydrocholesterol reductase	-0.65629902	down	9.45E-05
DHDDS	dehydrodolichyl diphosphate synthase subunit	0.601682713	up	0.041372
DHDH	dihydrodiol dehydrogenase (dimeric)	1.26610205	up	0.010301
DHFR	dihydrofolate reductase	-1.99872471	down	4.92E-11
DHODH	dihydroorotate dehydrogenase (quinone)	-0.89785029	down	5.53E-05
DHRS2	dehydrogenase/reductase (SDR family) member 2	2.106004734	up	4.46E-23
DHRS4	dehydrogenase/reductase (SDR family) member 4	-0.6003025	down	0.004061
DHRS9	dehydrogenase/reductase (SDR family) member 9	1.768972606	up	2.06E-09
DHX15	DEAH (Asp-Glu-Ala-His) box helicase 15	-0.59575418	down	0.000388
DHX29	DEAH (Asp-Glu-Ala-His) box polypeptide 29	-1.06477875	down	2.34E-09
DHX33	DEAH (Asp-Glu-Ala-His) box polypeptide 33	-1.26723118	down	2.7E-14
DHX9	DEAH (Asp-Glu-Ala-His) box helicase 9	-0.86665365	down	8.61E-06
DIAPH3	diaphanous-related formin 3	-1.06342077	down	3.27E-06
DIRAS1	DIRAS family, GTP-binding RAS-like 1	0.873255316	up	7.22E-05
DISP2	dispatched homolog 2 (Drosophila)	1.376764189	up	0.00159
DKK1	dickkopf WNT signaling pathway inhibitor 1	-1.03125381	down	0.000288
DLD	dihydrolipoamide dehydrogenase	-0.70546055	down	0.000107
DLG1	discs, large homolog 1 (Drosophila)	-0.69731195	down	0.000491
DLG4	discs, large homolog 4 (Drosophila)	1.357171918	up	1.19E-1

				7
DLGAP4	discs, large (Drosophila) homolog-associated protein 4	0.802550742	up	0.000278
DLGAP5	discs, large (Drosophila) homolog-associated protein 5	-1.48559891	down	3.37E-15
DLL4	delta-like 4 (Drosophila)	1.181820597	up	0.010979
DMBX1	diencephalon/mesencephalon homeobox 1	-1.10952142	down	0.000349
DMKN	dermokine	1.153227497	up	1.26E-07
DMRTA1	DMRT-like family A1	1.432062265	up	4.33E-05
DMWD	dystrophia myotonica, WD repeat containing	0.694315923	up	0.000226
DNA2	DNA replication helicase/nuclease 2	-0.84172809	down	0.006306
DNAAF3	dynein, axonemal, assembly factor 3	0.634951323	up	0.003705
DNAJB2	DnaJ (Hsp40) homolog, subfamily B, member 2	0.833732368	up	2.84E-05
DNAJB5	DnaJ (Hsp40) homolog, subfamily B, member 5	1.188905582	up	4.2E-10
DNAJB9	DnaJ (Hsp40) homolog, subfamily B, member 9	1.160397765	up	6.41E-06
DNAJC1	DnaJ (Hsp40) homolog, subfamily C, member 1	0.65677428	up	0.000855
DNAJC13	DnaJ (Hsp40) homolog, subfamily C, member 13	-0.66012621	down	0.0004
DNAJC16	DnaJ (Hsp40) homolog, subfamily C, member 16	-0.64821676	down	0.008977
DNAJC22	DnaJ (Hsp40) homolog, subfamily C, member 22	-0.67379502	down	0.000121
DNAJC25	DnaJ (Hsp40) homolog, subfamily C , member 25	0.789669936	up	0.007324
DNAJC9	DnaJ (Hsp40) homolog, subfamily C, member 9	-0.86093685	down	7.6E-06
DNASE1	deoxyribonuclease I	-0.65565828	down	0.012799
DNMBP	dynamin binding protein	0.818356543	up	2.26E-07
DNMT1	DNA (cytosine-5-)-methyltransferase 1	-0.90953764	down	1.74E-08
DOC2A	double C2-like domains, alpha	0.8834906	up	0.00436
DOCK10	dedicator of cytokinesis 10	-0.77600097	down	4.05E-06

DOK5	docking protein 5	3.046477915	up	0.02773
DPF2	D4, zinc and double PHD fingers family 2	0.699840157	up	2.54E-05
DPH1	diphthamide biosynthesis 1	0.626903446	up	0.002064
DPP4	dipeptidyl-peptidase 4	1.608079288	up	0.00069
DPP8	dipeptidyl-peptidase 8	-0.71046919	down	0.000112
DPY19L3	dpy-19-like 3 (C. elegans)	-0.70764493	down	0.00248
DPY19L4	dpy-19-like 4 (C. elegans)	-1.31394333	down	2.61E-10
DPYSL4	dihydropyrimidinase-like 4	0.701997436	up	0.004024
DRAXIN	dorsal inhibitory axon guidance protein	2.379278682	up	0.000728
DROSHA	drosha, ribonuclease type III	-0.80146707	down	1.8E-06
DSC2	desmocollin 2	0.770046656	up	0.010506
DSC3	desmocollin 3	-0.90299962	down	0.045072
DSCAML1	Down syndrome cell adhesion molecule like 1	1.080003357	up	0.000302
DSCC1	DNA replication and sister chromatid cohesion 1	-2.45429542	down	5.23E-11
DSN1	DSN1, MIS12 kinetochore complex component	-0.72388797	down	0.000212
DTL	denticleless E3 ubiquitin protein ligase homolog (Drosophila)	-1.14996741	down	0.000169
DTNBP1	dystrobrevin binding protein 1	0.654264416	up	0.01331
DTWD2	DTW domain containing 2	-1.24347817	down	0.01518
DTX1	deltex 1, E3 ubiquitin ligase	1.666480685	up	1.58E-07
DTX3	deltex 3, E3 ubiquitin ligase	0.649422623	up	0.001608
DTYMK	deoxythymidylate kinase (thymidylate kinase)	-1.23454725	down	4.68E-09
DUSP10	dual specificity phosphatase 10	1.175613815	up	0.000416
DUSP15	dual specificity phosphatase 15	2.350447114	up	0.00078
DUSP16	dual specificity phosphatase 16	0.935391525	up	1.06E-08
DUSP19	dual specificity phosphatase 19	-2.7043932	down	1.21E-05

DUSP2	dual specificity phosphatase 2	3.134050123	up	0.036272
DUSP5	dual specificity phosphatase 5	1.644959164	up	0.001936
DUSP6	dual specificity phosphatase 6	1.180426579	up	1.08E-11
DUSP7	dual specificity phosphatase 7	1.079618221	up	1.2E-05
DUT	deoxyuridine triphosphatase	-1.06449652	down	7E-10
DYNLT1	dynein, light chain, Tctex-type 1	-0.66987677	down	0.001155
DYSF	dysferlin	1.459227389	up	0.036798
E2F1	E2F transcription factor 1	-2.5016173	down	6.51E-38
E2F2	E2F transcription factor 2	-1.93132114	down	2.49E-14
E2F5	E2F transcription factor 5, p130-binding	-1.65212403	down	2.58E-14
E2F7	E2F transcription factor 7	-1.04587253	down	1.16E-06
E2F8	E2F transcription factor 8	-1.67716632	down	3.61E-12
EARS2	glutamyl-tRNA synthetase 2, mitochondrial	-0.69677011	down	0.0045
EBAG9	estrogen receptor binding site associated, antigen, 9	-0.66395165	down	0.015148
EBI3	Epstein-Barr virus induced 3	1.128274195	up	9.94E-07
EBNA1BP2	EBNA1 binding protein 2	-0.82507548	down	2.52E-06
ECI2	enoyl-CoA delta isomerase 2	-2.4890615	down	2.08E-42
ECM1	extracellular matrix protein 1	0.868628227	up	0.01133
ECT2	epithelial cell transforming 2	-1.83162271	down	2.36E-20
EDEM3	ER degradation enhancer, mannosidase alpha-like 3	-0.7103568	down	0.000128
EDN1	endothelin 1	-1.4139057	down	2.39E-17
EEF1A2	eukaryotic translation elongation factor 1 alpha 2	0.678490719	up	6.76E-06
EEF2	eukaryotic translation elongation factor 2	0.611676264	up	3.62E-05
EEPD1	endonuclease/exonuclease/phosphatase family domain containing 1	0.610786183	up	0.00697

				7
EFCAB11	EF-hand calcium binding domain 11	-1.12989404	down	0.00141<br>6
EFL1	ephrin-A1	0.666789278	up	0.00037<br>5
EFNA3	ephrin-A3	0.818145559	up	0.00036<br>9
EFNB1	ephrin-B1	0.922522617	up	2.55E-0<br>5
EFNB2	ephrin-B2	0.757421526	up	0.00055<br>1
EFNB3	ephrin-B3	1.328484823	up	1.38E-0<br>9
EFR3A	EFR3 homolog A (S. cerevisiae)	-0.91901492	down	1.87E-0<br>6
EGFL7	EGF-like-domain, multiple 7	0.61639094	up	0.02903<br>6
EGR1	early growth response 1	2.453786174	up	0.00101<br>5
EGR2	early growth response 2	2.904373798	up	0.02794<br>1
EHD1	EH-domain containing 1	1.445184011	up	3.01E-1<br>2
EHF	ets homologous factor	-1.30001333	down	3.53E-0<br>6
EIF1	eukaryotic translation initiation factor 1	0.7671443	up	4.36E-0<br>6
EIF1AX	eukaryotic translation initiation factor 1A, X-linked	-1.27227302	down	7.51E-1<br>2
EIF1B	eukaryotic translation initiation factor 1B	0.669525359	up	0.00052
EIF2A	eukaryotic translation initiation factor 2A, 65kDa	0.643004797	up	0.00013<br>1
EIF2AK1	eukaryotic translation initiation factor 2-alpha kinase 1	-0.72316004	down	4.18E-0<br>6
EIF2AK2	eukaryotic translation initiation factor 2-alpha kinase 2	-1.05064269	down	8.08E-0<br>7
EIF4A2	eukaryotic translation initiation factor 4A2	0.929055597	up	0.00014<br>3
EIF4EBP2	eukaryotic translation initiation factor 4E binding protein 2	1.114111162	up	1.51E-1<br>2
EIF4EBP3	eukaryotic translation initiation factor 4E binding protein 3	0.795699596	up	0.01213<br>5
EIF4G1	eukaryotic translation initiation factor 4 gamma, 1	-1.16785209	down	2.58E-1

				4
ELF3	E74-like factor 3 (ets domain transcription factor, epithelial-specific)	0.891354103	up	0.02268
ELK4	ELK4, ETS-domain protein (SRF accessory protein 1)	-0.77587253	down	0.000363
ELL	elongation factor RNA polymerase II	1.298922966	up	1.33E-06
ELOVL1	ELOVL fatty acid elongase 1	0.627199817	up	0.000122
ELOVL4	ELOVL fatty acid elongase 4	0.983118031	up	0.003889
EME1	essential meiotic structure-specific endonuclease 1	-1.19114494	down	3.81E-10
EML2	echinoderm microtubule associated protein like 2	0.70302791	up	0.001255
EMP1	epithelial membrane protein 1	0.881522659	up	1.76E-07
EMP2	epithelial membrane protein 2	-1.09775903	down	4.79E-11
ENC1	ectodermal-neural cortex 1 (with BTB domain)	-1.06519593	down	7.32E-05
ENGASE	endo-beta-N-acetylglucosaminidase	0.629376511	up	0.002038
ENO2	enolase 2 (gamma, neuronal)	1.031092826	up	9.76E-12
ENPP4	ectonucleotide pyrophosphatase/phosphodiesterase 4 (putative)	2.020522754	up	0.000381
ENTPD1	ectonucleoside triphosphate diphosphohydrolase 1	1.609270201	up	0.018257
ENTPD2	ectonucleoside triphosphate diphosphohydrolase 2	1.253730615	up	0.00294
ENTPD8	ectonucleoside triphosphate diphosphohydrolase 8	1.527095778	up	0.006253
EOGT	EGF domain-specific O-linked N-acetylglucosamine (GlcNAc) transferase	-1.47898057	down	6.81E-05
EPC1	enhancer of polycomb homolog 1 (Drosophila)	0.748607843	up	0.000324
EPDR1	ependymin related 1	-1.00692923	down	1.4E-10
EPOR	erythropoietin receptor	0.59539182	up	0.004686
EPS15L1	epidermal growth factor receptor pathway substrate 15-like 1	0.725168055	up	0.000439
EPS8L3	EPS8-like 3	-1.74469821	down	0.000244
ERAL1	Era-like 12S mitochondrial rRNA chaperone 1	-0.63953979	down	0.00013

				7
ERAP1	endoplasmic reticulum aminopeptidase 1	-0.82330886	down	4.75E-05
ERC1	ELKS/RAB6-interacting/CAST family member 1	-0.86848559	down	0.029011
ERCC6L	excision repair cross-complementation group 6-like	-1.38825598	down	5.06E-08
ERCC6L2	excision repair cross-complementation group 6-like 2	-1.37056874	down	0.042549
EREG	epiregulin	1.178165587	up	0.005815
ERI3	ERI1 exoribonuclease family member 3	-0.68779878	down	0.001715
ERMAP	erythroblast membrane-associated protein (Scianna blood group)	-0.70423995	down	0.013118
ERMP1	endoplasmic reticulum metallopeptidase 1	-0.61864132	down	0.00045
ERN1	endoplasmic reticulum to nucleus signaling 1	1.538187283	up	1.29E-09
ESAM	endothelial cell adhesion molecule	1.185612966	up	6.25E-07
ETHE1	ethylmalonic encephalopathy 1	0.77910365	up	7.75E-07
ETNK2	ethanolamine kinase 2	0.833265496	up	3.39E-05
ETS1	v-ets avian erythroblastosis virus E26 oncogene homolog 1	0.780411956	up	0.001132
ETV3	ets variant 3	0.9119746	up	3.88E-05
EVC2	Ellis van Creveld syndrome 2	0.688694331	up	0.010122
EVI5	ecotropic viral integration site 5	-1.72389451	down	1.21E-09
EVI5L	ecotropic viral integration site 5-like	0.689165669	up	0.000484
EXO1	exonuclease 1	-1.83399551	down	5.64E-21
EXOC3L2	exocyst complex component 3-like 2	1.442242936	up	0.011897
EXOC5	exocyst complex component 5	-0.8957585	down	1.34E-06
EXOC6	exocyst complex component 6	-1.09906587	down	3.3E-09
EXOSC3	exosome component 3	-0.68365432	down	0.003155

EXOSC8	exosome component 8	-1.42652467	down	1.24E-09
EXTL3	exostosin-like glycosyltransferase 3	-0.73620344	down	1.2E-05
EZH1	enhancer of zeste 1 polycomb repressive complex 2 subunit	0.6609123	up	0.00033
EZH2	enhancer of zeste 2 polycomb repressive complex 2 subunit	-0.95352723	down	5.12E-07
F11R	F11 receptor	-1.19939449	down	2.85E-14
F3	coagulation factor III (thromboplastin, tissue factor)	1.082224832	up	0.000203
FA2H	fatty acid 2-hydroxylase	1.24969369	up	1.93E-05
FAAP24	chromosome 19 open reading frame 40	-0.91744777	down	9.97E-05
FADS1	fatty acid desaturase 1	-0.63250556	down	8.68E-05
FADS2	fatty acid desaturase 2	-1.47655642	down	2.37E-22
FADS3	fatty acid desaturase 3	0.952779406	up	7.64E-09
FAM102A	family with sequence similarity 102, member A	1.09669845	up	5.24E-14
FAM102B	family with sequence similarity 102, member B	-0.86198117	down	2.53E-05
FAM111A	family with sequence similarity 111, member A	-1.04991464	down	6.06E-07
FAM111B	family with sequence similarity 111, member B	-1.69918995	down	3.97E-05
FAM120A	family with sequence similarity 120A	-0.74586978	down	0.000189
FAM127B	family with sequence similarity 127, member B	-1.22717559	down	5.41E-14
FAM127C	family with sequence similarity 127, member C	-0.86557023	down	0.002695
FAM131C	family with sequence similarity 131, member C	0.716546756	up	0.001999
FAM149A	family with sequence similarity 149, member A	-0.70615756	down	0.039041
FAM160A2	family with sequence similarity 160, member A2	0.626238676	up	0.000588
FAM161B	family with sequence similarity 161, member B	1.557174379	up	7.31E-09
FAM168B	family with sequence similarity 168, member B	-0.59632967	down	0.00099

				1
FAM171A2	family with sequence similarity 171, member A2	0.837507375	up	0.000619
FAM172A	family with sequence similarity 172, member A	-1.00079893	down	0.003222
FAM173B	family with sequence similarity 173, member B	-1.19793444	down	4.65E-05
FAM175A	family with sequence similarity 175, member A	0.658672583	up	0.04521
FAM175B	family with sequence similarity 175, member B	-0.87887548	down	0.002293
FAM189A2	family with sequence similarity 189, member A2	2.353764444	up	0.022379
FAM196A	family with sequence similarity 196, member A	1.480687402	up	0.00652
FAM199X	family with sequence similarity 199, X-linked	-0.82037347	down	2.36E-06
FAM208A	family with sequence similarity 208, member A	-1.02781246	down	8.86E-09
FAM216A	family with sequence similarity 216, member A	-0.72092427	down	0.004354
FAM219A	family with sequence similarity 219, member A	0.727788641	up	0.001695
FAM221A	family with sequence similarity 221, member A	1.326037433	up	0.007605
FAM222B	family with sequence similarity 222, member B	0.75296139	up	1.8E-05
FAM24B	family with sequence similarity 24, member B	0.786772393	up	0.001523
FAM35A	family with sequence similarity 35, member A	-0.75207481	down	0.011578
FAM3C	family with sequence similarity 3, member C	0.692495574	up	0.012391
FAM46B	family with sequence similarity 46, member B	1.001081776	up	0.004802
FAM46C	family with sequence similarity 46, member C	1.814285376	up	0.027114
FAM49A	family with sequence similarity 49, member A	1.272539842	up	0.015934
FAM60A	family with sequence similarity 60, member A	-1.08096458	down	7.79E-10
FAM64A	family with sequence similarity 64, member A	-0.70168696	down	8.6E-05
FAM72A	family with sequence similarity 72, member A	-1.0421274	down	0.000121
FAM72C	family with sequence similarity 72, member C	-1.11788019	down	0.030116

FAM72D	family with sequence similarity 72, member D	-1.31351784	down	0.001828
FAM83A	family with sequence similarity 83, member A	-2.80309994	down	9.68E-05
FAM83D	family with sequence similarity 83, member D	-0.84881115	down	2.87E-07
FAM83G	family with sequence similarity 83, member G	0.900405638	up	4.79E-09
FAM83H	family with sequence similarity 83, member H	-0.63864281	down	0.011137
FAM86B2	family with sequence similarity 86, member B2	1.173439101	up	0.008126
FAM86C1	family with sequence similarity 86, member C1	-0.67061677	down	0.025971
FAM89B	family with sequence similarity 89, member B	0.626696081	up	0.019683
FAM98C	family with sequence similarity 98, member C	0.771662083	up	3.42E-05
FANCA	Fanconi anemia, complementation group A	-1.56084881	down	2.68E-19
FANCC	Fanconi anemia, complementation group C	-1.34124348	down	3.58E-09
FANCD2	Fanconi anemia, complementation group D2	-1.63162715	down	0.003036
FANCE	Fanconi anemia, complementation group E	0.618276762	up	0.00903
FANCI	Fanconi anemia, complementation group I	-1.04951634	down	8.77E-12
FANCM	Fanconi anemia, complementation group M	-0.75647158	down	0.028286
FAR1	fatty acyl CoA reductase 1	-2.03456687	down	0.0242
FASN	fatty acid synthase	-0.63904706	down	0.000232
FASTKD5	FAST kinase domains 5	-0.78017431	down	3.06E-05
FBF1	Fas (TNFRSF6) binding factor 1	-0.7903496	down	0.00033
FBN2	fibrillin 2	-1.15396479	down	3.54E-08
FBXL14	F-box and leucine-rich repeat protein 14	-0.81589818	down	0.000637
FBXL16	F-box and leucine-rich repeat protein 16	1.594431616	up	2.5E-12
FBXL17	F-box and leucine-rich repeat protein 17	-1.12725543	down	1.15E-05
FBXL18	F-box and leucine-rich repeat protein 18	-1.14307644	down	3.75E-0

				5
FBXL3	F-box and leucine-rich repeat protein 3	-1.06568434	down	0.01037<br>3
FBXL6	F-box and leucine-rich repeat protein 6	0.828126848	up	4.63E-0<br>5
FBXO31	F-box protein 31	0.839521	up	5.49E-0<br>7
FBXO32	F-box protein 32	1.623082605	up	1.21E-0<br>9
FBXO41	F-box protein 41	0.598114366	up	0.00187<br>7
FBXO43	F-box protein 43	-1.16285403	down	0.00551<br>2
FBXO44	F-box protein 44	0.696810596	up	0.00138<br>4
FBXO5	F-box protein 5	-1.22757792	down	6.83E-0<br>7
FBXO6	F-box protein 6	1.130648955	up	1.52E-0<br>8
FBXW5	F-box and WD repeat domain containing 5	0.886062896	up	1.35E-0<br>6
FCF1	FCF1 rRNA-processing protein	-0.78644457	down	3.5E-05
FCGBP	Fc fragment of IgG binding protein	-0.70288571	down	0.00368<br>5
FCGRT	Fc fragment of IgG, receptor, transporter, alpha	0.597577442	up	0.00058<br>1
FCHSD1	FCH and double SH3 domains 1	0.781296394	up	3.07E-0<br>5
FDPS	farnesyl diphosphate synthase	-1.02979065	down	1.14E-1<br>0
FECH	ferrochelatase	-2.14534556	down	3.58E-2<br>6
FEN1	flap structure-specific endonuclease 1	-1.50422735	down	1.73E-1<br>4
FERMT2	fermitin family member 2	0.719332519	up	2.95E-0<br>5
FEZ1	fasciculation and elongation protein zeta 1 (zygin I)	1.670788897	up	3.78E-0<br>8
FFAR2	free fatty acid receptor 2	1.658575812	up	0.0157
FGB	fibrinogen beta chain	-1.19743656	down	0.02259<br>6
FGF19	fibroblast growth factor 19	0.817187507	up	0.03304<br>1

FGG	fibrinogen gamma chain	-0.86807194	down	0.036369
FGL1	fibrinogen-like 1	-0.79450358	down	0.001869
FHL2	four and a half LIM domains 2	0.982612005	up	2.1E-10
FHL3	four and a half LIM domains 3	1.203072606	up	7.09E-10
FHOD3	formin homology 2 domain containing 3	1.081913127	up	0.001148
FIGN	fidgetin	-0.60700791	down	0.040181
FIGNL1	fidgetin-like 1	-1.34178497	down	1.08E-07
FKBP3	FK506 binding protein 3, 25kDa	-0.6485846	down	0.001804
FKBP5	FK506 binding protein 5	-0.78418437	down	0.008578
FKBP9	FK506 binding protein 9, 63 kDa	-0.94113821	down	1.19E-08
FKRP	fukutin related protein	-1.30240222	down	0.00011
FKTN	fukutin	-0.69751548	down	0.000731
FLOT1	flotillin 1	-0.80833975	down	4.35E-07
FLT3LG	fms-related tyrosine kinase 3 ligand	1.28242284	up	0.004108
FMR1	fragile X mental retardation 1	-1.13829547	down	2.85E-08
FN3K	fructosamine 3 kinase	0.727858398	up	0.001605
FNBP4	formin binding protein 4	0.66073399	up	0.011234
FNDC3A	fibronectin type III domain containing 3A	1.710203625	up	5.9E-05
FNTA	farnesyltransferase, CAAX box, alpha	0.781448491	up	0.002532
FOSB	FBJ murine osteosarcoma viral oncogene homolog B	3.168858565	up	6.42E-05
FOSL1	FOS-like antigen 1	0.949596618	up	0.030435
FOSL2	FOS-like antigen 2	0.613114766	up	0.001614
FOXA1	forkhead box A1	-1.23069755	down	0.000169

FOXM1	forkhead box M1	-1.3566446	down	2.96E-09
FOXN3	forkhead box N3	-0.75947632	down	0.000392
FOXRED1	FAD-dependent oxidoreductase domain containing 1	-0.82474265	down	0.000279
FOXRED2	FAD-dependent oxidoreductase domain containing 2	-0.74350317	down	0.004421
FRMD5	FERM domain containing 5	0.936309614	up	0.000148
FSBP	fibrinogen silencer binding protein	-0.83503057	down	0.009526
FSD1	fibronectin type III and SPRY domain containing 1	0.895606699	up	9.93E-06
FST	follistatin	0.887139869	up	0.002293
FTCD	formimidoyltransferase cyclodeaminase	4.077416197	up	0.026358
FTH1	ferritin, heavy polypeptide 1	1.115273084	up	1.04E-09
FUBP3	far upstream element (FUSE) binding protein 3	-0.74837238	down	9.09E-05
FUS	FUS RNA binding protein	-1.41471737	down	4.2E-05
FUT11	fucosyltransferase 11 (alpha (1,3) fucosyltransferase)	-0.96671469	down	0.024445
FXR2	fragile X mental retardation, autosomal homolog 2	0.612006181	up	0.000239
FXYD1	FXYD domain containing ion transport regulator 1	2.052103825	up	0.000239
FXYD7	FXYD domain containing ion transport regulator 7	5.743722704	up	2.99E-05
FYN	FYN proto-oncogene, Src family tyrosine kinase	0.833168386	up	0.014167
FZD3	frizzled class receptor 3	-1.57875407	down	0.001293
FZD4	frizzled class receptor 4	-1.23789254	down	9.17E-08
FZD8	frizzled class receptor 8	-0.91783218	down	0.001631
G0S2	G0/G1 switch 2	1.147489262	up	0.013471
G3BP1	GTPase activating protein (SH3 domain) binding protein 1	-0.6566846	down	5.63E-05

GAB2	GRB2-associated binding protein 2	0.647052988	up	0.001731
GABBR1	gamma-aminobutyric acid (GABA) B receptor, 1	0.771300648	up	0.01056
GABRA5	gamma-aminobutyric acid (GABA) A receptor, alpha 5	-0.66589717	down	0.000259
GABRD	gamma-aminobutyric acid (GABA) A receptor, delta	1.68643708	up	0.000222
GAD1	glutamate decarboxylase 1 (brain, 67kDa)	2.383721746	up	2.68E-12
GADD45A	growth arrest and DNA-damage-inducible, alpha	1.286808781	up	1.81E-06
GALE	UDP-galactose-4-epimerase	0.596167039	up	0.000214
GALNT18	polypeptide N-acetylgalactosaminyltransferase 18	0.666246299	up	0.001225
GALNT2	polypeptide N-acetylgalactosaminyltransferase 2	-0.63329243	down	8.94E-05
GALNT4	polypeptide N-acetylgalactosaminyltransferase 4	-1.52026976	down	0.008865
GALNT9	polypeptide N-acetylgalactosaminyltransferase 9	1.06804149	up	5.34E-08
GAPDH	glyceraldehyde-3-phosphate dehydrogenase	0.591093187	up	9.86E-05
GAS7	growth arrest-specific 7	1.019429939	up	0.005305
GATAD1	GATA zinc finger domain containing 1	0.591761616	up	0.001683
GATC	glutamyl-tRNA(Gln) amidotransferase, subunit C	-0.88983161	down	0.020307
GATSL3	GATS protein-like 3	1.696341158	up	4.44E-10
GBAS	glioblastoma amplified sequence	-1.67310217	down	3.23E-25
GBE1	glucan (1,4-alpha-), branching enzyme 1	-0.91669978	down	0.00151
GBP1	guanylate binding protein 1, interferon-inducible	0.909644591	up	0.0018
GBP2	guanylate binding protein 2, interferon-inducible	1.703064459	up	8.55E-21
GBP4	guanylate binding protein 4	1.124884419	up	0.021144
GBP5	guanylate binding protein 5	1.959414149	up	0.009939
GCC1	GRIP and coiled-coil domain containing 1	-0.62939299	down	0.000783

GCDH	glutaryl-CoA dehydrogenase	-1.10871278	down	0.009782
GCH1	GTP cyclohydrolase 1	0.673510218	up	0.021582
GCLC	glutamate-cysteine ligase, catalytic subunit	-0.81080268	down	0.000442
GCLM	glutamate-cysteine ligase, modifier subunit	-1.27629983	down	3.91E-13
GCNT3	glucosaminyl (N-acetyl) transferase 3, mucin type	0.663539958	up	3.33E-05
GCOM1	GRINL1A complex locus 1	1.275662643	up	2.43E-05
GDAP1	ganglioside induced differentiation associated protein 1	-0.90764881	down	0.000936
GDF15	growth differentiation factor 15	1.630573697	up	0.000575
GDI1	GDP dissociation inhibitor 1	0.68165718	up	1.09E-05
GEM	GTP binding protein overexpressed in skeletal muscle	2.035856477	up	2.62E-08
GEN1	GEN1 Holliday junction 5' flap endonuclease	-1.38151974	down	5.06E-05
GFPT2	glutamine-fructose-6-phosphate transaminase 2	1.178093346	up	1.46E-05
GFRA1	GDNF family receptor alpha 1	-0.70254901	down	0.020194
GFRA2	GDNF family receptor alpha 2	2.110879585	up	0.016315
GGA3	golgi-associated, gamma adaptin ear containing, ARF binding protein 3	0.601629199	up	0.000445
GGH	gamma-glutamyl hydrolase (conjugase, folylpolygammaglutamyl hydrolase)	-2.49949317	down	4.46E-22
GGT7	gamma-glutamyltransferase 7	0.66740122	up	0.000402
GIMAP2	GTPase, IMAP family member 2	1.403912734	up	0.026559
GINS1	GINS complex subunit 1 (Psf1 homolog)	-1.35741121	down	3.47E-14
GINS2	GINS complex subunit 2 (Psf2 homolog)	-1.78348832	down	3.61E-24
GINS4	GINS complex subunit 4 (Sld5 homolog)	-1.32032808	down	8.4E-11
GIPR	gastric inhibitory polypeptide receptor	1.120307826	up	0.018933

GLDC	glycine dehydrogenase (decarboxylating)	-1.29540794	down	2.47E-11
GLI3	GLI family zinc finger 3	1.48594325	up	0.037302
GLIPR1	GLI pathogenesis-related 1	1.235566349	up	5.64E-08
GLMP	chromosome 1 open reading frame 85	-1.17159579	down	2.82E-10
GLP2R	glucagon-like peptide 2 receptor	-1.51476945	down	0.003684
GLTSCR2	glioma tumor suppressor candidate region gene 2	0.66618544	up	0.000534
GLUL	glutamate-ammonia ligase	0.931898669	up	0.00023
GMIP	GEM interacting protein	0.611743842	up	0.014185
GMNN	geminin, DNA replication inhibitor	-0.78213251	down	0.000136
GNB4	guanine nucleotide binding protein (G protein), beta polypeptide 4	-1.68929119	down	3.54E-08
GNB5	guanine nucleotide binding protein (G protein), beta 5	-0.80648457	down	2.34E-06
GNG11	guanine nucleotide binding protein (G protein), gamma 11	-0.58619105	down	0.025559
GNG4	guanine nucleotide binding protein (G protein), gamma 4	-0.92440027	down	0.000232
GNL3L	guanine nucleotide binding protein-like 3 (nucleolar)-like	-0.60282388	down	0.002926
GNPAT	glyceronephosphate O-acyltransferase	-2.13156995	down	2.35E-35
GNPNAT1	glucosamine-phosphate N-acetyltransferase 1	-0.67013267	down	0.003237
GNPTAB	N-acetylglucosamine-1-phosphate transferase, alpha and beta subunits	-1.53818356	down	5.09E-16
GOLGA2	golgin A2	0.610346953	up	0.000138
GOLGA7B	golgin A7 family, member B	1.781821796	up	2.15E-15
GOLGB1	golgin B1	-0.87956454	down	0.004734
GOLIM4	golgi integral membrane protein 4	-0.90380624	down	8.76E-07
GOLM1	golgi membrane protein 1	-0.72623875	down	2.31E-06

GOLT1A	golgi transport 1A	2.068176853	up	3.43E-22
GOLT1B	golgi transport 1B	-0.68281444	down	0.003084
GORASP2	golgi reassembly stacking protein 2, 55kDa	-0.62181298	down	0.000205
GOT1	glutamic-oxaloacetic transaminase 1, soluble	-0.67321529	down	0.000122
GPAT3	1-acylglycerol-3-phosphate O-acyltransferase 9	0.810621902	up	2.28E-06
GPCPD1	glycerophosphocholine phosphodiesterase GDE1 homolog (S. cerevisiae)	1.11922747	up	1.17E-08
GPD1L	glycerol-3-phosphate dehydrogenase 1-like	-0.80317082	down	1.26E-05
GPD2	glycerol-3-phosphate dehydrogenase 2 (mitochondrial)	-0.8812402	down	1.74E-07
GPI	glucose-6-phosphate isomerase	0.831601155	up	2.9E-05
GPR108	G protein-coupled receptor 108	0.772015981	up	1.03E-06
GPR135	G protein-coupled receptor 135	-1.10765362	down	0.010411
GPR137B	G protein-coupled receptor 137B	1.031762377	up	8.29E-08
GPR153	G protein-coupled receptor 153	0.926884962	up	2.39E-05
GPR155	G protein-coupled receptor 155	0.673989437	up	0.0246
GPR156	G protein-coupled receptor 156	3.032946598	up	0.002596
GPR157	G protein-coupled receptor 157	1.144152415	up	3.87E-08
GPR162	G protein-coupled receptor 162	0.683958132	up	0.032951
GPR3	G protein-coupled receptor 3	0.913993347	up	0.005899
GPR4	G protein-coupled receptor 4	2.417122177	up	0.012319
GPRC5A	G protein-coupled receptor, class C, group 5, member A	-0.6191441	down	0.017251
GPRC5C	G protein-coupled receptor, class C, group 5, member C	0.643648059	up	5.06E-05
GPSM1	G-protein signaling modulator 1	0.849359249	up	0.000156
GPSM2	G-protein signaling modulator 2	-0.70605405	down	0.0002

GPSM3	G-protein signaling modulator 3	1.007744344	up	0.037725
GPX2	glutathione peroxidase 2 (gastrointestinal)	-0.99646251	down	5.92E-11
GPX3	glutathione peroxidase 3 (plasma)	0.6686005	up	0.00082
GPX4	glutathione peroxidase 4	0.622583779	up	0.000211
GPX8	glutathione peroxidase 8 (putative)	-0.5977218	down	0.012887
GRAMD1A	GRAM domain containing 1A	0.716634349	up	4.01E-05
GRASP	GRP1 (general receptor for phosphoinositides 1)-associated scaffold protein	1.755340093	up	0.010407
GRB10	growth factor receptor-bound protein 10	0.624751412	up	0.00012
GRB14	growth factor receptor-bound protein 14	0.605368434	up	0.002374
GRB2	growth factor receptor-bound protein 2	-0.87484588	down	3.28E-08
GRB7	growth factor receptor-bound protein 7	1.104484084	up	0.00033
GREM2	gremlin 2, DAN family BMP antagonist	2.284268212	up	6.59E-09
GRHL3	grainyhead-like 3 (Drosophila)	0.953333717	up	0.00688
GRIN3B	glutamate receptor, ionotropic, N-methyl-D-aspartate 3B	0.924035802	up	0.000861
GRINA	glutamate receptor, ionotropic, N-methyl D-aspartate-associated protein 1 (glutamate binding)	0.822933225	up	4E-08
GRM4	glutamate receptor, metabotropic 4	2.247315473	up	0.009614
GRN	granulin	0.631199666	up	2.08E-05
GS1-259H13.2	transmembrane protein 225-like	0.906600115	up	0.040181
GSG2	germ cell associated 2 (haspin)	-1.21544104	down	1.96E-08
GSR	glutathione reductase	-1.04210824	down	4.3E-12
GTF2B	general transcription factor IIB	0.827743786	up	1.41E-05
GTF2H3	general transcription factor IIH, polypeptide 3, 34kDa	-0.75698729	down	6.21E-05
GTF2I	general transcription factor IIi	-0.67527548	down	0.000859
GTF2IRD2	GTF2I repeat domain containing 2	-1.1380392	down	0.049053

GTPBP1	GTP binding protein 1	0.889599259	up	0.00107
GTSE1	G-2 and S-phase expressed 1	-1.30150137	down	3.23E-12
GUCY1A2	guanylate cyclase 1, soluble, alpha 2	-1.63741129	down	0.010695
GUCY1B3	guanylate cyclase 1, soluble, beta 3	0.902757855	up	0.029468
GYG2	glycogenin 2	-0.99580764	down	8.52E-05
H1F0	H1 histone family, member 0	1.151166873	up	4.92E-15
H1FX	H1 histone family, member X	0.710593609	up	0.006516
H2AFV	H2A histone family, member V	-0.92777221	down	4.32E-09
H2AFX	H2A histone family, member X	-2.31223602	down	8.8E-35
H2AFZ	H2A histone family, member Z	-1.40140148	down	8.58E-10
H6PD	hexose-6-phosphate dehydrogenase (glucose 1-dehydrogenase)	-0.8522581	down	1.18E-07
HACD2	protein tyrosine phosphatase-like (proline instead of catalytic arginine), member b	-1.24015052	down	4.07E-14
HAL	histidine ammonia-lyase	1.324762785	up	0.036494
HAND1	heart and neural crest derivatives expressed 1	1.668816021	up	0.006556
HAP1	huntingtin-associated protein 1	0.926595275	up	0.000747
HAPLN3	hyaluronan and proteoglycan link protein 3	0.790047598	up	0.002268
HAS2	hyaluronan synthase 2	0.692242379	up	0.042867
HAS3	hyaluronan synthase 3	1.475450635	up	3.54E-23
HAT1	histone acetyltransferase 1	-1.85867242	down	2.47E-10
HAVCR1	hepatitis A virus cellular receptor 1	-0.61825192	down	0.00102
HBP1	HMG-box transcription factor 1	0.747015618	up	4.42E-06
HBS1L	HBS1-like translational GTPase	-0.61600115	down	0.023579
HCFC1	host cell factor C1	-0.83714274	down	5.63E-06

HCFC2	host cell factor C2	0.71804635	up	0.01823
HCN2	hyperpolarization activated cyclic nucleotide-gated potassium channel 2	0.777997342	up	0.00094 9
HCN4	hyperpolarization activated cyclic nucleotide-gated potassium channel 4	1.624527908	up	0.00537 3
HDAC1	histone deacetylase 1	-0.78857478	down	7.08E-0 7
HDAC11	histone deacetylase 11	0.799588085	up	0.00011 3
HDAC4	histone deacetylase 4	-0.59135543	down	0.01655
HDAC5	histone deacetylase 5	1.304156554	up	5.86E-1 0
HDAC8	histone deacetylase 8	-0.69962035	down	0.02114 4
HEATR5A	HEAT repeat containing 5A	-0.66996095	down	0.00116 6
HELLS	helicase, lymphoid-specific	-1.33640839	down	2.05E-1 1
HELZ2	helicase with zinc finger 2, transcriptional coactivator	0.765120745	up	4.29E-0 7
HERC2	HECT and RLD domain containing E3 ubiquitin protein ligase 2	-0.62024401	down	0.00639 5
HERC5	HECT and RLD domain containing E3 ubiquitin protein ligase 5	1.165312772	up	6.13E-0 8
HES2	hes family bHLH transcription factor 2	0.660928731	up	0.02304 5
HEXIM1	hexamethylene bis-acetamide inducible 1	-0.79097618	down	1.58E-0 6
HFE	hemochromatosis	-2.42909165	down	1.05E-3 3
HGD	homogentisate 1,2-dioxygenase	-1.92561891	down	9.31E-1 1
HID1	HID1 domain containing	0.685144536	up	8.4E-05
HINT3	histidine triad nucleotide binding protein 3	-0.63499571	down	0.00776
HIPK1	homeodomain interacting protein kinase 1	-1.33753402	down	4.83E-0 9
HIPK2	homeodomain interacting protein kinase 2	-0.6249574	down	0.04167 9
HIST1H1C	histone cluster 1, H1c	2.041123572	up	2.89E-3 0
HIST1H2AC	histone cluster 1, H2ac	0.774356598	up	0.00251 7
HIST1H2BC	histone cluster 1, H2bc	1.742745399	up	4.89E-0

				9
HIST1H2BD	histone cluster 1, H2bd	0.614555324	up	0.01948
HIST1H2BJ	histone cluster 1, H2bj	1.247907143	up	0.018206
HIST2H2AA3	histone cluster 2, H2aa3	2.314322593	up	0.002112
HIST2H2BE	histone cluster 2, H2be	1.65712118	up	4.16E-10
HIST2H4A	histone cluster 2, H4a	2.602986355	up	0.007045
HIST2H4B	histone cluster 2, H4b	2.945827261	up	0.00883
HIST3H2A	histone cluster 3, H2a	1.028279489	up	0.005554
HKR1	HKR1, GLI-Kruppel zinc finger family member	1.002927241	up	0.004359
HLA-A	major histocompatibility complex, class I, A	1.174469432	up	1.84E-15
HLA-B	major histocompatibility complex, class I, B	1.888164085	up	4.52E-25
HLA-C	major histocompatibility complex, class I, C	0.710054671	up	3.43E-06
HLA-DMB	major histocompatibility complex, class II, DM beta	-0.75335095	down	0.000737
HLA-E	major histocompatibility complex, class I, E	0.774100072	up	2.39E-07
HLA-F	major histocompatibility complex, class I, F	1.626657473	up	1.23E-21
HLCS	holocarboxylase synthetase (biotin-(proprionyl-CoA-carboxylase (ATP-hydrolysing)) ligase)	-0.60846272	down	0.022405
HLF	hepatic leukemia factor	-0.83750385	down	0.013513
HM13	histocompatibility (minor) 13	0.80243408	up	6.43E-07
HMG20A	high mobility group 20A	-1.14594113	down	4.84E-12
HMGA2	high mobility group AT-hook 2	1.564994511	up	9.85E-12
HMGB1	high mobility group box 1	-1.14477273	down	1.07E-13
HMGB2	high mobility group box 2	-1.64474199	down	9E-27
HMGB3	high mobility group box 3	-0.99839005	down	3.09E-06
HMGCS1	3-hydroxy-3-methylglutaryl-CoA synthase 1 (soluble)	-0.83498947	down	0.00099

				7
HMGN3	high mobility group nucleosomal binding domain 3	-0.66236543	down	0.008383
HMGN4	high mobility group nucleosomal binding domain 4	-0.84953592	down	2.11E-07
HMGN5	high mobility group nucleosome binding domain 5	-1.8858665	down	0.000704
HMGXB3	HMG box domain containing 3	0.612130385	up	0.003733
HMMR	hyaluronan-mediated motility receptor (RHAMM)	-1.21995963	down	5.82E-12
HN1L	hematological and neurological expressed 1-like	-0.64753343	down	9.87E-05
HNF4G	hepatocyte nuclear factor 4, gamma	-1.96836121	down	0.028462
HNRNPA2B1	heterogeneous nuclear ribonucleoprotein A2/B1	-1.13458537	down	2.34E-11
HNRNPAB	heterogeneous nuclear ribonucleoprotein A/B	-1.0226752	down	1.6E-05
HNRNPD	heterogeneous nuclear ribonucleoprotein D (AU-rich element RNA binding protein 1, 37kDa)	-0.95062095	down	1.55E-09
HNRNPH3	heterogeneous nuclear ribonucleoprotein H3 (2H9)	-1.07010032	down	2.18E-11
HNRNPM	heterogeneous nuclear ribonucleoprotein M	-0.82526817	down	0.00153
HNRNPR	heterogeneous nuclear ribonucleoprotein R	-0.77106702	down	5.41E-07
HOGA1	4-hydroxy-2-oxoglutarate aldolase 1	-1.33016779	down	0.006374
HOMER1	homer homolog 1 (Drosophila)	-2.24774344	down	5.39E-10
HOOK3	hook microtubule-tethering protein 3	-0.95369908	down	0.000156
HOXA1	homeobox A1	1.079864957	up	1.63E-06
HOXB5	homeobox B5	-1.0317709	down	0.010758
HP1BP3	heterochromatin protein 1, binding protein 3	-0.80523691	down	2.11E-07
HPCAL1	hippocalcin-like 1	0.806437263	up	0.000924
HPCAL4	hippocalcin like 4	3.435284836	up	1.15E-20
HPD	4-hydroxyphenylpyruvate dioxygenase	-1.01670269	down	0.016869

HPGD	hydroxyprostaglandin dehydrogenase 15-(NAD)	0.620826229	up	0.043993
HPS3	Hermansky-Pudlak syndrome 3	0.755691253	up	2.05E-06
HSBP1L1	heat shock factor binding protein 1-like 1	0.651020497	up	0.002535
HSD17B12	hydroxysteroid (17-beta) dehydrogenase 12	-0.94583153	down	0.001548
HSH2D	hematopoietic SH2 domain containing	1.064675889	up	6.97E-06
HSP90AA1	heat shock protein 90kDa alpha (cytosolic), class A member 1	-1.02874484	down	6.14E-12
HSPA2	heat shock 70kDa protein 2	-1.12358515	down	5.44E-06
HSPB8	heat shock 22kDa protein 8	0.888863247	up	0.008318
HSPE1	heat shock 10kDa protein 1	-1.83834851	down	1.46E-28
HSPE1-MOB4	HSPE1-MOB4 readthrough	-1.88359598	down	1.6E-22
HTATSF1	HIV-1 Tat specific factor 1	-1.06304178	down	8.14E-11
HTR1B	5-hydroxytryptamine (serotonin) receptor 1B, G protein-coupled	-1.28591975	down	0.040889
HTR1D	5-hydroxytryptamine (serotonin) receptor 1D, G protein-coupled	-2.24619345	down	1.91E-25
HYLS1	hydrolethalus syndrome 1	-0.70201589	down	0.001598
IARS	isoleucyl-tRNA synthetase	-0.92775477	down	9.42E-10
IBTK	inhibitor of Bruton agammaglobulinemia tyrosine kinase	-1.00398691	down	9.26E-08
ICA1	islet cell autoantigen 1, 69kDa	0.593929571	up	0.038817
ICAM1	intercellular adhesion molecule 1	1.302640997	up	0.000102
ICAM5	intercellular adhesion molecule 5, telencephalin	0.978620761	up	0.003488
ICE2	interactor of little elongator complex ELL subunit 2	-0.86625246	down	8.93E-07
ICK	intestinal cell (MAK-like) kinase	-2.14022672	down	1.42E-11
ICMT	isoprenylcysteine carboxyl methyltransferase	-0.8467803	down	7.69E-06

ICOSLG	inducible T-cell co-stimulator ligand	1.73458711	up	0.000332
IDH1	isocitrate dehydrogenase 1 (NADP+), soluble	-1.06811325	down	3.78E-13
IDI1	isopentenyl-diphosphate delta isomerase 1	-1.21000659	down	5.62E-12
IER3	immediate early response 3	1.063775794	up	0.00294
IFI27	interferon, alpha-inducible protein 27	1.857869303	up	1.7E-17
IFI44	interferon-induced protein 44	0.9417678	up	4.32E-06
IFI44L	interferon-induced protein 44-like	0.657245082	up	0.001287
IFI6	interferon, alpha-inducible protein 6	0.945771372	up	1.37E-06
IFIH1	interferon induced with helicase C domain 1	0.687741113	up	0.017771
IFIT3	interferon-induced protein with tetratricopeptide repeats 3	1.34085679	up	0.000764
IFNGR1	interferon gamma receptor 1	0.938862078	up	1.74E-06
IFNGR2	interferon gamma receptor 2 (interferon gamma transducer 1)	0.759243548	up	2.54E-06
IFT81	intraflagellar transport 81	-1.29475262	down	4.36E-11
IGBP1	immunoglobulin (CD79A) binding protein 1	0.625915531	up	0.000244
IGF2BP2	insulin-like growth factor 2 mRNA binding protein 2	0.596663028	up	0.001142
IGF2BP3	insulin-like growth factor 2 mRNA binding protein 3	-0.79132325	down	2.24E-06
IGFBP3	insulin-like growth factor binding protein 3	0.735145063	up	3.32E-05
IGFLR1	IGF-like family receptor 1	-1.44892009	down	4.7E-05
IGSF10	immunoglobulin superfamily, member 10	-1.29868345	down	0.000313
IGSF3	immunoglobulin superfamily, member 3	-0.96092601	down	9.43E-09
IGSF8	immunoglobulin superfamily, member 8	0.641036413	up	0.010567
IKBIP	IKBKB interacting protein	-1.14199183	down	7.64E-05
IKBKE	inhibitor of kappa light polypeptide gene enhancer in B-cells, kinase epsilon	0.664312748	up	0.003512

IL10RA	interleukin 10 receptor, alpha	2.501516054	up	0.010405
IL10RB	interleukin 10 receptor, beta	1.006619338	up	1.84E-09
IL15	interleukin 15	0.669746581	up	0.027655
IL1A	interleukin 1, alpha	1.930779882	up	3.77E-06
IL1B	interleukin 1, beta	1.161909386	up	5.62E-05
IL1RAP	interleukin 1 receptor accessory protein	0.937370171	up	0.002211
IL22RA1	interleukin 22 receptor, alpha 1	1.184573816	up	7.4E-09
IL32	interleukin 32	1.727014521	up	9.87E-09
IL4I1	interleukin 4 induced 1	1.636374957	up	2.51E-06
IL4R	interleukin 4 receptor	0.943680662	up	2.07E-09
IL6	interleukin 6	1.348765711	up	0.033626
IL6ST	interleukin 6 signal transducer	-0.79090477	down	0.003816
ILF2	interleukin enhancer binding factor 2	-0.9488634	down	3.21E-10
IMPA2	inositol(myo)-1(or 4)-monophosphatase 2	-0.61846583	down	0.000463
IMPAD1	inositol monophosphatase domain containing 1	-0.7021409	down	2.38E-05
INCENP	inner centromere protein antigens 135/155kDa	-1.58955284	down	1.15E-22
ING3	inhibitor of growth family, member 3	1.018564392	up	0.013336
ING4	inhibitor of growth family, member 4	0.655556554	up	0.000809
INHBA	inhibin, beta A	1.671401074	up	3.2E-06
INHBB	inhibin, beta B	-1.13598932	down	1.79E-12
INO80	INO80 complex subunit	-0.64418432	down	0.00158
INO80C	INO80 complex subunit C	-2.0588546	down	5.79E-24
INPP1	inositol polyphosphate-1-phosphatase	0.593822748	up	0.004557

INPP5K	inositol polyphosphate-5-phosphatase K	0.632184466	up	0.00015
INSL4	insulin-like 4 (placenta)	-2.44941003	down	3.04E-29
INTS12	integrator complex subunit 12	0.737222275	up	0.000762
INTS2	integrator complex subunit 2	-1.22146555	down	1.91E-11
IPO4	importin 4	-1.03396241	down	1.92E-10
IPO8	importin 8	-0.75484879	down	0.000323
IPP	intracisternal A particle-promoted polypeptide	-1.24918465	down	8.06E-05
IRAK2	interleukin-1 receptor-associated kinase 2	1.417514057	up	0.000199
IRF1	interferon regulatory factor 1	0.997399006	up	0.005099
IRF4	interferon regulatory factor 4	2.097823584	up	0.020308
IRF5	interferon regulatory factor 5	0.597786783	up	0.004159
ISG15	ISG15 ubiquitin-like modifier	1.524709908	up	8.41E-11
ISG20	interferon stimulated exonuclease gene 20kDa	1.006127999	up	2.81E-08
ISL2	ISL LIM homeobox 2	0.825942131	up	0.004531
ISM2	isthmin 2	1.580635485	up	0.000525
ITGA2	integrin, alpha 2 (CD49B, alpha 2 subunit of VLA-2 receptor)	1.759166434	up	9.14E-32
ITGA5	integrin, alpha 5 (fibronectin receptor, alpha polypeptide)	1.17596048	up	8.8E-16
ITGA7	integrin, alpha 7	1.050587537	up	0.005436
ITGAV	integrin, alpha V	-0.79319329	down	4.44E-07
ITGAX	integrin, alpha X (complement component 3 receptor 4 subunit)	1.410503697	up	1.95E-06
ITGB4	integrin, beta 4	0.912192224	up	1.74E-07
ITPKC	inositol-trisphosphate 3-kinase C	0.900746779	up	4.38E-08
ITPR3	inositol 1,4,5-trisphosphate receptor, type 3	0.914516001	up	0.03581

				1
ITPRIP	inositol 1,4,5-trisphosphate receptor interacting protein	0.795118092	up	0.018768
ITPRIPL2	inositol 1,4,5-trisphosphate receptor interacting protein-like 2	-1.06932845	down	5.17E-05
JAG1	jagged 1	0.904162359	up	2.66E-09
JAK3	Janus kinase 3	0.701711906	up	0.030923
JARID2	jumonji, AT rich interactive domain 2	1.505642896	up	0.010745
JAZF1	JAZF zinc finger 1	0.810054503	up	0.00158
JDP2	Jun dimerization protein 2	-0.75634434	down	0.000356
JOSD2	Josephin domain containing 2	0.701030001	up	0.032439
JUN	jun proto-oncogene	1.677161187	up	0.000355
JUNB	jun B proto-oncogene	0.884647517	up	0.049053
KAT2B	K(lysine) acetyltransferase 2B	-0.96620833	down	0.000291
KATNAL1	katanin p60 subunit A-like 1	-0.80282818	down	0.00459
KATNAL2	katanin p60 subunit A-like 2	-1.78005052	down	0.030371
KAZALD1	Kazal-type serine peptidase inhibitor domain 1	-0.61834748	down	0.024494
KCNC3	potassium voltage-gated channel, Shaw-related subfamily, member 3	1.085111913	up	0.001943
KCNE4	potassium voltage-gated channel, Isk-related family, member 4	-4.78604489	down	5.01E-05
KCNF1	potassium voltage-gated channel, subfamily F, member 1	0.722208975	up	0.020271
KCNH2	potassium voltage-gated channel, subfamily H (eag-related), member 2	1.005866103	up	0.001431
KCNH3	potassium voltage-gated channel, subfamily H (eag-related), member 3	0.589052169	up	0.019681
KCNJ4	potassium inwardly-rectifying channel, subfamily J, member 4	1.429893933	up	0.004161
KCNK10	potassium channel, subfamily K, member 10	2.825781726	up	0.047979
KCNK5	potassium channel, subfamily K, member 5	-0.75726583	down	0.041994

KCNN1	potassium intermediate/small conductance calcium-activated channel, subfamily N, member 1	0.830888318	up	0.026602
KCNN4	potassium intermediate/small conductance calcium-activated channel, subfamily N, member 4	0.709339531	up	1.48E-05
KCNS3	potassium voltage-gated channel, delayed-rectifier, subfamily S, member 3	-2.09273128	down	1.98E-20
KCTD15	potassium channel tetramerization domain containing 15	-0.81837093	down	3.97E-05
KCTD21	potassium channel tetramerization domain containing 21	0.746614098	up	0.000183
KCTD3	potassium channel tetramerization domain containing 3	-0.72630918	down	1.03E-05
KCTD9	potassium channel tetramerization domain containing 9	0.826172942	up	0.000281
KDELC2	KDEL (Lys-Asp-Glu-Leu) containing 2	-0.90117125	down	0.038476
KDM4B	lysine (K)-specific demethylase 4B	0.863849486	up	7.05E-07
KDM5B	lysine (K)-specific demethylase 5B	0.894916194	up	6.81E-09
KDM6B	lysine (K)-specific demethylase 6B	1.067630728	up	2.4E-06
KEAP1	kelch-like ECH-associated protein 1	-1.12249583	down	2.29E-11
KIAA0586	KIAA0586	-1.33338822	down	0.000142
KIAA1147	KIAA1147	-0.63664731	down	0.001831
KIAA1161	KIAA1161	-0.75601391	down	7.38E-05
KIAA1524	KIAA1524	-1.11921294	down	2.51E-08
KIAA1549L	KIAA1549-like	-0.78938051	down	0.000126
KIAA1683	KIAA1683	1.450856724	up	0.038862
KIF11	kinesin family member 11	-1.60778011	down	0.000274
KIF14	kinesin family member 14	-2.1705826	down	8.76E-14
KIF15	kinesin family member 15	-1.26804244	down	0.000131
KIF17	kinesin family member 17	0.955257815	up	0.000885

KIF18A	kinesin family member 18A	-0.84302378	down	0.049259
KIF18B	kinesin family member 18B	-1.37591352	down	1.57E-12
KIF1A	kinesin family member 1A	2.238176053	up	4.26E-15
KIF20A	kinesin family member 20A	-0.99967195	down	4.26E-11
KIF20B	kinesin family member 20B	-2.16204459	down	6.62E-06
KIF21B	kinesin family member 21B	1.081698069	up	8.46E-08
KIF22	kinesin family member 22	-0.59261656	down	0.000547
KIF23	kinesin family member 23	-1.1498829	down	8.59E-08
KIF24	kinesin family member 24	-0.65996106	down	0.008702
KIF2C	kinesin family member 2C	-0.88791884	down	1.89E-08
KIF3B	kinesin family member 3B	-2.17950126	down	2.29E-42
KIF4A	kinesin family member 4A	-1.45168544	down	1.97E-09
KIF4B	kinesin family member 4B	-1.61952909	down	1.06E-07
KIF5A	kinesin family member 5A	1.318044583	up	0.009962
KIFC1	kinesin family member C1	-0.9831762	down	8.84E-06
KIRREL2	kin of IRRE like 2 (Drosophila)	1.108648769	up	2.69E-07
KISS1	KiSS-1 metastasis-suppressor	2.609836886	up	0.004092
KITLG	KIT ligand	-1.4474503	down	5.92E-11
KLC3	kinesin light chain 3	1.216979402	up	0.001516
KLF12	Kruppel-like factor 12	-1.09359203	down	1.52E-05
KLF3	Kruppel-like factor 3 (basic)	0.630786967	up	0.000269
KLF4	Kruppel-like factor 4 (gut)	1.398016376	up	6.32E-17

KLF6	Kruppel-like factor 6	1.189455542	up	0.001721
KLHDC7A	kelch domain containing 7A	-1.16446128	down	0.000575
KLHL21	kelch-like family member 21	1.292177187	up	7.76E-13
KLHL24	kelch-like family member 24	1.818011773	up	0.028366
KLHL26	kelch-like family member 26	0.782410157	up	0.006766
KLHL35	kelch-like family member 35	1.520984847	up	0.017554
KLHL38	kelch-like family member 38	4.096883021	up	0.000401
KLRC2	killer cell lectin-like receptor subfamily C, member 2	1.34475233	up	0.013227
KLRC3	killer cell lectin-like receptor subfamily C, member 3	2.097466416	up	0.003776
KLRG1	killer cell lectin-like receptor subfamily G, member 1	-2.37494876	down	0.030664
KNSTRN	kinetochore-localized astrin/SPAG5 binding protein	-0.84442872	down	3.83E-06
KNTC1	kinetochore associated 1	-1.44264086	down	1.85E-14
KPNA2	karyopherin alpha 2 (RAG cohort 1, importin alpha 1)	-0.80434756	down	7.52E-08
KRAS	Kirsten rat sarcoma viral oncogene homolog	-0.77171934	down	0.000628
KRBOX4	KRAB box domain containing 4	1.439235911	up	0.002876
KREMEN1	kringle containing transmembrane protein 1	-0.71786305	down	0.045121
KREMEN2	kringle containing transmembrane protein 2	0.588149539	up	0.025179
KRT16	keratin 16, type I	1.937327386	up	0.027701
KRT18	keratin 18, type I	-0.89571307	down	2.21E-09
KRT19	keratin 19, type I	0.671541135	up	3.17E-05
KRT23	keratin 23 (histone deacetylase inducible)	1.368716891	up	0.00459
KRT32	keratin 32, type I	2.437206992	up	0.022084

KRT4	keratin 4, type II	-4.38859376	down	1.19E-12
KRT83	keratin 83, type II	-0.77786539	down	0.000581
KSR2	kinase suppressor of ras 2	-1.10996647	down	0.009188
L1CAM	L1 cell adhesion molecule	1.484574639	up	1.17E-19
L3HYPDH	L-3-hydroxyproline dehydratase (trans-)	-0.99426604	down	1.35E-07
L3MBTL2	l(3)mbt-like 2 (Drosophila)	-0.99841843	down	0.00055
LACTB2	lactamase, beta 2	-1.03037687	down	5.41E-07
LAMA4	laminin, alpha 4	2.512914469	up	0.004136
LAMB3	laminin, beta 3	0.731379135	up	1.02E-05
LAMC2	laminin, gamma 2	1.292474486	up	5.82E-17
LAMP3	lysosomal-associated membrane protein 3	1.748334963	up	7.91E-08
LANCL1	LanC lantibiotic synthetase component C-like 1 (bacterial)	-0.7269657	down	2.32E-05
LARP7	La ribonucleoprotein domain family, member 7	-0.84447489	down	0.00027
LARS2	leucyl-tRNA synthetase 2, mitochondrial	-2.22894405	down	1.73E-14
LAS1L	LAS1-like (S. cerevisiae)	-0.76970767	down	2.87E-06
LASP1	LIM and SH3 protein 1	-0.85690597	down	1.59E-07
LAT2	linker for activation of T cells family, member 2	2.194364085	up	3.08E-18
LATS1	large tumor suppressor kinase 1	-0.71056737	down	0.002838
LAYN	layilin	0.788585465	up	0.009534
LBH	limb bud and heart development	-1.69204202	down	7.11E-26
LBR	lamin B receptor	-0.60031993	down	0.034632
LCMT2	leucine carboxyl methyltransferase 2	-0.82041913	down	0.012643
LCP1	lymphocyte cytosolic protein 1 (L-plastin)	3.109472287	up	9.9E-05

LDHA	lactate dehydrogenase A	-0.87788303	down	2.97E-09
LDHD	lactate dehydrogenase D	0.651929992	up	0.005107
LDOC1L	leucine zipper, down-regulated in cancer 1-like	0.759446115	up	3.92E-06
LEO1	Leo1, Paf1/RNA polymerase II complex component, homolog (S. cerevisiae)	-0.86267958	down	5.71E-07
LETMD1	LETM1 domain containing 1	1.013148244	up	5.05E-10
LGALS3BP	lectin, galactoside-binding, soluble, 3 binding protein	0.598408923	up	0.000371
LGI4	leucine-rich repeat LGI family, member 4	1.857502899	up	0.021377
LGMN	legumain	-0.74050991	down	5.67E-06
LHX6	LIM homeobox 6	1.027672593	up	0.035198
LIMA1	LIM domain and actin binding 1	0.913753817	up	1.46E-05
LIMCH1	LIM and calponin homology domains 1	-1.84365575	down	5.51E-06
LIMD2	LIM domain containing 2	0.92382578	up	2.24E-05
LIMK1	LIM domain kinase 1	0.607604088	up	0.000153
LIN37	lin-37 DREAM MuvB core complex component	0.923741556	up	4.36E-05
LIN7B	lin-7 homolog B (C. elegans)	0.971371726	up	0.000607
LIPH	lipase, member H	0.780071759	up	1.21E-05
LIPT2	lipoyl(octanoyl) transferase 2 (putative)	-1.02877125	down	0.032439
LMBR1L	limb development membrane protein 1-like	1.436304895	up	1.06E-12
LMBRD2	LMBR1 domain containing 2	-1.06881024	down	8.77E-05
LMNB1	lamin B1	-2.32335066	down	7.18E-41
LMNB2	lamin B2	-1.18008928	down	6.5E-14
LMO2	LIM domain only 2 (rhombotin-like 1)	1.305862303	up	0.049093

LMO4	LIM domain only 4	-0.92519548	down	1.02E-06
LMTK3	lemur tyrosine kinase 3	0.705138407	up	0.02282
LMX1B	LIM homeobox transcription factor 1, beta	0.866649702	up	8.29E-05
LOC100130370	uncharacterized LOC100130370	-1.07625256	down	0.001119
LOC101927572	uncharacterized LOC101927572	-0.98787526	down	0.033393
LOC102723728	nodal modulator 3-like	1.16155577	up	0.039489
LOC102723899	pituitary tumor-transforming gene 1 protein-interacting protein-like	-1.21669412	down	0.033419
LOC102724378	uncharacterized LOC102724378	-0.93509636	down	0.0159
LOC102724642	anaphase-promoting complex subunit 1-like	-1.22444925	down	0.005838
LOC102724965	collagen alpha-2(I) chain-like	-1.29757437	down	0.033691
LOC105369201	uncharacterized LOC105369201	1.261302187	up	6E-06
LOC105370708	uncharacterized LOC105370708	-2.18650575	down	0.048359
LOC105371921	uncharacterized LOC105371921	2.952523597	up	0.011372
LOC105373989	serine/arginine repetitive matrix protein 1-like	-1.48673777	down	0.001457
LOC105374378	uncharacterized LOC105374378	1.12521496	up	0.007386
LOC105374811	uncharacterized LOC105374811	-2.10192244	down	0.021517
LOC105375907	uncharacterized LOC105375907	-2.19621534	down	0.0201
LOC105376488	uncharacterized LOC105376488	1.283778813	up	0.006341
LOC388282	uncharacterized LOC388282	-1.00762	down	0.000108
LOC401052	uncharacterized LOC401052	1.481756327	up	0.000644
LOC402096	uncharacterized LOC402096	-1.699587	down	0.011208
LOC441155	zinc finger CCCH-type domain-containing-like	-0.94306087	down	0.001905
LOC728026	prothymosin alpha-like	-1.36897162	down	0.022355
LOC728392	uncharacterized LOC728392	1.166427665	up	0.00425

				6
LONRF1	LON peptidase N-terminal domain and ring finger 1	0.88647276	up	0.00119
LOXL2	lysyl oxidase-like 2	1.055334515	up	3.25E-13
LOXL3	lysyl oxidase-like 3	2.123180402	up	0.018378
LPXN	leupaxin	0.946180262	up	0.000377
LRFN3	leucine rich repeat and fibronectin type III domain containing 3	-0.75227594	down	0.000754
LRIG3	leucine-rich repeats and immunoglobulin-like domains 3	0.695200866	up	0.000238
LRP1	low density lipoprotein receptor-related protein 1	0.847511802	up	0.020676
LRP5L	low density lipoprotein receptor-related protein 5-like	0.691630177	up	0.001853
LRP8	low density lipoprotein receptor-related protein 8, apolipoprotein e receptor	-0.65682873	down	0.000148
LRRC24	leucine rich repeat containing 24	1.277591529	up	0.000308
LRRC37B	leucine rich repeat containing 37B	0.9267211	up	4.52E-05
LRRC40	leucine rich repeat containing 40	-0.64536179	down	0.033566
LRRC45	leucine rich repeat containing 45	-0.75511394	down	0.000517
LRRC47	leucine rich repeat containing 47	-1.19421525	down	1.22E-09
LRRC73	leucine rich repeat containing 73	1.420044133	up	9.38E-06
LRRC75B	leucine rich repeat containing 75B	0.71968873	up	0.002394
LRRC8A	leucine rich repeat containing 8 family, member A	0.608028142	up	0.000152
LRRC8E	leucine rich repeat containing 8 family, member E	1.394610067	up	1.98E-07
LRRCC1	leucine rich repeat and coiled-coil centrosomal protein 1	-1.06611766	down	0.040597
LSM12	LSM12 homolog (S. cerevisiae)	-1.59874857	down	1.1E-13
LSR	lipolysis stimulated lipoprotein receptor	1.30705253	up	0.000227
LTB	lymphotoxin beta (TNF superfamily, member 3)	1.352572174	up	0.014063

LTBP1	latent transforming growth factor beta binding protein 1	-0.63687763	down	0.046968
LTN1	listerin E3 ubiquitin protein ligase 1	-1.06650617	down	1.36E-07
LURAP1L	leucine rich adaptor protein 1-like	0.634468795	up	0.000632
LY6E	lymphocyte antigen 6 complex, locus E	0.617548001	up	6.94E-05
LY6K	lymphocyte antigen 6 complex, locus K	1.487182102	up	0.000628
LYPD1	LY6/PLAUR domain containing 1	0.919991374	up	0.008795
LYPD3	LY6/PLAUR domain containing 3	1.074080037	up	0.000165
LYPLA2	lysophospholipase II	0.605228669	up	0.027447
LYRM2	LYR motif containing 2	-1.21370075	down	8.28E-07
LYST	lysosomal trafficking regulator	1.417043192	up	1.77E-05
LZTS3	leucine zipper, putative tumor suppressor family member 3	1.022416534	up	3.62E-08
MAATS1	MYCBP-associated, testis expressed 1	1.288966561	up	0.015148
MAD2L1	MAD2 mitotic arrest deficient-like 1 (yeast)	-1.17594543	down	1.55E-09
MAEA	macrophage erythroblast attacher	-1.16125838	down	1.82E-08
MAFF	v-maf avian musculoaponeurotic fibrosarcoma oncogene homolog F	0.863506285	up	0.02282
MAGOHB	mago-nashi homolog B (Drosophila)	-0.65811205	down	0.025187
MALL	mal, T-cell differentiation protein-like	-2.05277036	down	0.001276
MAML2	mastermind-like 2 (Drosophila)	0.698834823	up	0.005971
MAMLD1	mastermind-like domain containing 1	1.1621308	up	9.48E-11
MAN2B1	mannosidase, alpha, class 2B, member 1	0.613232961	up	0.000128
MANBA	mannosidase, beta A, lysosomal	1.036894042	up	1.6E-07
MANEA	mannosidase, endo-alpha	-1.3833361	down	0.001897
MANSC1	MANSC domain containing 1	-0.77860077	down	0.03066

				4
MAOA	monoamine oxidase A	-0.90875185	down	0.000125
MAP1LC3A	microtubule-associated protein 1 light chain 3 alpha	1.059337436	up	8.11E-06
MAP1LC3B	microtubule-associated protein 1 light chain 3 beta	0.668513673	up	0.004814
MAP1S	microtubule-associated protein 1S	0.794547162	up	9.05E-05
MAP2K2	mitogen-activated protein kinase kinase 2	0.746996766	up	9.65E-06
MAP2K3	mitogen-activated protein kinase kinase 3	1.024280537	up	0.00016
MAP2K6	mitogen-activated protein kinase kinase 6	-1.63748927	down	0.000884
MAP3K1	mitogen-activated protein kinase kinase kinase 1, E3 ubiquitin protein ligase	0.856313758	up	2.88E-06
MAP3K12	mitogen-activated protein kinase kinase kinase 12	0.787991834	up	1.92E-06
MAP3K4	mitogen-activated protein kinase kinase kinase 4	-0.70910164	down	0.000619
MAP3K6	mitogen-activated protein kinase kinase kinase 6	-0.904269	down	0.000599
MAP6	microtubule-associated protein 6	1.320739254	up	0.002615
MAP7	microtubule-associated protein 7	-0.84587129	down	7.28E-06
MAP9	microtubule-associated protein 9	-1.18554591	down	0.016315
MAPK1	mitogen-activated protein kinase 1	-0.59561382	down	0.000513
MAPK15	mitogen-activated protein kinase 15	2.543379759	up	0.019657
MAPK8IP1	mitogen-activated protein kinase 8 interacting protein 1	0.967969527	up	1.1E-06
MAPK8IP2	mitogen-activated protein kinase 8 interacting protein 2	1.078858718	up	3.97E-05
MAPK8IP3	mitogen-activated protein kinase 8 interacting protein 3	1.158473793	up	0.000349
MAPK9	mitogen-activated protein kinase 9	-0.71436609	down	5.05E-05
MAPKAP1	mitogen-activated protein kinase associated protein 1	-0.89374544	down	4.09E-07
MARCH3	membrane-associated ring finger (C3HC4) 3, E3 ubiquitin protein ligase	1.604761018	up	6.55E-09

MARCH4	membrane-associated ring finger (C3HC4) 4, E3 ubiquitin protein ligase	0.917985359	up	1.91E-05
MARCH9	membrane-associated ring finger (C3HC4) 9	0.661759493	up	0.000894
MARK4	MAP/microtubule affinity-regulating kinase 4	0.796719881	up	1.15E-05
MARVELD2	MARVEL domain containing 2	-0.84735129	down	0.013122
MAST1	microtubule associated serine/threonine kinase 1	1.243461736	up	0.000307
MATN2	matrilin 2	-0.65773102	down	0.000917
MAU2	MAU2 sister chromatid cohesion factor	0.635747301	up	0.000324
MB	myoglobin	-0.72207684	down	0.002843
MB21D2	Mab-21 domain containing 2	0.746056825	up	0.001927
MBD3	methyl-CpG binding domain protein 3	-1.73983421	down	5.45E-10
MBLAC2	metallo-beta-lactamase domain containing 2	-1.13524852	down	0.006244
MCCC2	methylcrotonoyl-CoA carboxylase 2 (beta)	-0.83463892	down	4.92E-07
MCL1	myeloid cell leukemia 1	0.854765001	up	7.73E-08
MCM10	minichromosome maintenance complex component 10	-1.91922717	down	2.55E-24
MCM2	minichromosome maintenance complex component 2	-1.08597524	down	3.63E-11
MCM3	minichromosome maintenance complex component 3	-2.49144613	down	8.5E-24
MCM4	minichromosome maintenance complex component 4	-1.82093487	down	1.3E-22
MCM5	minichromosome maintenance complex component 5	-1.40454593	down	1.97E-18
MCM6	minichromosome maintenance complex component 6	-1.62100417	down	5.29E-26
MCM7	minichromosome maintenance complex component 7	-1.13093119	down	4.33E-12
MCM8	minichromosome maintenance complex component 8	-1.62961159	down	2.69E-21
MCM9	minichromosome maintenance complex component 9	-1.79666835	down	6.57E-07
MCTP1	multiple C2 domains, transmembrane 1	1.305134625	up	5.67E-0

				6
MDC1	mediator of DNA-damage checkpoint 1	-0.79683915	down	5.01E-05
MDFIC	MyoD family inhibitor domain containing	-0.6834988	down	0.005721
MDK	midkine (neurite growth-promoting factor 2)	0.618890962	up	0.00467
MDM1	Mdm1 nuclear protein homolog (mouse)	-0.64821474	down	0.015491
ME1	malic enzyme 1, NADP(+)-dependent, cytosolic	-0.65376675	down	0.000119
MED10	mediator complex subunit 10	0.598816879	up	0.001501
MED15	mediator complex subunit 15	1.103096404	up	2.01E-12
MED26	mediator complex subunit 26	1.131352069	up	0.000401
MEF2D	myocyte enhancer factor 2D	0.615155313	up	0.000971
MEFV	Mediterranean fever	2.897235086	up	0.000407
MEGF6	multiple EGF-like-domains 6	0.592055723	up	0.002025
MEGF8	multiple EGF-like-domains 8	-0.99963021	down	0.012746
MEGF9	multiple EGF-like-domains 9	-1.27605128	down	8.79E-14
MEIS2	Meis homeobox 2	-0.74738221	down	0.004256
MELK	maternal embryonic leucine zipper kinase	-1.16957313	down	4.67E-13
MESDC1	mesoderm development candidate 1	0.64271786	up	0.001479
METRNL	meteorin, glial cell differentiation regulator-like	0.82641128	up	0.000274
METTL15	methyltransferase like 15	-1.0186787	down	0.015193
METTL23	methyltransferase like 23	0.590270581	up	0.00094
METTL2A	methyltransferase like 2A	-0.74054901	down	2.27E-05
METTL3	methyltransferase like 3	-0.63241476	down	0.000379
METTL4	methyltransferase like 4	-0.7318008	down	0.025298

METTL7A	methyltransferase like 7A	-20	down	0.01293
METTL8	methyltransferase like 8	-0.6169652	down	0.043945
MFAP3	microfibrillar-associated protein 3	-1.24085617	down	3.82E-11
MFAP3L	microfibrillar-associated protein 3-like	-0.72623543	down	0.008937
MFGE8	milk fat globule-EGF factor 8 protein	0.691508814	up	2.17E-05
MGAM	maltase-glucoamylase (alpha-glucosidase)	-2.12221478	down	5.67E-06
MIA3	melanoma inhibitory activity family, member 3	0.866710327	up	3.58E-06
MICA	MHC class I polypeptide-related sequence A	0.773643224	up	2.39E-05
MICAL1	microtubule associated monooxygenase, calponin and LIM domain containing 1	0.932709989	up	1.95E-07
MICAL2	microtubule associated monooxygenase, calponin and LIM domain containing 2	0.673987719	up	0.037976
MICALCL	MICAL C-terminal like	1.750924	up	0.000291
MICB	MHC class I polypeptide-related sequence B	0.926382978	up	4.8E-08
MIER2	mesoderm induction early response 1, family member 2	0.666358729	up	0.000397
MIIP	migration and invasion inhibitory protein	0.640817874	up	0.020707
MIOX	myo-inositol oxygenase	1.380803805	up	0.000809
MIPEP	mitochondrial intermediate peptidase	-2.12700508	down	5.57E-12
MIS18A	MIS18 kinetochore protein A	-0.66295445	down	0.006116
MIS18BP1	MIS18 binding protein 1	-1.28960261	down	1.78E-06
MKI67	marker of proliferation Ki-67	-1.97069611	down	3.47E-19
MKS1	Meckel syndrome, type 1	-1.43276777	down	4.75E-13
MLEC	malectin	-1.41905534	down	6.03E-09
MLH1	mutL homolog 1	-1.31767917	down	2.31E-06
MLLT11	myeloid/lymphoid or mixed-lineage leukemia (trithorax homolog,	-0.71513827	down	4.02E-0

	Drosophila); translocated to, 11			5
MLYCD	malonyl-CoA decarboxylase	-1.00280473	down	6.99E-05
MMD	monocyte to macrophage differentiation-associated	-0.61781687	down	0.00208
MMP10	matrix metallopeptidase 10 (stromelysin 2)	20	up	0.004807
MMP13	matrix metallopeptidase 13 (collagenase 3)	3.524869611	up	0.008819
MMP17	matrix metallopeptidase 17 (membrane-inserted)	0.608732379	up	0.029315
MMP19	matrix metallopeptidase 19	1.683008846	up	0.010653
MMP24	matrix metallopeptidase 24 (membrane-inserted)	-1.3837718	down	8.15E-13
MMS22L	MMS22-like, DNA repair protein	-0.92177899	down	6.09E-05
MNS1	meiosis-specific nuclear structural 1	-1.68548727	down	0.000556
MOB1A	MOB kinase activator 1A	-0.65290738	down	0.000104
MOB1B	MOB kinase activator 1B	-1.17231325	down	3.66E-05
MOB4	MOB family member 4, phocein	-0.61194082	down	0.015193
MORC3	MORC family CW-type zinc finger 3	0.727201032	up	0.034659
MPC1	mitochondrial pyruvate carrier 1	1.035154635	up	0.001422
MPHOSPH6	M-phase phosphoprotein 6	-1.02440663	down	0.000168
MPHOSPH9	M-phase phosphoprotein 9	-1.0979896	down	8.94E-08
MPP1	membrane protein, palmitoylated 1, 55kDa	0.942146489	up	1.84E-05
MPZL3	myelin protein zero-like 3	3.343979837	up	6.48E-05
MRAP2	melanocortin 2 receptor accessory protein 2	-1.85722466	down	0.002073
MRAS	muscle RAS oncogene homolog	-0.59361685	down	0.014073
MREG	melanoregulin	-0.70722736	down	0.019491
MRPL16	mitochondrial ribosomal protein L16	-0.68311188	down	0.00015

				6
MRPL43	mitochondrial ribosomal protein L43	-0.67221538	down	8.26E-05
MRPL51	mitochondrial ribosomal protein L51	-0.90854145	down	6.02E-08
MRS2	MRS2 magnesium transporter	-1.39119595	down	8.5E-12
MRTO4	mRNA turnover 4 homolog (S. cerevisiae)	-0.64911381	down	0.001696
MSANTD3	Myb/SANT-like DNA-binding domain containing 3	-1.48583111	down	4.24E-15
MSANTD3-TMEFF1	MSANTD3-TMEFF1 readthrough	-1.0636857	down	1.6E-06
MSMO1	methylsterol monooxygenase 1	-0.81685722	down	0.000185
MSRB1	methionine sulfoxide reductase B1	-0.71697638	down	4.5E-05
MT1F	metallothionein 1F	0.733193106	up	0.004451
MT1X	metallothionein 1X	1.262041371	up	1.66E-05
MT2A	metallothionein 2A	0.789483127	up	0.011135
MTAP	methylthioadenosine phosphorylase	1.276393267	up	0.002517
MTBP	MDM2 binding protein	-1.40606735	down	9.86E-05
MTF1	metal-regulatory transcription factor 1	0.585495649	up	0.004482
MTFR2	mitochondrial fission regulator 2	-0.8289618	down	0.003979
MTMR11	myotubularin related protein 11	0.614353437	up	0.006756
MTMR14	myotubularin related protein 14	0.62663083	up	0.000294
MTOR	mechanistic target of rapamycin (serine/threonine kinase)	-0.7974088	down	1.47E-05
MTRNR2L8	MT-RNR2-like 8	-1.46667229	down	0.034659
MTSS1	metastasis suppressor 1	1.863486269	up	1.25E-10
MTUS1	microtubule associated tumor suppressor 1	-1.23232143	down	1.27E-09
MTX3	metaxin 3	-1.49396482	down	1.23E-10

MUC3A	mucin 3A, cell surface associated	0.969320322	up	9.54E-05
MUC5AC	mucin 5AC, oligomeric mucus/gel-forming	-1.3472053	down	0.00083
MUM1	melanoma associated antigen (mutated) 1	-0.63010326	down	0.004527
MX1	MX dynamin-like GTPase 1	0.898349759	up	0.030301
MX2	MX dynamin-like GTPase 2	1.464395984	up	0.021045
MXD1	MAX dimerization protein 1	1.48759928	up	0.000274
MXD3	MAX dimerization protein 3	-0.70795417	down	0.002695
MYADM	myeloid-associated differentiation marker	0.625478597	up	0.004614
MYBL1	v-myb avian myeloblastosis viral oncogene homolog-like 1	-0.68125629	down	0.008869
MYBL2	v-myb avian myeloblastosis viral oncogene homolog-like 2	-1.12268013	down	1.44E-09
MYEOV	myeloma overexpressed	0.949891004	up	7.09E-10
MYL9	myosin, light chain 9, regulatory	3.548648901	up	0.001005
MYO5B	myosin VB	0.773684166	up	0.000123
MYO5C	myosin VC	-1.19737462	down	1.59E-07
MYO9A	myosin IXA	-0.91669826	down	2.02E-06
MYRF	myelin regulatory factor	-1.5528865	down	0.007872
N4BP3	NEDD4 binding protein 3	1.441181869	up	2.76E-05
NAAA	N-acylethanolamine acid amidase	-0.64112262	down	0.000766
NACAD	NAC alpha domain containing	2.279699502	up	0.002635
NAGK	N-acetylglucosamine kinase	0.717344126	up	5.8E-05
NAP1L4	nucleosome assembly protein 1-like 4	-0.78446178	down	1.14E-06
NAPA	N-ethylmaleimide-sensitive factor attachment protein, alpha	0.601353576	up	0.013035
NAPB	N-ethylmaleimide-sensitive factor attachment protein, beta	0.652844542	up	0.03042

				1
NARS2	asparaginyl-tRNA synthetase 2, mitochondrial (putative)	-0.58973949	down	0.01015 7
NASP	nuclear autoantigenic sperm protein (histone-binding)	-1.18696263	down	8.47E-1 4
NAT8L	N-acetyltransferase 8-like (GCN5-related, putative)	-0.8393683	down	0.00161 2
NCALD	neurocalcin delta	-1.16674071	down	2.63E-0 7
NCAPD2	non-SMC condensin I complex, subunit D2	-1.41557607	down	1.38E-0 9
NCAPD3	non-SMC condensin II complex, subunit D3	-0.94482647	down	4E-07
NCAPG	non-SMC condensin I complex, subunit G	-0.73236108	down	0.00260 1
NCAPG2	non-SMC condensin II complex, subunit G2	-0.98157314	down	6.52E-1 0
NCAPH	non-SMC condensin I complex, subunit H	-1.00634789	down	4.85E-1 0
NCCRP1	non-specific cytotoxic cell receptor protein 1 homolog (zebrafish)	1.472290602	up	0.00634
NCEH1	neutral cholesterol ester hydrolase 1	-0.66326689	down	0.00010 8
NCK1	NCK adaptor protein 1	1.356739453	up	0.02085 6
NCKAP1	NCK-associated protein 1	-0.97900508	down	1.05E-1 0
NCL	nucleolin	-0.61183568	down	0.00417 4
NDC1	NDC1 transmembrane nucleoporin	-1.72417338	down	6.91E-2 3
NDC80	NDC80 kinetochore complex component	-0.92875054	down	0.00352 1
NDFIP2	Nedd4 family interacting protein 2	-1.25335733	down	7.34E-1 2
NDRG1	N-myc downstream regulated 1	1.081886288	up	0.00100 4
NDUFB11	NADH dehydrogenase (ubiquinone) 1 beta subcomplex, 11, 17.3kDa	-1.92339626	down	1.41E-3 4
NDUFC2	NADH dehydrogenase (ubiquinone) 1, subcomplex unknown, 2, 14.5kDa	-1.17095823	down	1.03E-0 5
NDUFS6	NADH dehydrogenase (ubiquinone) Fe-S protein 6, 13kDa (NADH-coenzyme Q reductase)	-0.63791832	down	0.00017 9
NEBL	nebulette	-0.60315655	down	0.0038
NECAB3	N-terminal EF-hand calcium binding protein 3	0.737491871	up	0.00016

				8
NECAP1	NECAP endocytosis associated 1	1.003211698	up	6.58E-08
NEDD1	neural precursor cell expressed, developmentally down-regulated 1	-1.2934877	down	1.49E-11
NEFL	neurofilament, light polypeptide	1.578270988	up	2.94E-06
NEFM	neurofilament, medium polypeptide	2.860584049	up	0.012574
NEIL3	nei endonuclease VIII-like 3 (E. coli)	-0.72651783	down	0.004415
NEK8	NIMA-related kinase 8	0.683022496	up	0.004398
NELFCD	negative elongation factor complex member C/D	-0.61894925	down	0.006214
NEMF	nuclear export mediator factor	-1.08816092	down	7.4E-08
NEMP1	transmembrane protein 194A	-0.94021836	down	1.17E-07
NEMP2	transmembrane protein 194B	-1.08916899	down	4.24E-05
NEU1	sialidase 1 (lysosomal sialidase)	0.789681278	up	1.48E-06
NEU3	sialidase 3 (membrane sialidase)	-0.78660375	down	0.008254
NEURL1	neuralized E3 ubiquitin protein ligase 1	2.050465901	up	9.88E-05
NEURL3	neuralized E3 ubiquitin protein ligase 3	3.239872547	up	3.14E-17
NFE2	nuclear factor, erythroid 2	-1.06610389	down	0.016315
NFIL3	nuclear factor, interleukin 3 regulated	1.131916432	up	1.4E-07
NFKB2	nuclear factor of kappa light polypeptide gene enhancer in B-cells 2 (p49/p100)	1.442281875	up	3.71E-11
NFKBIA	nuclear factor of kappa light polypeptide gene enhancer in B-cells inhibitor, alpha	0.960172842	up	0.00737
NFKBIE	nuclear factor of kappa light polypeptide gene enhancer in B-cells inhibitor, epsilon	0.784970849	up	0.030301
NFKBIZ	nuclear factor of kappa light polypeptide gene enhancer in B-cells inhibitor, zeta	1.483505016	up	0.00015
NFX1	nuclear transcription factor, X-box binding 1	0.610893271	up	0.000714
NGEF	neuronal guanine nucleotide exchange factor	0.951994521	up	4.35E-09

NGF	nerve growth factor (beta polypeptide)	1.983611654	up	0.003274
NGFR	nerve growth factor receptor	2.087887657	up	2.6E-05
NID2	nidogen 2 (osteonidogen)	0.647153547	up	0.000365
NIF3L1	NIF3 NGG1 interacting factor 3-like 1 (S. cerevisiae)	-0.66204033	down	0.000848
NIFK	nucleolar protein interacting with the FHA domain of MKI67	0.765080385	up	3.07E-05
NINJ1	ninjurin 1	0.595094417	up	0.00833
NIPA1	non imprinted in Prader-Willi/Angelman syndrome 1	-0.70728032	down	3.62E-05
NIPAL1	NIPA-like domain containing 1	-1.55994286	down	0.000924
NIPAL4	NIPA-like domain containing 4	1.602121961	up	6.99E-19
NIPSNAP3A	nipsnap homolog 3A (C. elegans)	-1.27928219	down	1.45E-07
NKD2	naked cuticle homolog 2 (Drosophila)	0.706953647	up	0.002064
NKIRAS2	NFKB inhibitor interacting Ras-like 2	0.70010104	up	8.57E-06
NKX2-8	NK2 homeobox 8	0.8943332	up	0.023665
NKX3-1	NK3 homeobox 1	1.113295714	up	3.06E-06
NLE1	notchless homolog 1 (Drosophila)	0.866762387	up	7.7E-08
NLRP2	NLR family, pyrin domain containing 2	-0.79132361	down	0.014664
NMD3	NMD3 ribosome export adaptor	0.61950948	up	0.001159
NME1	NME/NM23 nucleoside diphosphate kinase 1	-0.78074329	down	4.94E-07
NMNAT2	nicotinamide nucleotide adenylyltransferase 2	1.270043955	up	0.015715
NNMT	nicotinamide N-methyltransferase	-2.26986881	down	6.43E-15
NOB1	NIN1/RPN12 binding protein 1 homolog (S. cerevisiae)	1.0432292	up	3.47E-12
NOC3L	nucleolar complex associated 3 homolog (S. cerevisiae)	-0.84786643	down	4.65E-05
NOD2	nucleotide-binding oligomerization domain containing 2	1.263777855	up	0.013371

NOL12	nucleolar protein 12	-0.93606459	down	0.000203
NOP58	NOP58 ribonucleoprotein	-0.7307091	down	0.016393
NOSTRIN	nitric oxide synthase trafficking	0.769972227	up	0.012158
NOTCH3	notch 3	-0.96276767	down	2.61E-09
NOTUM	notum pectinacetylesterase homolog (Drosophila)	1.381488868	up	0.008126
NOV	nephroblastoma overexpressed	1.117962165	up	0.022764
NOVA2	neuro-oncological ventral antigen 2	1.297355593	up	7.99E-11
NPAS1	neuronal PAS domain protein 1	1.030867999	up	0.000104
NPAT	nuclear protein, ataxia-telangiectasia locus	-0.71435948	down	0.00719
NPC1	Niemann-Pick disease, type C1	1.239114702	up	1.17E-05
NPC2	Niemann-Pick disease, type C2	0.600703232	up	0.011159
NPHS1	nephrosis 1, congenital, Finnish type (nephrin)	1.122089277	up	1.61E-07
NPIPA8	nuclear pore complex interacting protein family, member A8	1.338830162	up	0.018059
NPIPB6	nuclear pore complex interacting protein family, member B6	1.217176559	up	0.021144
NPLOC4	nuclear protein localization 4 homolog (S. cerevisiae)	-0.72024691	down	4.99E-06
NPTX1	neuronal pentraxin I	0.616430168	up	0.000888
NPY4R	neuropeptide Y receptor Y4	-1.3196111	down	1.57E-12
NR2F2	nuclear receptor subfamily 2, group F, member 2	-0.74693465	down	0.009127
NR5A2	nuclear receptor subfamily 5, group A, member 2	0.828362018	up	4.22E-07
NRARP	NOTCH-regulated ankyrin repeat protein	-0.79209668	down	0.024594
NRDE2	NRDE-2, necessary for RNA interference, domain containing	-0.70910658	down	0.001955
NREP	neuronal regeneration related protein	-0.64634691	down	0.014713

NRGN	neurogranin (protein kinase C substrate, RC3)	-0.9009694	down	0.015728
NRIP3	nuclear receptor interacting protein 3	1.064887953	up	0.000273
NRXN2	neurexin 2	2.215523521	up	0.000714
NSFL1C	NSFL1 (p97) cofactor (p47)	0.626469753	up	0.000128
NSUN2	NOP2/Sun RNA methyltransferase family, member 2	-0.61501506	down	0.000298
NT5C3A	5'-nucleotidase, cytosolic IIIA	1.070945727	up	1.69E-06
NT5DC2	5'-nucleotidase domain containing 2	-1.23001859	down	2.97E-12
NT5E	5'-nucleotidase, ecto (CD73)	0.62349544	up	0.002096
NUCKS1	nuclear casein kinase and cyclin-dependent kinase substrate 1	-0.64669282	down	5.62E-05
NUDCD1	NudC domain containing 1	-0.88680332	down	5.04E-07
NUDT1	nudix (nucleoside diphosphate linked moiety X)-type motif 1	-0.87198436	down	6E-07
NUDT15	nudix (nucleoside diphosphate linked moiety X)-type motif 15	-0.72681119	down	0.002843
NUDT16	nudix (nucleoside diphosphate linked moiety X)-type motif 16	-0.90404585	down	1.03E-05
NUDT17	nudix (nucleoside diphosphate linked moiety X)-type motif 17	0.813590456	up	0.008337
NUDT19	nudix (nucleoside diphosphate linked moiety X)-type motif 19	-0.90111526	down	1.66E-05
NUDT3	nudix (nucleoside diphosphate linked moiety X)-type motif 3	-1.82405372	down	4.96E-05
NUDT7	nudix (nucleoside diphosphate linked moiety X)-type motif 7	-0.88535593	down	0.000241
NUF2	NUF2, NDC80 kinetochore complex component	-0.98615631	down	2.45E-06
NUP107	nucleoporin 107kDa	-0.81460212	down	0.000156
NUP155	nucleoporin 155kDa	-0.69134055	down	0.000485
NUP205	nucleoporin 205kDa	-0.65505067	down	5.62E-05
NUP58	nucleoporin like 1	-1.01509323	down	2.13E-08

NUP62CL	nucleoporin 62kDa C-terminal like	-0.85421094	down	0.006122
NUP88	nucleoporin 88kDa	-0.73536886	down	1.18E-05
NUSAP1	nucleolar and spindle associated protein 1	-1.32143352	down	9E-18
NXPE3	neurexophilin and PC-esterase domain family, member 3	-1.29061168	down	9.55E-12
NXPH3	neurexophilin 3	1.12403831	up	0.045932
NXPH4	neurexophilin 4	0.720440868	up	0.012295
OAF	OAF homolog (Drosophila)	0.676421703	up	0.000532
OAS1	2'-5'-oligoadenylate synthetase 1, 40/46kDa	0.634821442	up	7E-05
OAS2	2'-5'-oligoadenylate synthetase 2, 69/71kDa	1.147251716	up	0.015899
OAS3	2'-5'-oligoadenylate synthetase 3, 100kDa	-1.22016471	down	3.26E-11
OASL	2'-5'-oligoadenylate synthetase-like	1.539478563	up	3.86E-10
ODF3B	outer dense fiber of sperm tails 3B	1.16821594	up	0.000165
OGDHL	oxoglutarate dehydrogenase-like	2.510404038	up	2.69E-05
OGFOD3	2-oxoglutarate and iron-dependent oxygenase domain containing 3	-0.84705628	down	2.43E-05
OGFRL1	opioid growth factor receptor-like 1	0.770535805	up	9.46E-05
OIP5	Opa interacting protein 5	-2.47567915	down	1.61E-11
OLR1	oxidized low density lipoprotein (lectin-like) receptor 1	0.603286056	up	0.047136
OPA1	optic atrophy 1 (autosomal dominant)	-0.58971835	down	0.043827
OPN3	opsin 3	-0.93513909	down	4.46E-09
OPTN	optineurin	1.296309619	up	1.56E-08
ORAI1	ORAI calcium release-activated calcium modulator 1	0.784124368	up	2.27E-06
ORC1	origin recognition complex, subunit 1	-1.2465629	down	1E-10
ORC2	origin recognition complex, subunit 2	-0.71816926	down	0.000172

ORC6	origin recognition complex, subunit 6	-0.74614808	down	0.000874
ORMDL3	ORMDL sphingolipid biosynthesis regulator 3	-0.77674502	down	9.93E-06
OSBPL1A	oxysterol binding protein-like 1A	-0.69586542	down	0.004027
OSBPL3	oxysterol binding protein-like 3	-0.82100739	down	9.07E-06
OSBPL5	oxysterol binding protein-like 5	0.848527908	up	1.76E-06
OSBPL6	oxysterol binding protein-like 6	0.914972309	up	0.005262
OSBPL7	oxysterol binding protein-like 7	0.638523212	up	0.001796
OSER1	oxidative stress responsive serine-rich 1	1.231937044	up	9.53E-10
OSGEP	O-sialoglycoprotein endopeptidase	-0.62199371	down	0.002922
OTUB2	OTU deubiquitinase, ubiquitin aldehyde binding 2	0.9736958	up	3.12E-07
OTUD1	OTU deubiquitinase 1	0.63345489	up	0.034119
OTUD4	OTU deubiquitinase 4	-1.18342726	down	1.68E-10
OTUD6B	OTU domain containing 6B	-0.8918106	down	0.002921
OXTR	oxytocin receptor	0.948939583	up	0.000746
P2RX4	purinergic receptor P2X, ligand-gated ion channel, 4	1.070430761	up	1.11E-10
P4HA2	prolyl 4-hydroxylase, alpha polypeptide II	0.941909503	up	1.12E-09
PADI2	peptidyl arginine deiminase, type II	0.906701268	up	1.03E-05
PADI3	peptidyl arginine deiminase, type III	0.6995624	up	5.35E-05
PAFAH1B2	platelet-activating factor acetylhydrolase 1b, catalytic subunit 2 (30kDa)	-1.59415818	down	1.02E-21
PAG1	phosphoprotein membrane anchor with glycosphingolipid microdomains 1	4.204955226	up	6.09E-05
PAM	peptidylglycine alpha-amidating monooxygenase	0.690053502	up	4.75E-05
PANK1	pantothenate kinase 1	-1.56726292	down	0.005125

PANK3	pantothenate kinase 3	-0.79686146	down	0.00065
PAPSS2	3'-phosphoadenosine 5'-phosphosulfate synthase 2	-0.92250757	down	4.72E-05
PAQR5	progestin and adipoQ receptor family member V	0.723272759	up	0.002354
PAQR9	progestin and adipoQ receptor family member IX	-1.168906	down	0.017447
PARD6B	par-6 family cell polarity regulator beta	0.957433966	up	0.022405
PARG	poly (ADP-ribose) glycohydrolase	-0.96072541	down	6.52E-06
PARP12	poly (ADP-ribose) polymerase family, member 12	0.74277696	up	0.026293
PARP3	poly (ADP-ribose) polymerase family, member 3	0.808262298	up	4.27E-05
PARP6	poly (ADP-ribose) polymerase family, member 6	0.801475621	up	5.66E-07
PARPBP	PARP1 binding protein	-1.46597924	down	1.23E-06
PARVA	parvin, alpha	-1.15008287	down	5.46E-11
PASK	PAS domain containing serine/threonine kinase	-1.23472176	down	1.57E-11
PAX6	paired box 6	1.439699693	up	6.12E-06
PAX7	paired box 7	-1.085474	down	0.014847
PBK	PDZ binding kinase	-1.61393311	down	8.82E-13
PBX1	pre-B-cell leukemia homeobox 1	-0.9159371	down	0.001667
PBX4	pre-B-cell leukemia homeobox 4	1.149468964	up	0.034831
PBXIP1	pre-B-cell leukemia homeobox interacting protein 1	0.614372868	up	0.000111
PC	pyruvate carboxylase	0.696447673	up	2.54E-05
PCBP4	poly(rC) binding protein 4	0.680269083	up	0.000118
PCDH1	protocadherin 1	1.584693575	up	9.01E-17
PCDH10	protocadherin 10	2.448541597	up	0.047779

PCDHA1	protocadherin alpha 1	2.465971752	up	0.017251
PCDHAC2	protocadherin alpha subfamily C, 2	-0.62909663	down	0.022021
PCDHB5	protocadherin beta 5	-1.11413387	down	0.026973
PCGF1	polycomb group ring finger 1	1.003022698	up	1.46E-07
PCGF3	polycomb group ring finger 3	-0.75543459	down	0.011053
PCGF5	polycomb group ring finger 5	-1.0682312	down	1.9E-09
PCGF6	polycomb group ring finger 6	-1.38653323	down	0.000951
PCLO	piccolo presynaptic cytomatrix protein	-0.87109489	down	0.007392
PCM1	pericentriolar material 1	-0.61522544	down	0.000243
PCNA	proliferating cell nuclear antigen	-1.39291555	down	6.94E-20
PCNT	pericentrin	-0.78759829	down	0.021197
PCSK6	proprotein convertase subtilisin/kexin type 6	-1.12239938	down	0.008256
PCSK7	proprotein convertase subtilisin/kexin type 7	-0.66510104	down	0.003003
PCSK9	proprotein convertase subtilisin/kexin type 9	-1.02382732	down	0.005511
PCTP	phosphatidylcholine transfer protein	0.66983641	up	0.000207
PDCD4	programmed cell death 4 (neoplastic transformation inhibitor)	-1.31927867	down	1.28E-10
PDCL3	phosducin-like 3	-0.74993091	down	0.005105
PDE2A	phosphodiesterase 2A, cGMP-stimulated	1.145453674	up	0.029268
PDGFRB	platelet-derived growth factor receptor, beta polypeptide	3.337242961	up	0.007089
PDIA4	protein disulfide isomerase family A, member 4	-1.15327787	down	6.24E-15
PDIA5	protein disulfide isomerase family A, member 5	-0.94072363	down	1.08E-08
PDLIM2	PDZ and LIM domain 2 (mystique)	0.847358377	up	1.4E-05
PDLIM7	PDZ and LIM domain 7 (enigma)	0.971545321	up	3.32E-0

				9
PDP1	pyruvate dehyrogenase phosphatase catalytic subunit 1	1.052670899	up	3.2E-09
PDPK1	3-phosphoinositide dependent protein kinase 1	-0.72693092	down	0.00013<br>3
PDRG1	p53 and DNA-damage regulated 1	0.644344969	up	0.00203<br>1
PDS5A	PDS5, regulator of cohesion maintenance, homolog A (S. cerevisiae)	-0.71772759	down	1.03E-0<br>5
PDZD4	PDZ domain containing 4	1.276326563	up	2.32E-0<br>7
PDZD7	PDZ domain containing 7	0.699130676	up	0.04910<br>4
PEA15	phosphoprotein enriched in astrocytes 15	1.260181386	up	5.08E-1<br>7
PECR	peroxisomal trans-2-enoyl-CoA reductase	-0.83286837	down	3.33E-0<br>5
PEG10	paternally expressed 10	-1.1592691	down	3.9E-08
PELI3	pellino E3 ubiquitin protein ligase family member 3	0.942059407	up	2.68E-0<br>5
PER3	period circadian clock 3	-0.93162958	down	0.00068<br>9
PEX11B	peroxisomal biogenesis factor 11 beta	-0.5994932	down	0.01347<br>1
PEX12	peroxisomal biogenesis factor 12	-0.64513296	down	0.01748<br>1
PEX16	peroxisomal biogenesis factor 16	0.75336903	up	0.00261<br>5
PEX19	peroxisomal biogenesis factor 19	-0.70741019	down	5.06E-0<br>5
PFAS	phosphoribosylformylglycinamidine synthase	-1.22045011	down	6.9E-07
PFKFB2	6-phosphofructo-2-kinase/fructose-2,6-biphosphatase 2	-1.27119022	down	2.19E-0<br>8
PGAM1	phosphoglycerate mutase 1 (brain)	-0.8376971	down	7.85E-0<br>5
PGAM4	phosphoglycerate mutase family member 4	-1.03005601	down	0.00047<br>6
PGD	phosphogluconate dehydrogenase	-0.8729457	down	2.05E-0<br>9
PGF	placental growth factor	0.852876641	up	0.00639<br>1
PGGT1B	protein geranylgeranyltransferase type I, beta subunit	-1.02793004	down	5.25E-0<br>6
PHACTR4	phosphatase and actin regulator 4	-0.9128154	down	4.55E-0

				6
PHC2	polyhomeotic homolog 2 (Drosophila)	0.819775581	up	1.59E-07
PHF11	PHD finger protein 11	0.876371487	up	0.000593
PHF14	PHD finger protein 14	-0.68625518	down	0.007808
PHF19	PHD finger protein 19	-0.7002355	down	9.54E-05
PHGDH	phosphoglycerate dehydrogenase	-1.36973005	down	4.15E-09
PHKG1	phosphorylase kinase, gamma 1 (muscle)	1.595271551	up	0.025931
PI3	peptidase inhibitor 3, skin-derived	1.491019838	up	0.001151
PI4K2A	phosphatidylinositol 4-kinase type 2 alpha	0.779520471	up	8.7E-07
PIAS4	protein inhibitor of activated STAT, 4	0.635585342	up	0.001008
PID1	phosphotyrosine interaction domain containing 1	1.021424416	up	0.002947
PIF1	PIF1 5'-to-3' DNA helicase	-1.39020694	down	3E-08
PIFO	primary cilia formation	1.840181813	up	0.000839
PIGH	phosphatidylinositol glycan anchor biosynthesis, class H	1.235920889	up	9.76E-11
PIGK	phosphatidylinositol glycan anchor biosynthesis, class K	-0.85858308	down	0.000566
PIGW	phosphatidylinositol glycan anchor biosynthesis, class W	-1.37443855	down	0.002218
PIK3C2A	phosphatidylinositol-4-phosphate 3-kinase, catalytic subunit type 2 alpha	-0.86337994	down	5.95E-05
PIK3IP1	phosphoinositide-3-kinase interacting protein 1	1.248402367	up	0.001123
PIK3R3	phosphoinositide-3-kinase, regulatory subunit 3 (gamma)	-1.30166985	down	0.002294
PIK3R4	phosphoinositide-3-kinase, regulatory subunit 4	-0.79531827	down	4.65E-05
PIM1	Pim-1 proto-oncogene, serine/threonine kinase	0.845651106	up	0.011767
PIM3	Pim-3 proto-oncogene, serine/threonine kinase	0.908582478	up	0.015155
PIP5K1C	phosphatidylinositol-4-phosphate 5-kinase, type I, gamma	-0.82801448	down	3.7E-05
PKDCC	protein kinase domain containing, cytoplasmic	1.022661028	up	4.07E-1

				0
PKN2	protein kinase N2	-0.8283283	down	9.56E-05
PLA2G15	phospholipase A2, group XV	0.613126229	up	0.000861
PLAGL2	pleiomorphic adenoma gene-like 2	-0.67867533	down	0.000552
PLAT	plasminogen activator, tissue	0.692436301	up	0.000445
PLAUR	plasminogen activator, urokinase receptor	0.758206371	up	0.001513
PLCD1	phospholipase C, delta 1	0.871622676	up	7.4E-07
PLCE1	phospholipase C, epsilon 1	-1.0831162	down	0.002236
PLCH2	phospholipase C, eta 2	1.537048624	up	0.000342
PLD2	phospholipase D2	0.618230261	up	0.000199
PLD3	phospholipase D family, member 3	0.590905188	up	0.000285
PLD6	phospholipase D family, member 6	1.145091536	up	6.07E-05
PLEK2	pleckstrin 2	1.817517604	up	4.99E-22
PLEKHB2	pleckstrin homology domain containing, family B (evectins) member 2	-1.17669164	down	8.67E-15
PLEKHG5	pleckstrin homology domain containing, family G (with RhoGef domain) member 5	1.018402056	up	7.6E-06
PLEKHJ1	pleckstrin homology domain containing, family J member 1	0.735850317	up	0.000324
PLEKHM1	pleckstrin homology domain containing, family M (with RUN domain) member 1	0.998472131	up	3E-10
PLEKHO1	pleckstrin homology domain containing, family O member 1	0.774664543	up	0.001662
PLIN2	perilipin 2	1.131435898	up	1.42E-06
PLK1	polo-like kinase 1	-0.92229534	down	6.24E-09
PLK3	polo-like kinase 3	2.056143166	up	1.44E-09
PLK4	polo-like kinase 4	-1.05253413	down	0.000525
PLLP	plasmolipin	0.831719974	up	0.00062

				1
PLXDC1	plexin domain containing 1	1.479603974	up	0.01593 9
PLXNA3	plexin A3	0.719030212	up	4.28E-0 5
PLXNB3	plexin B3	0.923833316	up	0.00020 7
PMAIP1	phorbol-12-myristate-13-acetate-induced protein 1	0.861936891	up	0.00030 8
PMF1	polyamine-modulated factor 1	-0.70698103	down	0.04736 8
PML	promyelocytic leukemia	1.141845869	up	3.98E-1 3
PMP22	peripheral myelin protein 22	0.700277558	up	7.59E-0 6
PNO1	partner of NOB1 homolog (S. cerevisiae)	-0.63603436	down	0.02143 5
PNRC2	proline-rich nuclear receptor coactivator 2	0.832380428	up	7.18E-0 6
POC1A	POC1 centriolar protein A	-0.58700653	down	0.00814 6
POC1B-GALNT4	POC1B-GALNT4 readthrough	-1.21436481	down	2.52E-0 5
POFUT1	protein O-fucosyltransferase 1	-1.83021768	down	9.65E-3 2
POLA1	polymerase (DNA directed), alpha 1, catalytic subunit	-1.00451071	down	0.00248 6
POLA2	polymerase (DNA directed), alpha 2, accessory subunit	-1.62891187	down	9.81E-1 9
POLE	polymerase (DNA directed), epsilon, catalytic subunit	-0.88605136	down	6.19E-0 8
POLE2	polymerase (DNA directed), epsilon 2, accessory subunit	-1.50719456	down	0.00014 2
POLE3	polymerase (DNA directed), epsilon 3, accessory subunit	-0.63466733	down	0.00034 1
POLL	polymerase (DNA directed), lambda	0.771295202	up	4.09E-0 6
POLM	polymerase (DNA directed), mu	0.709355578	up	0.00812 6
POLQ	polymerase (DNA directed), theta	-0.98347829	down	0.00021 4
POLR1A	polymerase (RNA) I polypeptide A, 194kDa	-0.88987236	down	0.02004 1

POLR1D	polymerase (RNA) I polypeptide D, 16kDa	0.630156594	up	0.000146
POLR2K	polymerase (RNA) II (DNA directed) polypeptide K, 7.0kDa	-0.71410579	down	0.002573
POLR3D	polymerase (RNA) III (DNA directed) polypeptide D, 44kDa	0.786155855	up	3.11E-06
POLR3G	polymerase (RNA) III (DNA directed) polypeptide G (32kD)	-1.62386532	down	0.000257
POLR3H	polymerase (RNA) III (DNA directed) polypeptide H (22.9kD)	-0.68617518	down	0.018321
POLR3K	polymerase (RNA) III (DNA directed) polypeptide K, 12.3 kDa	-0.7149903	down	0.001853
POM121	POM121 transmembrane nucleoporin	-0.81915361	down	1.64E-05
POM121C	POM121 transmembrane nucleoporin C	-0.91808548	down	8.08E-07
POMZP3	POM121 and ZP3 fusion	-0.70195567	down	0.044299
POP1	processing of precursor 1, ribonuclease P/MRP subunit (S. cerevisiae)	-0.80030799	down	0.013433
POP5	processing of precursor 5, ribonuclease P/MRP subunit (S. cerevisiae)	-0.74309049	down	0.001146
POU2F2	POU class 2 homeobox 2	1.379910063	up	0.003068
PPARG	peroxisome proliferator-activated receptor gamma	0.595138354	up	0.000549
PPAT	phosphoribosyl pyrophosphate amidotransferase	-0.87170668	down	9.37E-06
PPFIA3	protein tyrosine phosphatase, receptor type, f polypeptide (PTPRF), interacting protein (liprin), alpha 3	0.798561324	up	6.21E-05
PPHLN1	periphilin 1	-0.81540069	down	7.37E-06
PPIF	peptidylprolyl isomerase F	0.950660297	up	1.28E-09
PPIH	peptidylprolyl isomerase H (cyclophilin H)	-0.64469469	down	0.001185
PPIL1	peptidylprolyl isomerase (cyclophilin)-like 1	-0.60601119	down	0.001374
PPM1D	protein phosphatase, Mg2+/Mn2+ dependent, 1D	0.764926023	up	0.001867
PPM1J	protein phosphatase, Mg2+/Mn2+ dependent, 1J	1.035769	up	0.00376
PPP1R12C	protein phosphatase 1, regulatory subunit 12C	0.742943761	up	5.31E-05

PPP1R13B	protein phosphatase 1, regulatory subunit 13B	0.678810243	up	0.006022
PPP1R13L	protein phosphatase 1, regulatory subunit 13 like	0.748344955	up	0.00047
PPP1R15A	protein phosphatase 1, regulatory subunit 15A	1.898222175	up	0.004874
PPP1R18	protein phosphatase 1, regulatory subunit 18	1.014245008	up	3.37E-08
PPP1R37	protein phosphatase 1, regulatory subunit 37	1.179930392	up	0.001565
PPP1R3B	protein phosphatase 1, regulatory subunit 3B	0.852792796	up	0.000628
PPP1R3F	protein phosphatase 1, regulatory subunit 3F	-0.83488132	down	0.005008
PPP2R2C	protein phosphatase 2, regulatory subunit B, gamma	-0.69957741	down	5.9E-05
PPP2R5B	protein phosphatase 2, regulatory subunit B', beta	0.685567917	up	0.000486
PPP2R5C	protein phosphatase 2, regulatory subunit B', gamma	1.200505999	up	0.035795
PPP3CB	protein phosphatase 3, catalytic subunit, beta isozyme	-1.4429935	down	1.26E-15
PPP6R3	protein phosphatase 6, regulatory subunit 3	-1.94956164	down	0.009744
PPT2	palmitoyl-protein thioesterase 2	0.670850704	up	0.049787
PQBP1	polyglutamine binding protein 1	0.637821953	up	0.000207
PQLC2L	PQ loop repeat containing 2-like	1.487356704	up	0.00466
PRAF2	PRA1 domain family, member 2	0.742278215	up	8.17E-06
PRC1	protein regulator of cytokinesis 1	-1.25995937	down	2.46E-17
PRDM1	PR domain containing 1, with ZNF domain	-0.67233102	down	0.049793
PRDX1	peroxiredoxin 1	-0.59745105	down	0.000122
PRDX3	peroxiredoxin 3	-2.2572716	down	8.71E-51
PRELID3A	slowmo homolog 1 (Drosophila)	1.208992668	up	0.000296
PREPL	prolyl endopeptidase-like	-1.04824484	down	7.81E-08
PRICKLE1	prickle homolog 1 (Drosophila)	-0.7002146	down	0.040438

PRICKLE2	prickle homolog 2 (Drosophila)	0.59775216	up	0.014471
PRICKLE3	prickle homolog 3 (Drosophila)	0.632942512	up	0.000308
PRIM1	primase, DNA, polypeptide 1 (49kDa)	-0.77439535	down	0.000241
PRIM2	primase, DNA, polypeptide 2 (58kDa)	-1.04175663	down	0.00551
PRIMPOL	primase and polymerase (DNA-directed)	-0.65211338	down	0.033431
PRKAB2	protein kinase, AMP-activated, beta 2 non-catalytic subunit	1.447230777	up	3.96E-17
PRKCH	protein kinase C, eta	-0.78020815	down	0.00547
PRKD2	protein kinase D2	0.726346825	up	6.46E-05
PRKDC	protein kinase, DNA-activated, catalytic polypeptide	-0.83990324	down	0.001621
PRKX	protein kinase, X-linked	-1.069912	down	9.56E-07
PRNP	prion protein	-1.22919089	down	5.82E-12
PROCA1	protein interacting with cyclin A1	-0.8994987	down	0.017439
PROSER3	proline and serine rich 3	-0.83774053	down	0.000342
PRPF40B	PRP40 pre-mRNA processing factor 40 homolog B (S. cerevisiae)	0.605353392	up	0.001185
PRPS2	phosphoribosyl pyrophosphate synthetase 2	-1.65410316	down	3.54E-23
PRR15	proline rich 15	-0.76368487	down	0.035363
PRR5	proline rich 5 (renal)	0.59298056	up	0.033858
PRRT3	proline-rich transmembrane protein 3	1.02158856	up	0.01581
PRSS23	protease, serine, 23	-1.00799825	down	4.17E-12
PRSS3	protease, serine, 3	1.471749508	up	0.017192
PRSS53	protease, serine, 53	0.75550123	up	0.02458
PRTFDC1	phosphoribosyl transferase domain containing 1	-1.37658255	down	0.002633
PSAT1	phosphoserine aminotransferase 1	-1.26860519	down	2.79E-06
PSG2	pregnancy specific beta-1-glycoprotein 2	-20	down	0.03066

				4
PSIP1	PC4 and SFRS1 interacting protein 1	-0.74867796	down	9.29E-06
PSMA4	proteasome (prosome, macropain) subunit, alpha type, 4	-0.89512908	down	2.16E-09
PSMA6	proteasome (prosome, macropain) subunit, alpha type, 6	0.59457666	up	0.000442
PSMB9	proteasome (prosome, macropain) subunit, beta type, 9	0.640744616	up	0.002838
PSMD10	proteasome (prosome, macropain) 26S subunit, non-ATPase, 10	-0.86126214	down	4.22E-07
PSME3	proteasome (prosome, macropain) activator subunit 3 (PA28 gamma; Ki)	-0.69672493	down	0.001619
PSPH	phosphoserine phosphatase	-1.26806266	down	3.79E-11
PTAFR	platelet-activating factor receptor	0.924300795	up	0.000329
PTCH1	patched 1	-0.69024167	down	0.000732
PTEN	phosphatase and tensin homolog	0.739488446	up	0.007516
PTGDS	prostaglandin D2 synthase 21kDa (brain)	1.328282682	up	0.000666
PTGES	prostaglandin E synthase	0.725506614	up	5.38E-06
PTGR1	prostaglandin reductase 1	-0.60308854	down	0.000117
PTGR2	prostaglandin reductase 2	-0.93180327	down	3.38E-06
PTGS2	prostaglandin-endoperoxide synthase 2 (prostaglandin G/H synthase and cyclooxygenase)	1.353607661	up	2.01E-11
PTHLH	parathyroid hormone-like hormone	0.632734303	up	0.015715
PTK2B	protein tyrosine kinase 2 beta	0.664399393	up	0.001398
PTK6	protein tyrosine kinase 6	1.754447557	up	2.94E-11
PTP4A1	protein tyrosine phosphatase type IVA, member 1	-1.53823996	down	0.035695
PTPN11	protein tyrosine phosphatase, non-receptor type 11	-1.70734113	down	1.6E-28
PTPN21	protein tyrosine phosphatase, non-receptor type 21	-0.91097071	down	0.000177
PTPRH	protein tyrosine phosphatase, receptor type, H	1.163721465	up	0.00031

				2
PTPRN2	protein tyrosine phosphatase, receptor type, N polypeptide 2	0.971514903	up	0.017227
PTPRR	protein tyrosine phosphatase, receptor type, R	2.082185354	up	0.001933
PTTG1	pituitary tumor-transforming 1	-0.64787324	down	5.01E-05
PTX3	pentraxin 3, long	2.590165665	up	8.82E-14
PUS3	pseudouridylate synthase 3	1.167868722	up	4.17E-06
PUS7	pseudouridylate synthase 7 (putative)	-0.9556867	down	6.48E-05
PUS7L	pseudouridylate synthase 7 homolog (S. cerevisiae)-like	-1.12264031	down	0.003737
PWWP2B	PWWP domain containing 2B	0.885760945	up	2.25E-05
PXDN	peroxidasin homolog (Drosophila)	1.400142487	up	2.96E-08
PXMP4	peroxisomal membrane protein 4, 24kDa	-0.6675593	down	0.005411
PXN	paxillin	-1.01536849	down	6.9E-11
PYGM	phosphorylase, glycogen, muscle	1.997752033	up	0.006556
PYGO1	pygopus family PHD finger 1	-1.03762473	down	0.000332
QKI	QKI, KH domain containing, RNA binding	-0.80589492	down	3.85E-06
QSOX1	quiescin Q6 sulfhydryl oxidase 1	0.860986919	up	6.02E-09
R3HCC1	R3H domain and coiled-coil containing 1	0.843184395	up	1.2E-06
R3HDM4	R3H domain containing 4	1.126815979	up	4.74E-07
RAB11FIP2	RAB11 family interacting protein 2 (class I)	-1.27366939	down	7.18E-09
RAB11FIP5	RAB11 family interacting protein 5 (class I)	0.713138354	up	5.95E-06
RAB1A	RAB1A, member RAS oncogene family	-1.28893802	down	1.7E-17
RAB22A	RAB22A, member RAS oncogene family	-0.78493879	down	5.53E-05
RAB26	RAB26, member RAS oncogene family	-0.77250852	down	7E-05
RAB29	RAB29, member RAS oncogene family	-1.45776328	down	3.74E-16

RAB32	RAB32, member RAS oncogene family	-1.2188701	down	1.23E-07
RAB39B	RAB39B, member RAS oncogene family	2.09006901	up	0.000154
RAB3A	RAB3A, member RAS oncogene family	1.115802342	up	0.00102
RAB3IL1	RAB3A interacting protein (rabin3)-like 1	0.891765395	up	9.05E-07
RAB40C	RAB40C, member RAS oncogene family	1.075669696	up	0.01238
RAB9A	RAB9A, member RAS oncogene family	0.929961334	up	8.17E-08
RABL3	RAB, member of RAS oncogene family-like 3	-0.70167677	down	0.013576
RAC2	ras-related C3 botulinum toxin substrate 2 (rho family, small GTP binding protein Rac2)	2.05007241	up	1.52E-06
RACGAP1	Rac GTPase activating protein 1	-0.74833779	down	2.48E-06
RAD50	RAD50 homolog (S. cerevisiae)	-0.79831717	down	7.06E-05
RAD51	RAD51 recombinase	-1.02200016	down	1.66E-05
RAD51AP1	RAD51 associated protein 1	-2.20868162	down	4.8E-11
RAD54B	RAD54 homolog B (S. cerevisiae)	-0.87080688	down	0.002152
RAD54L	RAD54-like (S. cerevisiae)	-1.74971134	down	2.53E-18
RAI14	retinoic acid induced 14	0.970257796	up	3.34E-10
RALB	v-ral simian leukemia viral oncogene homolog B	-1.85236901	down	9.98E-30
RALGDS	ral guanine nucleotide dissociation stimulator	0.917080845	up	1.97E-05
RALGPS2	Ral GEF with PH domain and SH3 binding motif 2	-1.44846489	down	0.027359
RANBP1	RAN binding protein 1	-1.04892413	down	3.21E-10
RANBP10	RAN binding protein 10	0.85120084	up	2.74E-06
RARRES1	retinoic acid receptor responder (tazarotene induced) 1	-1.17594131	down	0.000139
RASA3	RAS p21 protein activator 3	0.643619126	up	0.000141
RASA4	RAS p21 protein activator 4	2.240431772	up	1.04E-06

RASA4B	RAS p21 protein activator 4B	1.729979148	up	0.000344
RASAL1	RAS protein activator like 1 (GAP1 like)	1.12151042	up	0.000878
RASD1	RAS, dexamethasone-induced 1	2.146338123	up	0.008274
RASD2	RASD family, member 2	-0.62152068	down	0.007941
RASGRF1	Ras protein-specific guanine nucleotide-releasing factor 1	3.626542282	up	0.00736
RASGRP2	RAS guanyl releasing protein 2 (calcium and DAG-regulated)	0.961772395	up	0.000428
RASL11A	RAS-like, family 11, member A	-0.87620661	down	0.002742
RASSF1	Ras association (RalGDS/AF-6) domain family member 1	1.087319702	up	6.77E-05
RASSF2	Ras association (RalGDS/AF-6) domain family member 2	-1.2824029	down	1.53E-15
RASSF9	Ras association (RalGDS/AF-6) domain family (N-terminal) member 9	-1.21792195	down	1.84E-05
RBCK1	RanBP-type and C3HC4-type zinc finger containing 1	0.911128229	up	1.55E-09
RBKS	ribokinase	0.892328925	up	2.15E-05
RBL1	retinoblastoma-like 1	-0.96807269	down	0.000714
RBM18	RNA binding motif protein 18	0.703720103	up	0.000118
RBM23	RNA binding motif protein 23	-1.04301059	down	4.14E-10
RBM26	RNA binding motif protein 26	-0.83586926	down	1.94E-05
RBM28	RNA binding motif protein 28	-0.95044322	down	0.002199
RBM47	RNA binding motif protein 47	-1.08495477	down	7.51E-12
RBMS1	RNA binding motif, single stranded interacting protein 1	-0.80872044	down	1.15E-06
RBMX	RNA binding motif protein, X-linked	-0.60562832	down	0.001276
RC3H2	ring finger and CCCH-type domains 2	-0.89409042	down	1.02E-05
RCBTB1	regulator of chromosome condensation (RCC1) and BTB (POZ) domain containing protein 1	-1.43412023	down	1.41E-13

RCN2	reticulocalbin 2, EF-hand calcium binding domain	-0.61011558	down	0.009548
RDH11	retinol dehydrogenase 11 (all-trans/9-cis/11-cis)	-1.80969077	down	1.91E-30
RDM1	RAD52 motif containing 1	-1.23739098	down	1.67E-05
RECQL	RecQ helicase-like	-1.02495569	down	1.73E-09
RECQL4	RecQ protein-like 4	-0.89286686	down	1.75E-05
REEP2	receptor accessory protein 2	0.644096476	up	0.000626
REEP5	receptor accessory protein 5	-0.6926302	down	0.000652
RELL2	RELT-like 2	0.70149958	up	0.002144
REM2	RAS (RAD and GEM)-like GTP binding 2	1.086591602	up	0.009018
RENBP	renin binding protein	2.849223662	up	0.000131
RERG	RAS-like, estrogen-regulated, growth inhibitor	-1.31719439	down	0.020581
RET	ret proto-oncogene	1.135755602	up	7.93E-05
RETSAT	retinol saturase (all-trans-retinol 13,14-reductase)	0.738056484	up	6.43E-06
RFC1	replication factor C (activator 1) 1, 145kDa	-0.95265081	down	2.31E-06
RFC2	replication factor C (activator 1) 2, 40kDa	-0.98033843	down	7.29E-09
RFC3	replication factor C (activator 1) 3, 38kDa	-1.10282742	down	0.000143
RFC4	replication factor C (activator 1) 4, 37kDa	-0.8737585	down	6.84E-05
RFC5	replication factor C (activator 1) 5, 36.5kDa	-0.66223185	down	0.000189
RFFL	ring finger and FYVE-like domain containing E3 ubiquitin protein ligase	1.197829073	up	3.14E-08
RFWD3	ring finger and WD repeat domain 3	-0.67290649	down	5.99E-05
RFX2	regulatory factor X, 2 (influences HLA class II expression)	0.868399582	up	8.53E-05
RFX7	regulatory factor X, 7	-0.67385567	down	0.013027

RGS10	regulator of G-protein signaling 10	0.814355258	up	1.87E-06
RGS11	regulator of G-protein signaling 11	0.803304174	up	0.009812
RGS14	regulator of G-protein signaling 14	0.821819368	up	0.002216
RGS16	regulator of G-protein signaling 16	1.427873503	up	0.008191
RGS17	regulator of G-protein signaling 17	0.938119198	up	0.005091
RGS2	regulator of G-protein signaling 2	0.7328813	up	0.000295
RGS9	regulator of G-protein signaling 9	1.976514427	up	7.62E-06
RHCG	Rh family, C glycoprotein	1.958264287	up	1.6E-28
RHOC	ras homolog family member C	0.617175038	up	0.000128
RHOD	ras homolog family member D	0.878935619	up	0.001899
RHPN2	rhophilin, Rho GTPase binding protein 2	0.938801226	up	2.61E-10
RIC3	RIC3 acetylcholine receptor chaperone	1.454024553	up	0.006162
RIF1	replication timing regulatory factor 1	-1.20096537	down	0.000235
RILP	Rab interacting lysosomal protein	1.066471548	up	5.89E-09
RIN1	Ras and Rab interactor 1	1.516268754	up	2.28E-13
RIOK3	RIO kinase 3	0.908968413	up	0.002241
RIPK2	receptor-interacting serine-threonine kinase 2	0.79851111	up	0.00049
RMI2	RecQ mediated genome instability 2	-1.21002877	down	3.75E-10
RMND1	required for meiotic nuclear division 1 homolog (S. cerevisiae)	0.962937626	up	0.001132
RNASEH2A	ribonuclease H2, subunit A	-0.93026891	down	1.01E-07
RNF103	ring finger protein 103	1.043288089	up	4.82E-06
RNF11	ring finger protein 11	-0.93813789	down	0.000374
RNF113A	ring finger protein 113A	0.733452354	up	5.26E-0

				5
RNF19B	ring finger protein 19B	1.027596451	up	0.00011<br>1
RNF212	ring finger protein 212	1.356931018	up	3.01E-0<br>6
RNF215	ring finger protein 215	0.625080829	up	0.00166<br>7
RNF217	ring finger protein 217	-1.19121134	down	2.32E-0<br>9
RNF223	ring finger protein 223	-1.86394713	down	0.02597
RNF24	ring finger protein 24	0.826891636	up	2.38E-0<br>7
RNF25	ring finger protein 25	0.781920451	up	2.68E-0<br>5
RNF26	ring finger protein 26	-0.99163419	down	1.35E-0<br>9
RNF31	ring finger protein 31	0.586126374	up	0.00061<br>9
RNF38	ring finger protein 38	1.335998099	up	8.7E-15
RNF43	ring finger protein 43	-0.72171173	down	0.00014<br>4
RNGTT	RNA guanylyltransferase and 5'-phosphatase	-1.51902754	down	2.72E-1<br>3
ROCK2	Rho-associated, coiled-coil containing protein kinase 2	-1.69517785	down	4.19E-2<br>3
ROR2	receptor tyrosine kinase-like orphan receptor 2	1.190564004	up	0.00462<br>3
RORA	RAR-related orphan receptor A	1.746742825	up	0.01948<br>2
RP1L1	retinitis pigmentosa 1-like 1	-1.7018515	down	0.00543<br>7
RP2	retinitis pigmentosa 2 (X-linked recessive)	-0.73562808	down	0.00094<br>6
RP9	retinitis pigmentosa 9 (autosomal dominant)	0.784278556	up	0.00039
RPA1	replication protein A1, 70kDa	-0.62061795	down	0.00689<br>4
RPE	ribulose-5-phosphate-3-epimerase	-0.84409715	down	2.76E-0<br>5
RPEL1	ribulose-5-phosphate-3-epimerase-like 1	-1.43938159	down	1.59E-0<br>6
RPGRIP1L	RPGRIP1-like	-0.80246804	down	0.00413<br>6
RPIA	ribose 5-phosphate isomerase A	-1.28177089	down	0.00160

				5
RPL10	ribosomal protein L10	0.713487697	up	2.03E-06
RPL12	ribosomal protein L12	0.618032042	up	5.62E-05
RPL13	ribosomal protein L13	0.619966055	up	0.002742
RPL13A	ribosomal protein L13a	1.11581223	up	3.44E-05
RPL18A	ribosomal protein L18a	0.654587672	up	0.000983
RPL23	ribosomal protein L23	0.595150513	up	0.003154
RPL27A	ribosomal protein L27a	0.960562987	up	0.001093
RPL28	ribosomal protein L28	0.954510861	up	3.64E-11
RPL32	ribosomal protein L32	0.596886506	up	0.000195
RPP25L	ribonuclease P/MRP 25kDa subunit-like	-1.59008596	down	4.41E-13
RPS10-NUDT3	RPS10-NUDT3 readthrough	-1.33499495	down	7.96E-10
RPS12	ribosomal protein S12	0.631344087	up	0.000114
RPS19	ribosomal protein S19	0.64102391	up	0.008213
RPS25	ribosomal protein S25	0.686988095	up	8.7E-06
RPS27	ribosomal protein S27	1.101754543	up	7.42E-11
RPS28	ribosomal protein S28	1.337590718	up	0.026019
RPS6KB1	ribosomal protein S6 kinase, 70kDa, polypeptide 1	-0.98534208	down	0.033926
RRAD	Ras-related associated with diabetes	2.582921792	up	2.05E-10
RRAGA	Ras-related GTP binding A	0.650158022	up	2.82E-05
RRAGD	Ras-related GTP binding D	1.354844582	up	5.18E-12
RRM1	ribonucleotide reductase M1	-1.2619006	down	3.83E-16
RRM2	ribonucleotide reductase M2	-1.43273782	down	1.04E-1

				8
RRP12	ribosomal RNA processing 12 homolog (S. cerevisiae)	0.856365438	up	3.81E-07
RRP15	ribosomal RNA processing 15 homolog (S. cerevisiae)	-0.94419959	down	0.003368
RSPH9	radial spoke head 9 homolog (Chlamydomonas)	1.017923943	up	0.012925
RTCA	RNA 3'-terminal phosphate cyclase	0.588143767	up	0.018125
RTFDC1	replication termination factor 2 domain containing 1	-0.93253187	down	5.35E-07
RTN2	reticulon 2	0.723636423	up	8.43E-05
RTN4	reticulon 4	-0.76684986	down	6.27E-07
RTTN	rotatin	0.618819019	up	0.000583
RUFY2	RUN and FYVE domain containing 2	-0.68664578	down	0.005647
RUNDC3A	RUN domain containing 3A	1.953791058	up	0.004271
RUNX1	runt-related transcription factor 1	0.841278106	up	0.000183
RUNX2	runt-related transcription factor 2	1.697254566	up	1.09E-05
RYK	receptor-like tyrosine kinase	-0.61104546	down	0.007598
S100A3	S100 calcium binding protein A3	-0.62310478	down	0.00806
S100A9	S100 calcium binding protein A9	2.348297474	up	0.000226
S1PR3	sphingosine-1-phosphate receptor 3	-1.05893527	down	2.74E-09
S1PR5	sphingosine-1-phosphate receptor 5	-1.43290947	down	0.000371
SAA2	serum amyloid A2	1.210675701	up	0.00044
SAMD1	sterile alpha motif domain containing 1	-0.64898519	down	0.0118
SAMD14	sterile alpha motif domain containing 14	2.130193655	up	9.95E-09
SAT2	spermidine/spermine N1-acetyltransferase family member 2	0.856701869	up	5.95E-08
SAYSD1	SAYSVFN motif domain containing 1	-0.92480768	down	0.00102
SBDS	Shwachman-Bodian-Diamond syndrome	0.67778861	up	8.26E-05

SBNO2	strawberry notch homolog 2 (Drosophila)	0.653800873	up	0.009241
SBSN	suprabasin	3.204627399	up	1.23E-11
SCAF11	SR-related CTD-associated factor 11	-0.81607744	down	7.69E-06
SCAI	suppressor of cancer cell invasion	-0.82658501	down	0.018643
SCAMP2	secretory carrier membrane protein 2	-1.17445857	down	7.84E-12
SCARA3	scavenger receptor class A, member 3	-1.26828951	down	4.75E-16
SCD	stearoyl-CoA desaturase (delta-9-desaturase)	-1.64643309	down	2.85E-22
SCG2	secretogranin II	0.7854857	up	0.022726
SCML1	sex comb on midleg-like 1 (Drosophila)	1.135809254	up	1.46E-07
SCML2	sex comb on midleg-like 2 (Drosophila)	0.719843915	up	0.000371
SCN4A	sodium channel, voltage-gated, type IV, alpha subunit	3.110990634	up	9.87E-09
SCN4B	sodium channel, voltage-gated, type IV, beta subunit	2.550755361	up	0.023982
SCN5A	sodium channel, voltage-gated, type V, alpha subunit	0.952023037	up	0.028579
SCNN1A	sodium channel, non-voltage-gated 1 alpha subunit	-0.82124155	down	0.00026
SCO2	SCO2 cytochrome c oxidase assembly protein	0.746188315	up	0.000235
SCRN1	secernin 1	-0.83486043	down	5.94E-06
SDC1	syndecan 1	-0.74298494	down	4.32E-06
SDCBP	syndecan binding protein (syntenin)	0.617593734	up	0.000214
SDHAF3	ACN9 homolog (S. cerevisiae)	-1.35158167	down	1.03E-06
SDHAF4	chromosome 6 open reading frame 57	-0.91741269	down	0.010118
SDPR	serum deprivation response	-1.26357233	down	3.61E-13
SDR16C5	short chain dehydrogenase/reductase family 16C, member 5	1.182619621	up	0.00362
SEC14L2	SEC14-like 2 (S. cerevisiae)	1.18467113	up	2.34E-1

				0
SEC23IP	SEC23 interacting protein	-0.67053135	down	0.000141
SEC24D	SEC24 family member D	0.639853399	up	0.000193
SEC61G	Sec61 gamma subunit	0.588511894	up	0.031511
SELPLG	selectin P ligand	1.2919457	up	0.019828
SEMA3A	sema domain, immunoglobulin domain (Ig), short basic domain, secreted, (semaphorin) 3A	0.715874866	up	1.84E-05
SEMA4B	sema domain, immunoglobulin domain (Ig), transmembrane domain (TM) and short cytoplasmic domain, (semaphorin) 4B	0.856734945	up	0.037302
SEMA4C	sema domain, immunoglobulin domain (Ig), transmembrane domain (TM) and short cytoplasmic domain, (semaphorin) 4C	0.988622526	up	9.75E-09
SEMA4F	sema domain, immunoglobulin domain (Ig), transmembrane domain (TM) and short cytoplasmic domain, (semaphorin) 4F	-0.64390856	down	0.00082
SEMA6A	sema domain, transmembrane domain (TM), and cytoplasmic domain, (semaphorin) 6A	3.260913323	up	0.017692
SEMA7A	semaphorin 7A, GPI membrane anchor (John Milton Hagen blood group)	2.531674985	up	5.24E-08
SENP2	SUMO1/sentrin/SMT3 specific peptidase 2	-0.90456117	down	0.004421
SEPHS1	selenophosphate synthetase 1	-0.65621479	down	0.000203
SEPT2	septin 2	-1.27669311	down	4.03E-18
SEPT3	septin 3	1.301112391	up	8.16E-10
SEPT4	septin 4	1.251786243	up	9.98E-08
SEPT5	septin 5	2.003590926	up	0.036785
SEPT9	septin 9	0.708486299	up	0.000105
SERHL2	serine hydrolase-like 2	2.690234591	up	0.021258
SERINC1	serine incorporator 1	0.665939438	up	5.46E-05
SERINC2	serine incorporator 2	0.947688127	up	3.3E-08
SERINC5	serine incorporator 5	-1.14474338	down	1.89E-06
SERPINA1	serpin peptidase inhibitor, clade A (alpha-1 antiproteinase,	0.904803616	up	0.03586

	antitrypsin), member 1			7
SERPINB7	serpin peptidase inhibitor, clade B (ovalbumin), member 7	1.253030674	up	0.00239 3
SERPINE1	serpin peptidase inhibitor, clade E (nexin, plasminogen activator inhibitor type 1), member 1	0.651801027	up	0.00781 3
SERPINE2	serpin peptidase inhibitor, clade E (nexin, plasminogen activator inhibitor type 1), member 2	1.164194207	up	7.43E-1 5
SERPING1	serpin peptidase inhibitor, clade G (C1 inhibitor), member 1	1.476341476	up	0.03438 6
SERTAD2	SERTA domain containing 2	0.848752005	up	7.38E-0 5
SESN1	sestrin 1	1.009030533	up	4.31E-0 5
SETD7	SET domain containing (lysine methyltransferase) 7	-1.90229798	down	9.01E-1 7
SETMAR	SET domain and mariner transposase fusion gene	-0.79430692	down	0.04641
SF3A2	splicing factor 3a, subunit 2, 66kDa	0.627689459	up	0.00040 5
SFMBT2	Scm-like with four mbt domains 2	1.001521403	up	7.21E-0 9
SFPQ	splicing factor proline/glutamine-rich	-0.92699881	down	4.5E-05
SFR1	SWI5-dependent recombination repair 1	1.290410163	up	1.19E-1 0
SFRP1	secreted frizzled-related protein 1	0.668256824	up	5.6E-05
SFRP4	secreted frizzled-related protein 4	-1.52545031	down	0.00194 3
SFT2D2	SFT2 domain containing 2	-0.80571314	down	0.01608 2
SFT2D3	SFT2 domain containing 3	-1.10138897	down	7.59E-0 7
SFXN1	sideroflexin 1	-0.81808569	down	1.61E-0 7
SFXN3	sideroflexin 3	0.587130424	up	0.00021 9
SGK2	serum/glucocorticoid regulated kinase 2	-0.77609306	down	0.01830 8
SGSM1	small G protein signaling modulator 1	0.98583308	up	0.00036 2
SH2D2A	SH2 domain containing 2A	1.029457189	up	0.02409 8
SH2D3C	SH2 domain containing 3C	1.83494473	up	0.00056 7
SH2D4A	SH2 domain containing 4A	-0.71636682	down	0.00010

				8
SH3BGRL3	SH3 domain binding glutamate-rich protein like 3	0.629047267	up	0.000271
SH3D19	SH3 domain containing 19	-1.04143611	down	2.93E-07
SH3D21	SH3 domain containing 21	1.628113802	up	1.61E-08
SH3GL3	SH3-domain GRB2-like 3	3.731276257	up	4.31E-05
SH3PXD2A	SH3 and PX domains 2A	0.612821191	up	0.005166
SHB	Src homology 2 domain containing adaptor protein B	0.691383303	up	0.001558
SHC2	SHC (Src homology 2 domain containing) transforming protein 2	1.059428407	up	0.004083
SHCBP1	SHC SH2-domain binding protein 1	-1.40911534	down	7.21E-17
SHF	Src homology 2 domain containing F	1.368221516	up	7.64E-07
SHMT1	serine hydroxymethyltransferase 1 (soluble)	-0.71827491	down	2.85E-05
SIK3	SIK family kinase 3	0.671828295	up	0.000323
SIMC1	SUMO-interacting motifs containing 1	-0.62662627	down	0.039991
SIN3B	SIN3 transcription regulator family member B	0.801971778	up	1.54E-06
SIRT2	sirtuin 2	0.71137571	up	9.37E-06
SIRT5	sirtuin 5	-0.65202264	down	0.010611
SIRT6	sirtuin 6	1.01963147	up	1.21E-06
SIRT7	sirtuin 7	0.864019757	up	2.27E-06
SKA1	spindle and kinetochore associated complex subunit 1	-0.72902137	down	0.003122
SKA3	spindle and kinetochore associated complex subunit 3	-1.35554454	down	1.84E-08
SKAP2	src kinase associated phosphoprotein 2	-0.61625986	down	0.000423
SKIL	SKI-like proto-oncogene	-0.80660487	down	0.000915

SKP2	S-phase kinase-associated protein 2, E3 ubiquitin protein ligase	-1.92374089	down	4.56E-07
SLBP	stem-loop binding protein	-0.67898811	down	8.45E-05
SLC11A1	solute carrier family 11 (proton-coupled divalent metal ion transporter), member 1	1.842833225	up	0.01546
SLC12A2	solute carrier family 12 (sodium/potassium/chloride transporter), member 2	-1.39295123	down	1.95E-05
SLC12A4	solute carrier family 12 (potassium/chloride transporter), member 4	0.80854662	up	8.6E-05
SLC12A7	solute carrier family 12 (potassium/chloride transporter), member 7	0.594296471	up	0.000431
SLC13A5	solute carrier family 13 (sodium-dependent citrate transporter), member 5	1.538803555	up	0.039937
SLC16A14	solute carrier family 16, member 14	1.173781134	up	0.023921
SLC16A2	solute carrier family 16, member 2 (thyroid hormone transporter)	1.280114939	up	0.000128
SLC16A6	solute carrier family 16, member 6	2.182544497	up	2.62E-18
SLC16A7	solute carrier family 16 (monocarboxylate transporter), member 7	-1.03317291	down	0.037557
SLC17A5	solute carrier family 17 (acidic sugar transporter), member 5	0.799000958	up	7.58E-05
SLC17A7	solute carrier family 17 (vesicular glutamate transporter), member 7	1.83636319	up	1.04E-15
SLC22A1	solute carrier family 22 (organic cation transporter), member 1	1.474571216	up	0.024012
SLC22A17	solute carrier family 22, member 17	0.724615524	up	0.003868
SLC22A18	solute carrier family 22, member 18	0.58957735	up	0.021156
SLC22A3	solute carrier family 22 (organic cation transporter), member 3	-0.60167705	down	0.008416
SLC22A4	solute carrier family 22 (organic cation/zwitterion transporter), member 4	1.322174256	up	0.000244
SLC23A1	solute carrier family 23 (ascorbic acid transporter), member 1	-2.06835951	down	3.49E-07
SLC24A4	solute carrier family 24 (sodium/potassium/calcium exchanger), member 4	1.875902155	up	0.005771
SLC25A13	solute carrier family 25 (aspartate/glutamate carrier), member 13	-0.71176208	down	0.027995
SLC25A15	solute carrier family 25 (mitochondrial carrier; ornithine transporter) member 15	-0.85731073	down	0.000864

SLC25A22	solute carrier family 25 (mitochondrial carrier: glutamate), member 22	0.865744962	up	4.96E-06
SLC25A24	solute carrier family 25 (mitochondrial carrier; phosphate carrier), member 24	-1.14604349	down	3.15E-10
SLC25A25	solute carrier family 25 (mitochondrial carrier; phosphate carrier), member 25	1.10510311	up	0.001509
SLC25A3	solute carrier family 25 (mitochondrial carrier; phosphate carrier), member 3	-0.75248749	down	7.47E-06
SLC25A30	solute carrier family 25, member 30	-0.72582813	down	0.000838
SLC25A35	solute carrier family 25, member 35	-0.83360326	down	0.000575
SLC25A37	solute carrier family 25 (mitochondrial iron transporter), member 37	0.813070586	up	7.23E-05
SLC25A38	solute carrier family 25, member 38	0.600347713	up	0.000999
SLC25A44	solute carrier family 25, member 44	-1.09854145	down	1.27E-10
SLC26A11	solute carrier family 26 (anion exchanger), member 11	0.762361448	up	3.45E-05
SLC26A2	solute carrier family 26 (anion exchanger), member 2	-0.65082552	down	0.009715
SLC26A6	solute carrier family 26 (anion exchanger), member 6	1.175651295	up	2.04E-11
SLC27A1	solute carrier family 27 (fatty acid transporter), member 1	0.618214931	up	0.000757
SLC27A2	solute carrier family 27 (fatty acid transporter), member 2	-0.92681638	down	1.13E-07
SLC2A11	solute carrier family 2 (facilitated glucose transporter), member 11	0.867759175	up	0.009397
SLC30A6	solute carrier family 30 (zinc transporter), member 6	-1.18713707	down	0.002218
SLC30A7	solute carrier family 30 (zinc transporter), member 7	-0.99259535	down	0.014293
SLC31A2	solute carrier family 31 (copper transporter), member 2	-0.66923504	down	0.018326
SLC35A2	solute carrier family 35 (UDP-galactose transporter), member A2	0.626118619	up	0.000215
SLC35A4	solute carrier family 35, member A4	-0.66640318	down	7.11E-05
SLC35B4	solute carrier family 35 (UDP-xylose/UDP-N-acetylglucosamine transporter), member B4	-0.62335537	down	0.00058
SLC35D2	solute carrier family 35 (UDP-GlcNAc/UDP-glucose transporter), member D2	1.195044978	up	1.36E-12

SLC35F3	solute carrier family 35, member F3	0.842142771	up	0.00071
SLC35F5	solute carrier family 35, member F5	-0.78796857	down	4.57E-05
SLC35G1	solute carrier family 35, member G1	-0.91184448	down	0.045017
SLC36A4	solute carrier family 36 (proton/amino acid symporter), member 4	-0.8795093	down	0.000363
SLC37A1	solute carrier family 37 (glucose-6-phosphate transporter), member 1	1.021678174	up	0.009982
SLC37A3	solute carrier family 37, member 3	-0.87781386	down	1.24E-06
SLC38A10	solute carrier family 38, member 10	-0.89226855	down	5.24E-05
SLC38A7	solute carrier family 38, member 7	0.796808488	up	1.51E-05
SLC40A1	solute carrier family 40 (iron-regulated transporter), member 1	-1.30649019	down	6.24E-09
SLC44A1	solute carrier family 44 (choline transporter), member 1	-0.99952773	down	3.2E-10
SLC45A3	solute carrier family 45, member 3	0.81966968	up	0.000158
SLC46A3	solute carrier family 46, member 3	-1.59315336	down	6.29E-08
SLC4A5	solute carrier family 4 (sodium bicarbonate cotransporter), member 5	1.227342109	up	0.000354
SLC4A7	solute carrier family 4, sodium bicarbonate cotransporter, member 7	-1.23524648	down	1.84E-05
SLC50A1	solute carrier family 50 (sugar efflux transporter), member 1	0.652731531	up	6.48E-05
SLC5A5	solute carrier family 5 (sodium/iodide cotransporter), member 5	1.372584815	up	0.002813
SLC6A12	solute carrier family 6 (neurotransmitter transporter), member 12	1.931596845	up	0.021583
SLC6A15	solute carrier family 6 (neutral amino acid transporter), member 15	0.588294286	up	0.002564
SLC6A8	solute carrier family 6 (neurotransmitter transporter), member 8	1.253115184	up	4.69E-14
SLC7A11	solute carrier family 7 (anionic amino acid transporter light chain, xc- system), member 11	-1.94744559	down	4.46E-23
SLC7A7	solute carrier family 7 (amino acid transporter light chain, y+L system), member 7	-0.62348838	down	0.00567
SLC8A2	solute carrier family 8 (sodium/calcium exchanger), member 2	1.679470565	up	0.004088
SLC9A2	solute carrier family 9, subfamily A (NHE2, cation proton antiporter	-0.84716041	down	0.01447

	2), member 2			1
SLC9A6	solute carrier family 9, subfamily A (NHE6, cation proton antiporter 6), member 6	-0.62936278	down	0.00243 5
SLC9A9	solute carrier family 9, subfamily A (NHE9, cation proton antiporter 9), member 9	1.852342777	up	0.02744 7
SLCO3A1	solute carrier organic anion transporter family, member 3A1	0.728807186	up	0.02743 4
SLF1	ankyrin repeat domain 32	-1.13382238	down	0.00036
SLFN11	schlafen family member 11	-0.66751275	down	0.04924 9
SLITRK6	SLIT and NTRK-like family, member 6	-2.93941953	down	0.04235 7
SLTM	SAFB-like, transcription modulator	-0.70017791	down	3.65E-0 5
SLX4	SLX4 structure-specific endonuclease subunit	-0.64254354	down	0.00300 9
SLX4IP	SLX4 interacting protein	-0.8389377	down	0.0242
SMAD6	SMAD family member 6	-0.73472941	down	2.84E-0 5
SMARCAD1	SWI/SNF-related, matrix-associated actin-dependent regulator of chromatin, subfamily a, containing DEAD/H box 1	-0.80471096	down	0.00014 7
SMARCD1	SWI/SNF related, matrix associated, actin dependent regulator of chromatin, subfamily d, member 1	-0.68491146	down	4.75E-0 5
SMARCD3	SWI/SNF related, matrix associated, actin dependent regulator of chromatin, subfamily d, member 3	0.928113243	up	2.7E-08
SMC1A	structural maintenance of chromosomes 1A	-1.05861828	down	6.92E-0 7
SMC2	structural maintenance of chromosomes 2	-1.6874414	down	3.26E-1 1
SMC4	structural maintenance of chromosomes 4	-0.86721483	down	0.00431 5
SMIM24	small integral membrane protein 24	20	up	0.04424 5
SMOX	spermine oxidase	1.280491269	up	0.00015 9
SMPD1	sphingomyelin phosphodiesterase 1, acid lysosomal	0.992948839	up	4.36E-0 8
SMPD4	sphingomyelin phosphodiesterase 4, neutral membrane (neutral sphingomyelinase-3)	-1.78890425	down	2.73E-2 8
SMTNL2	smoothelin-like 2	1.991460814	up	0.00091 7
SMURF2	SMAD specific E3 ubiquitin protein ligase 2	0.739632559	up	1.99E-0 5

SNAI1	snail family zinc finger 1	1.381965942	up	0.027936
SNAI3	snail family zinc finger 3	1.789420642	up	0.003617
SNAP23	synaptosomal-associated protein, 23kDa	-0.6388313	down	0.004094
SNAP25	synaptosomal-associated protein, 25kDa	0.857213292	up	0.020171
SNAP91	synaptosomal-associated protein, 91kDa	1.48743802	up	0.006821
SNCB	synuclein, beta	1.93833626	up	0.000189
SNPH	syntaphilin	0.656685699	up	0.000513
SNRNP25	small nuclear ribonucleoprotein 25kDa (U11/U12)	-1.11449729	down	9.45E-09
SNRNP35	small nuclear ribonucleoprotein 35kDa (U11/U12)	-0.97359529	down	5.43E-05
SNRNP40	small nuclear ribonucleoprotein 40kDa (U5)	-0.8830387	down	5.05E-07
SNRPF	small nuclear ribonucleoprotein polypeptide F	-0.62012429	down	0.000369
SNTA1	syntrophin, alpha 1	0.621513088	up	0.000265
SNUPN	snurportin 1	-0.63043709	down	0.000954
SNX15	sorting nexin 15	0.808729826	up	1.78E-05
SNX19	sorting nexin 19	-0.72318105	down	2.73E-05
SNX2	sorting nexin 2	0.648539371	up	0.000254
SNX21	sorting nexin family member 21	0.843610164	up	0.000292
SOAT1	sterol O-acyltransferase 1	-1.51486815	down	0.000181
SOBP	sine oculis binding protein homolog (Drosophila)	0.894265147	up	0.017279
SOCS1	suppressor of cytokine signaling 1	1.861202475	up	0.009181
SOCS3	suppressor of cytokine signaling 3	0.667775098	up	0.000475
SORBS1	sorbin and SH3 domain containing 1	0.633353623	up	0.014475

SORT1	sortilin 1	-0.74153033	down	1.49E-05
SOWAHB	sosondowah ankyrin repeat domain family member B	2.583461591	up	0.005605
SP100	SP100 nuclear antigen	0.915685854	up	6.47E-09
SP110	SP110 nuclear body protein	1.011734966	up	0.000119
SP140L	SP140 nuclear body protein-like	0.72297516	up	0.011372
SP2	Sp2 transcription factor	-0.87991671	down	3.39E-05
SPAG16	sperm associated antigen 16	-1.13705273	down	1.55E-06
SPAG4	sperm associated antigen 4	0.676937745	up	0.002373
SPAG5	sperm associated antigen 5	-1.25250932	down	1.96E-16
SPARC	secreted protein, acidic, cysteine-rich (osteonectin)	2.176342749	up	0.001398
SPATA2	spermatogenesis associated 2	0.720615641	up	0.000373
SPATA20	spermatogenesis associated 20	0.58721839	up	0.000235
SPATA2L	spermatogenesis associated 2-like	0.939812859	up	1.11E-05
SPATA7	spermatogenesis associated 7	0.956948438	up	3.26E-05
SPC24	SPC24, NDC80 kinetochore complex component	-1.16730019	down	3.15E-09
SPC25	SPC25, NDC80 kinetochore complex component	-2.10744903	down	1.4E-10
SPDEF	SAM pointed domain containing ETS transcription factor	-1.91722106	down	4.24E-05
SPDL1	spindle apparatus coiled-coil protein 1	-0.9320488	down	5.4E-08
SPEG	SPEG complex locus	0.601612913	up	0.015669
SPIDR	scaffolding protein involved in DNA repair	-0.59406921	down	0.024192
SPIN4	spindlin family, member 4	-0.66732124	down	0.00774
SPINT1	serine peptidase inhibitor, Kunitz type 1	1.153342923	up	7.21E-05
SPNS2	spinster homolog 2 (Drosophila)	2.344733753	up	2.56E-10

SPOCD1	SPOC domain containing 1	1.67550628	up	4.73E-05
SPOP	speckle-type POZ protein	-0.74451817	down	0.000195
SPPL2A	signal peptide peptidase like 2A	-1.04254049	down	3.42E-05
SPRTN	SprT-like N-terminal domain	-0.6111648	down	0.014146
SPRY2	sprouty homolog 2 (Drosophila)	0.611225301	up	0.007329
SPRYD7	SPRY domain containing 7	-0.89593989	down	0.004012
SPSB2	splA/ryanodine receptor domain and SOCS box containing 2	0.803452584	up	0.001353
SPTBN4	spectrin, beta, non-erythrocytic 4	0.75260203	up	0.006776
SPX	spexin hormone	0.827280918	up	0.006308
SQRDL	sulfide quinone reductase-like (yeast)	0.903014653	up	6.2E-09
SQSTM1	sequestosome 1	0.763240945	up	2.38E-07
SRCIN1	SRC kinase signaling inhibitor 1	0.675767123	up	0.001261
SREK1	splicing regulatory glutamine/lysine-rich protein 1	-0.73079771	down	0.00119
SREK1IP1	SREK1-interacting protein 1	-0.8204845	down	0.000439
SRSF1	serine/arginine-rich splicing factor 1	-1.0779454	down	2.49E-12
SRSF2	serine/arginine-rich splicing factor 2	-1.07578567	down	8.49E-10
SRSF3	serine/arginine-rich splicing factor 3	-0.61363866	down	0.000151
SRSF7	serine/arginine-rich splicing factor 7	-0.62736308	down	0.000131
SRSF8	serine/arginine-rich splicing factor 8	-0.76482168	down	0.000714
SRXN1	sulfiredoxin 1	-0.67955961	down	1.12E-05
SSBP2	single-stranded DNA binding protein 2	0.796241945	up	0.00015
SSFA2	sperm specific antigen 2	0.642558558	up	6.39E-05
SSSCA1	Sjogren syndrome/scleroderma autoantigen 1	0.697276271	up	5.54E-05

SSTR2	somatostatin receptor 2	2.669201288	up	0.021788
SSX2IP	synovial sarcoma, X breakpoint 2 interacting protein	-0.80274088	down	0.000419
ST3GAL4	ST3 beta-galactoside alpha-2,3-sialyltransferase 4	0.935672125	up	6.18E-06
ST6GAL1	ST6 beta-galactosamide alpha-2,6-sialyltranferase 1	-1.39121709	down	2.83E-09
ST6GAL2	ST6 beta-galactosamide alpha-2,6-sialyltranferase 2	-1.21034761	down	2.86E-07
ST6GALNAC1	ST6 (alpha-N-acetyl-neuraminyl-2,3-beta-galactosyl-1,3)-N-acetylgalactosaminide alpha-2,6-sialyltransferase 1	-20	down	0.000414
ST6GALNAC4	ST6 (alpha-N-acetyl-neuraminyl-2,3-beta-galactosyl-1,3)-N-acetylgalactosaminide alpha-2,6-sialyltransferase 4	1.007082702	up	4.48E-07
ST6GALNAC6	ST6 (alpha-N-acetyl-neuraminyl-2,3-beta-galactosyl-1,3)-N-acetylgalactosaminide alpha-2,6-sialyltransferase 6	0.76907862	up	2.1E-06
STAC	SH3 and cysteine rich domain	1.464936485	up	1.65E-10
STAC2	SH3 and cysteine rich domain 2	1.240938342	up	6.18E-05
STAG1	stromal antigen 1	-0.76346854	down	0.002435
STARD10	StAR-related lipid transfer (START) domain containing 10	0.604529473	up	0.000682
STARD13	StAR-related lipid transfer (START) domain containing 13	0.801519614	up	0.000593
STAT5B	signal transducer and activator of transcription 5B	-0.79149293	down	1.15E-06
STC1	stanniocalcin 1	2.02650614	up	9.62E-08
STC2	stanniocalcin 2	0.686444771	up	5.93E-05
STEAP2	STEAP family member 2, metalloreductase	-0.86748084	down	0.000602
STEAP3	STEAP family member 3, metalloreductase	0.639080252	up	0.00228
STIL	SCL/TAL1 interrupting locus	-1.27705896	down	8.17E-10
STK17A	serine/threonine kinase 17a	1.072418716	up	3.63E-06
STK19	serine/threonine kinase 19	0.770186757	up	0.0018
STK26	serine/threonine protein kinase 26	-1.35872205	down	2.5E-11

STK35	serine/threonine kinase 35	0.619705597	up	0.000234
STK38	serine/threonine kinase 38	-1.40408407	down	1.41E-14
STK4	serine/threonine kinase 4	-0.59091947	down	0.001073
STK40	serine/threonine kinase 40	1.118426183	up	8.35E-11
STMN3	stathmin-like 3	0.688454628	up	0.000182
STOML1	stomatin (EPB72)-like 1	1.156510895	up	4.1E-09
STOX2	storkhead box 2	-0.88613308	down	0.000411
STRN	striatin, calmodulin binding protein	-0.7898568	down	0.000245
STX17	syntaxin 17	-0.72235591	down	9.57E-05
STX1A	syntaxin 1A (brain)	1.17087621	up	8.64E-06
STX1B	syntaxin 1B	-0.78437593	down	0.004214
SUCLA2	succinate-CoA ligase, ADP-forming, beta subunit	-0.88469982	down	6.41E-06
SULT1A4	sulfotransferase family, cytosolic, 1A, phenol-preferring, member 4	-0.85908528	down	0.034601
SUMO3	small ubiquitin-like modifier 3	-1.18756098	down	7.83E-12
SUPT16H	suppressor of Ty 16 homolog (S. cerevisiae)	-0.87745247	down	9.22E-09
SUPT5H	suppressor of Ty 5 homolog (S. cerevisiae)	0.597472805	up	0.000138
SURF2	surfeit 2	0.796998125	up	0.002367
SUSD3	sushi domain containing 3	0.905016164	up	0.009148
SUSD4	sushi domain containing 4	2.12521736	up	1.96E-07
SUV39H1	suppressor of variegation 3-9 homolog 1 (Drosophila)	-1.09345585	down	1.92E-09
SUV39H2	suppressor of variegation 3-9 homolog 2 (Drosophila)	-0.71696014	down	0.007293
SWSAP1	SWIM-type zinc finger 7 associated protein 1	-1.30122192	down	0.008579

SYCE2	synaptonemal complex central element protein 2	-1.67861794	down	0.046472
SYNC	syncoilin, intermediate filament protein	-0.70977135	down	0.001839
SYNCRIP	synaptotagmin binding, cytoplasmic RNA interacting protein	-0.72949053	down	3.92E-05
SYNE3	spectrin repeat containing, nuclear envelope family member 3	1.151545133	up	0.001287
SYNGR2	synaptogyrin 2	0.590295462	up	0.000214
SYNGR3	synaptogyrin 3	0.889768996	up	0.000208
SYP	synaptophysin	0.647529049	up	0.019566
SYPL2	synaptophysin-like 2	3.526270343	up	0.004977
SYT13	synaptotagmin XIII	0.731752072	up	0.001807
SYT5	synaptotagmin V	2.477238548	up	0.001613
SYT7	synaptotagmin VII	0.777754172	up	0.005625
TACC1	transforming, acidic coiled-coil containing protein 1	-0.82611728	down	1.84E-07
TACC3	transforming, acidic coiled-coil containing protein 3	-1.05868427	down	5.46E-11
TACO1	translational activator of mitochondrially encoded cytochrome c oxidase I	-0.60094661	down	0.001428
TAF13	TAF13 RNA polymerase II, TATA box binding protein (TBP)-associated factor, 18kDa	-0.86303365	down	0.019634
TAF15	TAF15 RNA polymerase II, TATA box binding protein (TBP)-associated factor, 68kDa	-1.1488421	down	4.95E-08
TAF1D	TATA box binding protein (TBP)-associated factor, RNA polymerase I, D, 41kDa	1.447629409	up	3.93E-07
TAF2	TAF2 RNA polymerase II, TATA box binding protein (TBP)-associated factor, 150kDa	-0.96769867	down	1.61E-07
TAF6L	TAF6-like RNA polymerase II, p300/CBP-associated factor (PCAF)-associated factor, 65kDa	-0.8986138	down	0.019208
TAF8	TAF8 RNA polymerase II, TATA box binding protein (TBP)-associated factor, 43kDa	-0.66977273	down	0.0015
TAF9B	TAF9B RNA polymerase II, TATA box binding protein (TBP)-associated factor, 31kDa	-0.90154218	down	0.000626
TAGLN2	transgelin 2	-1.70335759	down	6.18E-31

TALDO1	transaldolase 1	-1.60398768	down	8.59E-28
TAP1	transporter 1, ATP-binding cassette, sub-family B (MDR/TAP)	1.065670635	up	1.51E-12
TARBP1	TAR (HIV-1) RNA binding protein 1	-0.83048966	down	1.94E-06
TBC1D10A	TBC1 domain family, member 10A	1.450852825	up	2.91E-10
TBC1D13	TBC1 domain family, member 13	-0.82342847	down	6.48E-06
TBC1D17	TBC1 domain family, member 17	0.69880783	up	0.000155
TBC1D3	TBC1 domain family, member 3	2.39285528	up	0.038085
TBC1D3F	TBC1 domain family, member 3F	1.11012458	up	0.048418
TBC1D3K	TBC1 domain family, member 3K	2.1045301	up	0.021517
TBC1D3L	TBC1 domain family, member 3L	1.189738009	up	0.000402
TBC1D8	TBC1 domain family, member 8 (with GRAM domain)	0.745087133	up	6.38E-06
TBCD	tubulin folding cofactor D	-0.69372533	down	0.000231
TBCEL	tubulin folding cofactor E-like	0.805090048	up	0.003432
TBL1XR1	transducin (beta)-like 1 X-linked receptor 1	-1.42055966	down	5.67E-16
TBPL1	TBP-like 1	1.289394691	up	6.41E-11
TBX19	T-box 19	0.775012851	up	0.039489
TBX3	T-box 3	0.71788018	up	0.000627
TCAF1	family with sequence similarity 115, member A	-0.65494119	down	0.007736
TCEAL3	transcription elongation factor A (SII)-like 3	0.607299759	up	0.029645
TCEAL8	transcription elongation factor A (SII)-like 8	0.651598243	up	0.011891
TCF19	transcription factor 19	-1.20481165	down	1.29E-08
TCF4	transcription factor 4	-1.77433427	down	8.5E-09

TCF7L2	transcription factor 7-like 2 (T-cell specific, HMG-box)	0.601343156	up	0.015339
TCHP	trichoplein, keratin filament binding	-0.68766089	down	0.001355
TCIRG1	T-cell, immune regulator 1, ATPase, H+ transporting, lysosomal V0 subunit A3	0.754073196	up	0.000401
TCN1	transcobalamin I (vitamin B12 binding protein, R binder family)	1.077137121	up	0.021299
TCP11L1	t-complex 11, testis-specific-like 1	-0.64335703	down	0.0006
TCP11L2	t-complex 11, testis-specific-like 2	0.654551381	up	0.006562
TCTEX1D2	Tctex1 domain containing 2	-1.04054249	down	0.007165
TDP1	tyrosyl-DNA phosphodiesterase 1	-0.75176642	down	0.000144
TDRKH	tudor and KH domain containing	-0.86236331	down	0.000156
TERF2IP	telomeric repeat binding factor 2, interacting protein	0.618837827	up	0.000449
TERT	telomerase reverse transcriptase	-1.00004066	down	0.006383
TESC	tescalcin	0.787017866	up	8.67E-05
TESK2	testis-specific kinase 2	1.235660668	up	0.000204
TFDP2	transcription factor Dp-2 (E2F dimerization partner 2)	-0.7999845	down	8.76E-06
TFR2	transferrin receptor 2	0.753087242	up	0.042857
TFRC	transferrin receptor	-0.9544109	down	2.93E-05
TGFBR1	transforming growth factor, beta receptor 1	-0.67671024	down	0.025693
TGFBR2	transforming growth factor, beta receptor II (70/80kDa)	0.984468708	up	0.003348
TGFBRAP1	transforming growth factor, beta receptor associated protein 1	-0.62213178	down	0.020581
TGM2	transglutaminase 2	-0.90983119	down	3.22E-05
TGOLN2	trans-golgi network protein 2	-1.36518645	down	2.21E-20
THAP3	THAP domain containing, apoptosis associated protein 3	0.746305177	up	0.000205

THAP8	THAP domain containing 8	0.746924654	up	0.006083
THEM4	thioesterase superfamily member 4	-0.75720723	down	0.000879
THEMIS2	thymocyte selection associated family member 2	1.726511203	up	4.74E-21
THOC6	THO complex 6 homolog (Drosophila)	-0.71967904	down	0.000147
THSD7A	thrombospondin, type I, domain containing 7A	-1.22598294	down	0.01032
THUMPD1	THUMP domain containing 1	-0.73388612	down	0.000151
TIAM2	T-cell lymphoma invasion and metastasis 2	0.872109044	up	0.000357
TICAM1	toll-like receptor adaptor molecule 1	0.691446469	up	0.017327
TICRR	TOPBP1-interacting checkpoint and replication regulator	-1.11836887	down	6.36E-10
TIMELESS	timeless circadian clock	-1.07621076	down	7.13E-11
TIMM21	translocase of inner mitochondrial membrane 21 homolog (yeast)	-1.09903385	down	5.49E-07
TINAGL1	tubulointerstitial nephritis antigen-like 1	1.042871682	up	9.59E-06
TIPARP	TCDD-inducible poly(ADP-ribose) polymerase	1.515593029	up	6.93E-07
TJAP1	tight junction associated protein 1 (peripheral)	0.625285882	up	0.010064
TJP3	tight junction protein 3	0.888906407	up	0.000104
TK1	thymidine kinase 1, soluble	-0.63884586	down	0.000168
TLDC1	TBC/LysM-associated domain containing 1	0.680815842	up	2.84E-05
TLK1	tousled-like kinase 1	-0.61618696	down	0.00267
TLR2	toll-like receptor 2	2.51715848	up	0.009378
TM2D2	TM2 domain containing 2	-0.74261187	down	3.83E-05
TM4SF19	transmembrane 4 L six family member 19	0.692336981	up	0.010693
TM4SF20	transmembrane 4 L six family member 20	-2.56108111	down	1.75E-09
TM4SF4	transmembrane 4 L six family member 4	-2.51125518	down	2.06E-2

				7
TMC6	transmembrane channel-like 6	0.910122904	up	3.15E-05
TMCO3	transmembrane and coiled-coil domains 3	-0.69841268	down	1.9E-05
TMED8	transmembrane emp24 protein transport domain containing 8	-1.65717611	down	4.31E-19
TMEM104	transmembrane protein 104	-0.62730773	down	0.047032
TMEM106A	transmembrane protein 106A	1.077974424	up	0.000208
TMEM106B	transmembrane protein 106B	-1.00927347	down	9.75E-07
TMEM109	transmembrane protein 109	-1.38215911	down	1.47E-18
TMEM132A	transmembrane protein 132A	1.078794285	up	4.58E-13
TMEM139	transmembrane protein 139	-1.81162563	down	0.000123
TMEM140	transmembrane protein 140	1.072769627	up	3.85E-07
TMEM141	transmembrane protein 141	0.62188526	up	0.002916
TMEM150A	transmembrane protein 150A	0.918233404	up	0.002785
TMEM156	transmembrane protein 156	0.775354073	up	0.000149
TMEM158	transmembrane protein 158 (gene/pseudogene)	1.539475505	up	6.11E-10
TMEM159	transmembrane protein 159	1.118586138	up	7.91E-11
TMEM167A	transmembrane protein 167A	-0.66114089	down	0.006244
TMEM167B	transmembrane protein 167B	1.378417142	up	2.08E-05
TMEM170A	transmembrane protein 170A	-0.68482454	down	0.006391
TMEM184C	transmembrane protein 184C	-0.58890648	down	0.004029
TMEM189	transmembrane protein 189	0.656657959	up	9.45E-05
TMEM19	transmembrane protein 19	-1.45265167	down	0.000366
TMEM199	transmembrane protein 199	-0.64654874	down	0.01701

				4
TMEM209	transmembrane protein 209	-1.43425213	down	2.06E-12
TMEM221	transmembrane protein 221	1.191141941	up	0.00356
TMEM223	transmembrane protein 223	-1.08280825	down	1.69E-07
TMEM229B	transmembrane protein 229B	0.840543031	up	0.010695
TMEM237	transmembrane protein 237	-1.58489426	down	0.000633
TMEM255A	transmembrane protein 255A	1.336734648	up	0.047067
TMEM265	uncharacterized LOC100862671	1.510123588	up	1.7E-14
TMEM37	transmembrane protein 37	-0.60839607	down	0.013474
TMEM38A	transmembrane protein 38A	1.505240341	up	2.41E-05
TMEM39A	transmembrane protein 39A	0.719285147	up	0.000135
TMEM41A	transmembrane protein 41A	-0.90667517	down	8.45E-07
TMEM41B	transmembrane protein 41B	-0.68782401	down	0.000629
TMEM51	transmembrane protein 51	1.390351716	up	0.049851
TMEM52	transmembrane protein 52	0.930148997	up	0.038472
TMEM54	transmembrane protein 54	-1.64563461	down	6.45E-17
TMEM55A	transmembrane protein 55A	1.487067731	up	1.34E-12
TMEM55B	transmembrane protein 55B	0.649794939	up	0.000179
TMEM57	transmembrane protein 57	0.756187947	up	0.000226
TMEM59L	transmembrane protein 59-like	0.832736264	up	0.000875
TMEM63B	transmembrane protein 63B	0.594456139	up	0.000602
TMEM64	transmembrane protein 64	-1.73669604	down	3.03E-17
TMEM68	transmembrane protein 68	-1.2239535	down	2.36E-06

TMEM74B	transmembrane protein 74B	0.88484113	up	0.001565
TMEM79	transmembrane protein 79	0.731038567	up	0.01062
TMEM86A	transmembrane protein 86A	1.733137261	up	1.47E-08
TMEM97	transmembrane protein 97	-0.71778791	down	6.39E-05
TMPO	thymopoietin	-1.48160696	down	1.25E-11
TMSB10	thymosin beta 10	0.70332537	up	2.45E-05
TMUB1	transmembrane and ubiquitin-like domain containing 1	0.651408384	up	0.027394
TMX4	thioredoxin-related transmembrane protein 4	-0.81267768	down	3.29E-06
TNC	tenascin C	1.176977972	up	5.3E-08
TNFAIP3	tumor necrosis factor, alpha-induced protein 3	1.933793742	up	0.000623
TNFAIP6	tumor necrosis factor, alpha-induced protein 6	0.889151498	up	0.009763
TNFRSF10B	tumor necrosis factor receptor superfamily, member 10b	1.050105215	up	1.06E-12
TNFRSF10D	tumor necrosis factor receptor superfamily, member 10d, decoy with truncated death domain	1.168896674	up	1.38E-11
TNFRSF1B	tumor necrosis factor receptor superfamily, member 1B	2.164252738	up	0.025187
TNFRSF21	tumor necrosis factor receptor superfamily, member 21	0.710804117	up	5.33E-06
TNFRSF9	tumor necrosis factor receptor superfamily, member 9	2.027215372	up	7.24E-12
TNFSF12	tumor necrosis factor (ligand) superfamily, member 12	0.703609251	up	0.001878
TNFSF12-TNFSF13	TNFSF12-TNFSF13 readthrough	0.736771739	up	0.013412
TNFSF9	tumor necrosis factor (ligand) superfamily, member 9	0.762644805	up	0.000959
TNIP1	TNFAIP3 interacting protein 1	1.051701017	up	1.49E-08
TNIP2	TNFAIP3 interacting protein 2	0.639803061	up	0.00036
TNS1	tensin 1	0.768416075	up	0.000149
TNS4	tensin 4	-0.91138279	down	2.12E-09

TNXB	tenascin XB	0.781467125	up	0.003489
TOB2	transducer of ERBB2, 2	-0.6826251	down	0.000308
TOM1	target of myb1 (chicken)	1.293397803	up	1.53E-15
TOMM20	translocase of outer mitochondrial membrane 20 homolog (yeast)	-1.07888625	down	1.33E-12
TOMM34	translocase of outer mitochondrial membrane 34	-0.94974139	down	5.74E-08
TOMM40	translocase of outer mitochondrial membrane 40 homolog (yeast)	-0.83108213	down	9.19E-07
TOMM6	translocase of outer mitochondrial membrane 6 homolog (yeast)	-0.763943	down	2.34E-06
TOP1	topoisomerase (DNA) I	0.691947015	up	2.16E-05
TOP2A	topoisomerase (DNA) II alpha 170kDa	-1.57860371	down	4.77E-28
TOP2B	topoisomerase (DNA) II beta 180kDa	-0.84657245	down	6.02E-08
TOPBP1	topoisomerase (DNA) II binding protein 1	-1.34073143	down	1.27E-14
TPBG	trophoblast glycoprotein	0.761471374	up	1.85E-06
TPM4	tropomyosin 4	-1.12288784	down	1.96E-14
TPP1	tripeptidyl peptidase I	0.823348754	up	1.42E-07
TPPP	tubulin polymerization promoting protein	0.600900328	up	0.001052
TPPP3	tubulin polymerization-promoting protein family member 3	0.973795997	up	0.000103
TPRA1	transmembrane protein, adipocyte asscociated 1	1.273360594	up	7.21E-12
TPRG1	tumor protein p63 regulated 1	1.284650647	up	0.032439
TPRG1L	tumor protein p63 regulated 1-like	1.226511868	up	3.78E-14
TPRN	taperin	0.708759919	up	0.0201
TPX2	TPX2, microtubule-associated	-0.72380292	down	3.23E-06
TRA2B	transformer 2 beta homolog (Drosophila)	-1.05165289	down	1.63E-11

TRAF3IP2	TRAF3 interacting protein 2	0.876168487	up	8.61E-07
TRAF5	TNF receptor-associated factor 5	-0.94673677	down	0.000103
TRANK1	tetratricopeptide repeat and ankyrin repeat containing 1	1.405373954	up	1.57E-11
TRAPPC6B	trafficking protein particle complex 6B	-0.60480268	down	0.014756
TRAPPC8	trafficking protein particle complex 8	-0.82286942	down	0.000138
TREX1	three prime repair exonuclease 1	1.121035709	up	2.52E-06
TRIB1	tribbles pseudokinase 1	1.147859885	up	1.02E-13
TRIM11	tripartite motif containing 11	0.817013889	up	0.000459
TRIM35	tripartite motif containing 35	0.616758816	up	0.00179
TRIM36	tripartite motif containing 36	1.191691965	up	0.003071
TRIM44	tripartite motif containing 44	-1.24213739	down	2.8E-15
TRIM46	tripartite motif containing 46	0.789227021	up	0.008458
TRIP10	thyroid hormone receptor interactor 10	0.685785427	up	0.000854
TRIP11	thyroid hormone receptor interactor 11	-0.98890384	down	0.009767
TRIP13	thyroid hormone receptor interactor 13	-1.09536999	down	4.36E-11
TRMT2B	tRNA methyltransferase 2 homolog B (S. cerevisiae)	-0.63459375	down	0.001479
TRMT6	tRNA methyltransferase 6 homolog (S. cerevisiae)	-0.84536121	down	6.8E-05
TRO	trophinin	2.679553131	up	0.011479
TRPV1	transient receptor potential cation channel, subfamily V, member 1	0.602236665	up	0.036725
TRPV2	transient receptor potential cation channel, subfamily V, member 2	0.995605213	up	0.005539
TSC22D3	TSC22 domain family, member 3	1.180758994	up	0.038085
TSC22D4	TSC22 domain family, member 4	0.654765349	up	0.00023
TSNARE1	t-SNARE domain containing 1	3.548888163	up	0.002663
TSNAX	translin-associated factor X	-0.70576374	down	0.04857

				5
TSPAN13	tetraspanin 13	-1.08117059	down	2.23E-09
TSPAN14	tetraspanin 14	-0.7381615	down	2.11E-06
TTC14	tetratricopeptide repeat domain 14	-0.68810617	down	0.007373
TTC17	tetratricopeptide repeat domain 17	0.702545171	up	0.000137
TTC21A	tetratricopeptide repeat domain 21A	0.587806037	up	0.048231
TTC25	tetratricopeptide repeat domain 25	0.772734382	up	0.016292
TTC26	tetratricopeptide repeat domain 26	-1.44620255	down	5.94E-05
TTC30A	tetratricopeptide repeat domain 30A	-0.73610288	down	0.022695
TTC32	tetratricopeptide repeat domain 32	-0.83506597	down	0.005091
TTC33	tetratricopeptide repeat domain 33	-1.43280404	down	8.06E-10
TTC9B	tetratricopeptide repeat domain 9B	20	up	0.033505
TTF2	transcription termination factor, RNA polymerase II	-1.00095962	down	4.32E-06
TTLL11	tubulin tyrosine ligase-like family, member 11	1.052517792	up	0.004867
TTPAL	tocopherol (alpha) transfer protein-like	-0.99653647	down	2.25E-06
TTYH2	tweety family member 2	1.286415998	up	3.79E-11
TUBA1B	tubulin, alpha 1b	-1.24315055	down	3.78E-09
TUBA1C	tubulin, alpha 1c	-0.61186117	down	6.61E-05
TUBB2A	tubulin, beta 2A class IIa	0.604966648	up	0.002073
TUBB2B	tubulin, beta 2B class IIb	0.689299732	up	0.001359
TUBB3	tubulin, beta 3 class III	0.637305742	up	4.75E-05
TUBB4A	tubulin, beta 4A class IVa	-0.77620632	down	0.000286

TUBB4B	tubulin, beta 4B class IVb	-1.02388044	down	7.31E-10
TUBB6	tubulin, beta 6 class V	-0.68171134	down	4.25E-05
TUFT1	tuftelin 1	1.312062221	up	0.006298
TUT1	terminal uridylyl transferase 1, U6 snRNA-specific	0.594576411	up	0.002818
TWIST2	twist family bHLH transcription factor 2	-1.91184368	down	1.18E-16
TWISTNB	TWIST neighbor	-0.64595404	down	0.001027
TWSG1	twisted gastrulation BMP signaling modulator 1	-0.73097517	down	3.72E-05
TXNDC15	thioredoxin domain containing 15	-0.73625717	down	0.000118
TXNDC17	thioredoxin domain containing 17	-0.59885389	down	0.00027
TXNDC5	thioredoxin domain containing 5 (endoplasmic reticulum)	-1.05011704	down	0.000391
TYMP	thymidine phosphorylase	1.272350107	up	3.14E-13
TYMS	thymidylate synthetase	-1.2848545	down	2.13E-14
TYW3	tRNA-yW synthesizing protein 3 homolog (S. cerevisiae)	-0.94437616	down	4.24E-05
U2AF2	U2 small nuclear RNA auxiliary factor 2	-0.91715656	down	7.01E-05
U2SURP	U2 snRNP-associated SURP domain containing	-0.61763732	down	0.000191
UAP1L1	UDP-N-acetylglucosamine pyrophosphorylase 1 like 1	0.937993614	up	1.38E-07
UBC	ubiquitin C	1.113767946	up	0.004851
UBE2C	ubiquitin-conjugating enzyme E2C	-0.74988768	down	3.09E-06
UBE2E1	ubiquitin-conjugating enzyme E2E 1	0.819800351	up	1.33E-06
UBE2E2	ubiquitin-conjugating enzyme E2E 2	0.94623289	up	1.93E-05
UBE2G2	ubiquitin-conjugating enzyme E2G 2	-1.07994132	down	1.89E-10
UBE2L6	ubiquitin-conjugating enzyme E2L 6	0.829121011	up	9.02E-06

UBE2O	ubiquitin-conjugating enzyme E2O	0.673281215	up	2.53E-05
UBE2T	ubiquitin-conjugating enzyme E2T (putative)	-0.91492314	down	0.000287
UBE3B	ubiquitin protein ligase E3B	-0.95977712	down	1.17E-08
UBE4A	ubiquitination factor E4A	-0.62634523	down	0.007906
UBTF	upstream binding transcription factor, RNA polymerase I	-0.68814302	down	0.0038
UCKL1	uridine-cytidine kinase 1-like 1	0.86799601	up	0.000169
UCP2	uncoupling protein 2 (mitochondrial, proton carrier)	-0.59332416	down	0.000959
UGCG	UDP-glucose ceramide glucosyltransferase	1.371502797	up	0.00228
UGDH	UDP-glucose 6-dehydrogenase	-0.61467585	down	0.000124
UGGT1	UDP-glucose glycoprotein glucosyltransferase 1	-0.95258753	down	0.000111
UGT1A1	UDP glucuronosyltransferase 1 family, polypeptide A1	-1.09535817	down	0.007604
UHRF1	ubiquitin-like with PHD and ring finger domains 1	-1.90088621	down	1.54E-08
ULK2	unc-51 like autophagy activating kinase 2	-0.63425129	down	0.000557
UNC119	unc-119 homolog (C. elegans)	0.61518492	up	0.000285
UNC5A	unc-5 homolog A (C. elegans)	1.307664332	up	0.015227
UNG	uracil-DNA glycosylase	-0.87471976	down	7.75E-07
UPK1B	uroplakin 1B	-0.85041514	down	2.26E-07
UPP1	uridine phosphorylase 1	0.918522418	up	0.001344
UPRT	uracil phosphoribosyltransferase (FUR1) homolog (S. cerevisiae)	-1.20116466	down	3.1E-06
UQCC1	ubiquinol-cytochrome c reductase complex assembly factor 1	-1.00114671	down	3.35E-09
UQCRFS1	ubiquinol-cytochrome c reductase, Rieske iron-sulfur polypeptide 1	-0.79981303	down	1.8E-07
USE1	unconventional SNARE in the ER 1 homolog (S. cerevisiae)	0.744468008	up	0.00041
USH1C	Usher syndrome 1C (autosomal recessive, severe)	-0.91810158	down	0.021225
USH1G	Usher syndrome 1G (autosomal recessive)	3.468392803	up	0.001813

USP1	ubiquitin specific peptidase 1	-1.3978175	down	1.55E-12
USP13	ubiquitin specific peptidase 13 (isopeptidase T-3)	-1.59275198	down	3.29E-14
USP24	ubiquitin specific peptidase 24	-0.62435917	down	0.000441
USP3	ubiquitin specific peptidase 3	0.706717328	up	1.9E-05
USP35	ubiquitin specific peptidase 35	0.678645487	up	0.000822
USP40	ubiquitin specific peptidase 40	-1.28077617	down	2.77E-12
USP45	ubiquitin specific peptidase 45	-0.88378272	down	0.001859
UTP20	UTP20, small subunit (SSU) processome component, homolog (yeast)	-0.90745363	down	0.000108
VASN	vasorin	0.616820264	up	0.032638
VAT1	vesicle amine transport 1	0.731659541	up	8.82E-07
VAV1	vav 1 guanine nucleotide exchange factor	0.910323048	up	6.19E-06
VAV3	vav 3 guanine nucleotide exchange factor	-1.01534622	down	1.84E-06
VCP	valosin containing protein	-0.61901277	down	4.84E-05
VCPKMT	valosin containing protein lysine (K) methyltransferase	0.688752134	up	0.04069
VDAC3	voltage-dependent anion channel 3	-1.04094829	down	1.07E-08
VDR	vitamin D (1,25- dihydroxyvitamin D3) receptor	1.687597813	up	4.23E-20
VEGFA	vascular endothelial growth factor A	1.027174022	up	0.002627
VEGFC	vascular endothelial growth factor C	0.620864157	up	0.002418
VGF	VGF nerve growth factor inducible	1.015121595	up	0.003488
VIM	vimentin	0.677309217	up	9.42E-06
VNN1	vanin 1	1.941803828	up	0.002548
VNN3	vanin 3	3.670977738	up	0.002836
VPS11	vacuolar protein sorting 11 homolog (S. cerevisiae)	0.863803378	up	4.37E-0

				7
VPS13A	vacuolar protein sorting 13 homolog A (S. cerevisiae)	-1.05143921	down	0.004083
VPS18	vacuolar protein sorting 18 homolog (S. cerevisiae)	0.658030221	up	0.000147
VPS37D	vacuolar protein sorting 37 homolog D (S. cerevisiae)	0.752055622	up	0.027358
VPS45	vacuolar protein sorting 45 homolog (S. cerevisiae)	-0.72147362	down	0.000261
VPS52	vacuolar protein sorting 52 homolog (S. cerevisiae)	-1.50358182	down	1.22E-14
VPS9D1	VPS9 domain containing 1	0.738436787	up	0.000922
VRK1	vaccinia related kinase 1	-1.33894023	down	1.81E-10
VRK3	vaccinia related kinase 3	1.008274825	up	2.61E-10
VSIG10	V-set and immunoglobulin domain containing 10	-0.5996759	down	0.005577
VTN	vitronectin	-0.70833212	down	0.013995
VWA1	von Willebrand factor A domain containing 1	-1.12149356	down	1.61E-08
VWA5B2	von Willebrand factor A domain containing 5B2	0.779021016	up	0.04664
VWA7	von Willebrand factor A domain containing 7	0.897220547	up	0.001843
WBP2	WW domain binding protein 2	0.86368271	up	2.59E-08
WBSCR28	Williams-Beuren syndrome chromosome region 28	1.692229747	up	0.003792
WDFY1	WD repeat and FYVE domain containing 1	0.731559714	up	1.94E-05
WDHD1	WD repeat and HMG-box DNA binding protein 1	-1.29039899	down	3.69E-11
WDR1	WD repeat domain 1	-1.11445807	down	3.61E-13
WDR25	WD repeat domain 25	1.028508715	up	0.000663
WDR34	WD repeat domain 34	-0.71557315	down	6.57E-05
WDR35	WD repeat domain 35	-0.78912712	down	1.86E-05
WDR4	WD repeat domain 4	-1.45000742	down	5.17E-0

				5
WDR5B	WD repeat domain 5B	-0.59769397	down	0.02359 6
WDR62	WD repeat domain 62	-1.32450098	down	1.58E-1 4
WDR76	WD repeat domain 76	-1.25108875	down	3.66E-0 9
WDR92	WD repeat domain 92	-0.76696666	down	0.00078 6
WIPF3	WAS/WASL interacting protein family, member 3	1.327547644	up	0.00020 8
WIPI1	WD repeat domain, phosphoinositide interacting 1	0.779337353	up	0.00192 9
WISP2	WNT1 inducible signaling pathway protein 2	0.920544917	up	4.82E-0 6
WNT3	wingless-type MMTV integration site family, member 3	-0.70349467	down	0.00307 1
WNT4	wingless-type MMTV integration site family, member 4	0.73833949	up	0.04254 9
WNT7A	wingless-type MMTV integration site family, member 7A	20	up	0.04266 5
WRAP53	WD repeat containing, antisense to TP53	-0.64861009	down	0.00134 1
WRB	tryptophan rich basic protein	-0.67514129	down	0.00118 3
WRN	Werner syndrome, RecQ helicase-like	-0.72457981	down	0.00501 8
WSCD1	WSC domain containing 1	0.938577622	up	1.74E-0 6
WTAP	Wilms tumor 1 associated protein	1.026106943	up	0.00080 9
WWC3	WWC family member 3	-0.62141014	down	0.00043 3
WWTR1	WW domain containing transcription regulator 1	-1.15367473	down	1.18E-1 3
XDH	xanthine dehydrogenase	1.687854581	up	4.74E-1 1
XKR8	XK, Kell blood group complex subunit-related family, member 8	0.991013163	up	2.19E-0 6
XPO1	exportin 1	-0.75916852	down	8.83E-0 7
XPO4	exportin 4	-0.61757863	down	0.01264 3

XRCC1	X-ray repair complementing defective repair in Chinese hamster cells 1	0.661911385	up	9.26E-05
XRCC2	X-ray repair complementing defective repair in Chinese hamster cells 2	-1.11030218	down	0.004625
XRCC3	X-ray repair complementing defective repair in Chinese hamster cells 3	-1.24583623	down	3.74E-09
XRCC4	X-ray repair complementing defective repair in Chinese hamster cells 4	-1.23842111	down	0.004758
XRN1	5'-3' exoribonuclease 1	-0.62338246	down	0.007538
YIPF1	Yip1 domain family, member 1	-0.59314829	down	0.009548
YIPF2	Yip1 domain family, member 2	0.646117891	up	0.001738
YPEL1	yippee-like 1 (Drosophila)	-1.03733506	down	0.001612
YPEL3	yippee-like 3 (Drosophila)	0.773350267	up	4.48E-05
YPEL4	yippee-like 4 (Drosophila)	1.873047375	up	0.001439
YRDC	yrdC N(6)-threonylcarbamoyltransferase domain containing	0.63706047	up	0.001832
YTHDC2	YTH domain containing 2	-0.67126333	down	0.000467
YWHAE	tyrosine 3-monooxygenase/tryptophan 5-monooxygenase activation protein, epsilon	-0.60121895	down	0.000127
YY1AP1	YY1 associated protein 1	0.593431731	up	0.000388
ZBED8	zinc finger, BED-type containing 8	-1.03465751	down	0.010614
ZBTB1	zinc finger and BTB domain containing 1	-0.69708093	down	0.000992
ZBTB12	zinc finger and BTB domain containing 12	-1.2181502	down	0.001623
ZBTB14	zinc finger and BTB domain containing 14	-0.72617502	down	0.003926
ZBTB33	zinc finger and BTB domain containing 33	-0.77785701	down	0.000986
ZBTB34	zinc finger and BTB domain containing 34	0.804202324	up	6.1E-05
ZBTB37	zinc finger and BTB domain containing 37	-0.9113132	down	0.03402
ZBTB38	zinc finger and BTB domain containing 38	-0.75989943	down	1.75E-05
ZBTB43	zinc finger and BTB domain containing 43	1.235493117	up	7.67E-0

				7
ZBTB44	zinc finger and BTB domain containing 44	-0.73681784	down	0.000726
ZBTB46	zinc finger and BTB domain containing 46	0.903849943	up	0.002822
ZBTB48	zinc finger and BTB domain containing 48	0.606753208	up	0.006676
ZC3H11A	zinc finger CCCH-type containing 11A	-1.19495897	down	0.004868
ZC3H12A	zinc finger CCCH-type containing 12A	0.965510151	up	0.032754
ZC3H12C	zinc finger CCCH-type containing 12C	0.672541275	up	0.042208
ZC3H13	zinc finger CCCH-type containing 13	-0.82774993	down	2.32E-06
ZC3H14	zinc finger CCCH-type containing 14	-0.62939819	down	0.000308
ZC3H15	zinc finger CCCH-type containing 15	0.77942464	up	0.037847
ZC3H3	zinc finger CCCH-type containing 3	0.622024614	up	0.000903
ZC3H4	zinc finger CCCH-type containing 4	-0.64434303	down	0.037162
ZC3H8	zinc finger CCCH-type containing 8	1.070768527	up	2.85E-05
ZC3HAV1	zinc finger CCCH-type, antiviral 1	-0.61056141	down	0.011808
ZC3HC1	zinc finger, C3HC-type containing 1	-0.61310279	down	0.008675
ZC4H2	zinc finger, C4H2 domain containing	1.078920382	up	0.000108
ZCCHC14	zinc finger, CCHC domain containing 14	-0.82303032	down	3.94E-05
ZCCHC3	zinc finger, CCHC domain containing 3	-0.86519456	down	1.59E-06
ZCCHC9	zinc finger, CCHC domain containing 9	0.586786535	up	0.007259
ZCRB1	zinc finger CCHC-type and RNA binding motif 1	-0.88385931	down	0.000503
ZDBF2	zinc finger, DBF-type containing 2	-1.01594354	down	0.000241
ZDHHC11B	zinc finger, DHHC-type containing 11B	1.047747547	up	3.86E-06

ZDHHC18	zinc finger, DHHC-type containing 18	0.683749207	up	3.29E-05
ZEB2	zinc finger E-box binding homeobox 2	1.262121033	up	0.005881
ZER1	zyg-11 related, cell cycle regulator	0.713092283	up	1.29E-05
ZFAND2A	zinc finger, AN1-type domain 2A	1.26243693	up	1.31E-11
ZFC3H1	zinc finger, C3H1-type containing	-0.75583347	down	0.001445
ZFP36	ZFP36 ring finger protein	2.02387719	up	0.004961
ZFP36L1	ZFP36 ring finger protein-like 1	1.686997053	up	4.36E-11
ZFP41	ZFP41 zinc finger protein	-0.60875832	down	0.011898
ZFR	zinc finger RNA binding protein	0.667601193	up	0.000355
ZFX	zinc finger protein, X-linked	-0.95777413	down	0.000409
ZFY	zinc finger protein, Y-linked	-1.00664348	down	0.018126
ZFYVE1	zinc finger, FYVE domain containing 1	0.761812156	up	1.61E-05
ZFYVE16	zinc finger, FYVE domain containing 16	-0.76230363	down	0.004343
ZFYVE26	zinc finger, FYVE domain containing 26	-0.84805694	down	0.006083
ZGRF1	zinc finger, GRF-type containing 1	-1.61514252	down	0.002435
ZHX1	zinc fingers and homeoboxes 1	-0.69712173	down	0.001445
ZHX2	zinc fingers and homeoboxes 2	0.742052734	up	0.00297
ZHX3	zinc fingers and homeoboxes 3	0.635136703	up	0.006851
ZMAT5	zinc finger, matrin-type 5	0.637652022	up	0.001527
ZMIZ2	zinc finger, MIZ-type containing 2	0.632541102	up	8.68E-05
ZMPSTE24	zinc metallopeptidase STE24	-0.96155044	down	2.31E-05
ZMYM3	zinc finger, MYM-type 3	-0.82673242	down	1.11E-06

ZMYM5	zinc finger, MYM-type 5	1.025992287	up	0.000144
ZMYND11	zinc finger, MYND-type containing 11	-2.12437764	down	0.022019
ZNF12	zinc finger protein 12	-0.66950855	down	0.023725
ZNF148	zinc finger protein 148	-0.66855879	down	0.004585
ZNF19	zinc finger protein 19	0.833514576	up	0.033081
ZNF197	zinc finger protein 197	0.889774195	up	0.000177
ZNF2	zinc finger protein 2	1.139243424	up	7.24E-06
ZNF205	zinc finger protein 205	0.661944196	up	0.016869
ZNF211	zinc finger protein 211	0.707751008	up	0.01287
ZNF217	zinc finger protein 217	0.715949974	up	3.5E-06
ZNF222	zinc finger protein 222	0.926363669	up	0.005305
ZNF229	zinc finger protein 229	0.903932164	up	0.000843
ZNF230	zinc finger protein 230	0.899342755	up	0.001045
ZNF233	zinc finger protein 233	1.987305312	up	0.004738
ZNF235	zinc finger protein 235	-1.17430624	down	0.018623
ZNF239	zinc finger protein 239	0.971884097	up	0.041067
ZNF250	zinc finger protein 250	0.636955126	up	0.006977
ZNF260	zinc finger protein 260	-0.77922123	down	0.002449
ZNF274	zinc finger protein 274	0.802063618	up	5.44E-06
ZNF280A	zinc finger protein 280A	0.962378389	up	0.001491
ZNF282	zinc finger protein 282	0.765911882	up	3.94E-06
ZNF296	zinc finger protein 296	1.252625777	up	2.22E-05
ZNF317	zinc finger protein 317	0.661542978	up	0.00012

				1
ZNF319	zinc finger protein 319	0.658292629	up	0.000458
ZNF324	zinc finger protein 324	1.404410115	up	1.23E-10
ZNF324B	zinc finger protein 324B	0.753309061	up	0.014142
ZNF326	zinc finger protein 326	-0.96466484	down	0.000202
ZNF329	zinc finger protein 329	0.690303558	up	0.032336
ZNF34	zinc finger protein 34	0.756010776	up	0.004425
ZNF354A	zinc finger protein 354A	0.697230387	up	0.008551
ZNF362	zinc finger protein 362	-0.85378264	down	0.045829
ZNF367	zinc finger protein 367	-1.03278976	down	2.55E-05
ZNF37A	zinc finger protein 37A	-0.68169527	down	0.017354
ZNF383	zinc finger protein 383	0.743652941	up	0.004429
ZNF385A	zinc finger protein 385A	0.973349483	up	9.51E-05
ZNF394	zinc finger protein 394	0.628478035	up	0.00269
ZNF407	zinc finger protein 407	-0.71962564	down	0.028915
ZNF416	zinc finger protein 416	0.848045143	up	0.011504
ZNF426	zinc finger protein 426	0.940301261	up	0.002646
ZNF461	zinc finger protein 461	0.635088685	up	0.042755
ZNF467	zinc finger protein 467	0.808488688	up	0.004531
ZNF469	zinc finger protein 469	-1.40729728	down	0.002856
ZNF473	zinc finger protein 473	0.639959964	up	0.016416
ZNF488	zinc finger protein 488	-1.00112781	down	4.88E-06
ZNF512B	zinc finger protein 512B	0.683120961	up	0.00017

				6
ZNF517	zinc finger protein 517	0.701536855	up	0.01585<br>3
ZNF518A	zinc finger protein 518A	-0.68359103	down	0.00843<br>6
ZNF526	zinc finger protein 526	0.854225264	up	0.00068<br>8
ZNF550	zinc finger protein 550	0.948158872	up	0.00062<br>9
ZNF555	zinc finger protein 555	0.949619758	up	0.00061
ZNF557	zinc finger protein 557	0.730815502	up	0.01657<br>4
ZNF568	zinc finger protein 568	1.810211149	up	0.03797<br>6
ZNF57	zinc finger protein 57	0.838259518	up	0.02398<br>6
ZNF581	zinc finger protein 581	1.049781656	up	8.85E-0<br>7
ZNF605	zinc finger protein 605	-1.00166269	down	0.00010<br>2
ZNF622	zinc finger protein 622	0.644878244	up	0.00028<br>5
ZNF638	zinc finger protein 638	-0.69315307	down	0.00376
ZNF641	zinc finger protein 641	1.11716895	up	2.26E-0<br>9
ZNF652	zinc finger protein 652	-0.77237374	down	5.35E-0<br>5
ZNF697	zinc finger protein 697	1.149147231	up	6.09E-0<br>5
ZNF789	zinc finger protein 789	-0.99072415	down	0.00054<br>2
ZNF79	zinc finger protein 79	0.729510549	up	0.00924<br>1
ZNF821	zinc finger protein 821	0.671284242	up	0.00753<br>3
ZNF852	zinc finger protein 852	-1.20189267	down	0.03091<br>7
ZNF880	zinc finger protein 880	3.991634362	up	0.01171<br>6
ZP1	zona pellucida glycoprotein 1 (sperm receptor)	1.951382916	up	0.00333<br>1
ZSCAN16	zinc finger and SCAN domain containing 16	0.841191434	up	0.00428<br>4

ZSCAN18	zinc finger and SCAN domain containing 18	1.449455608	up	0.006482
ZSCAN21	zinc finger and SCAN domain containing 21	0.710711628	up	0.000878
ZWILCH	zwilch kinetochore protein	-1.61502128	down	3.03E-13
ZWINT	ZW10 interacting kinetochore protein	-1.50526926	down	3.08E-09
ZXDC	ZXD family zinc finger C	0.768115107	up	3.47E-05
ZYG11A	zyg-11 family member A, cell cycle regulator	-0.71435147	down	0.008064
ZYX	zyxin	0.762698689	up	8.33E-05

Supplementary Table S2:The list of primers and siRNA sequence.

GENE	Forward primer	Reverse primer
**human qRT-PCR**		
**LINC00662**	GGGTTAGACCTCGCACAGG	CGCCTTTCACAGAACTGAGG
**P57**	CTAGCCAGCAGGCATCGAG	GTGGTGGACTCTTCTGCGTC
**CDK1**	GGGTCAGCTCGCTACTCAAC	AAGTTTTTGACGTGGGATGC
**CDH2**	GTCAGCAGAAGTTGAAGAAATAGTG	GCAAGTTGATTGGAGGGATG
**E2F1**	GCCACTGACTCTGCCACCATAG	CTGCCCATCCGGGACAAC
**BIK**	GACCATGGAGGTTCTTGGCA	GACCATGGAGGTTCTTGGCA
Sequences for siRNAs		
**si-LINC00662 1#**	UAAAGCGUGCCUACACGCUUCUGAA	UAAAGCGUGCCUACACGCUUCUGAA
**si-LINC00662 2#**	GCAGACACCUUUGCCUUGUUCCUGA	UCAGGAACAAGGCAAAGGUGUCUGC
**si-EZH2**	GAGGUUCAGACGAGCUGAUUU	AAAUCAGCUCGUCUGAACCUC
